# Synergistic induction of mitotic pyroptosis and tumor remission by inhibiting proteasome and WEE family kinases

**DOI:** 10.1038/s41392-024-01896-z

**Published:** 2024-07-12

**Authors:** Zhan-Li Chen, Chen Xie, Wei Zeng, Rui-Qi Huang, Jin-E Yang, Jin-Yu Liu, Ya-Jing Chen, Shi-Mei Zhuang

**Affiliations:** 1 MOE Key Laboratory of Gene Function and Regulation, Guangdong Province Key Laboratory of Pharmaceutical Functional Genes, School of Life Sciences, State Key Laboratory of Oncology in Southern China, Sun Yat-sen University, Guangzhou, PR China; 2 Key Laboratory of Liver Disease of Guangdong Province, The Third Affiliated Hospital, Sun Yat-sen University, Guangzhou, 510630 PR China

**Keywords:** Cancer therapy, Cell biology

## Abstract

Mitotic catastrophe (MC), which occurs under dysregulated mitosis, represents a fascinating tactic to specifically eradicate tumor cells. Whether pyroptosis can be a death form of MC remains unknown. Proteasome-mediated protein degradation is crucial for M-phase. Bortezomib (BTZ), which inhibits the 20S catalytic particle of proteasome, is approved to treat multiple myeloma and mantle cell lymphoma, but not solid tumors due to primary resistance. To date, whether and how proteasome inhibitor affected the fates of cells in M-phase remains unexplored. Here, we show that BTZ treatment, or silencing of PSMC5, a subunit of 19S regulatory particle of proteasome, causes G2- and M-phase arrest, multi-polar spindle formation, and consequent caspase-3/GSDME-mediated pyroptosis in M-phase (designated as mitotic pyroptosis). Further investigations reveal that inhibitor of WEE1/PKMYT1 (PD0166285), but not inhibitor of ATR, CHK1 or CHK2, abrogates the BTZ-induced G2-phase arrest, thus exacerbates the BTZ-induced mitotic arrest and pyroptosis. Combined BTZ and PD0166285 treatment (named BP-Combo) selectively kills various types of solid tumor cells, and significantly lessens the IC50 of both BTZ and PD0166285 compared to BTZ or PD0166285 monotreatment. Studies using various mouse models show that BP-Combo has much stronger inhibition on tumor growth and metastasis than BTZ or PD0166285 monotreatment, and no obvious toxicity is observed in BP-Combo-treated mice. These findings disclose the effect of proteasome inhibitors in inducing pyroptosis in M-phase, characterize pyroptosis as a new death form of mitotic catastrophe, and identify dual inhibition of proteasome and WEE family kinases as a promising anti-cancer strategy to selectively kill solid tumor cells.

## Introduction

Mitotic catastrophe (MC) occurs under aberrant mitosis and often leads to cell death either during or shortly after dysregulated mitosis.^1,2^ It is accompanied by a prolonged mitotic duration, termed mitotic arrest. MC can be triggered by various stimuli, including extensive DNA damage, premature mitotic entry, mitotic apparatus perturbation, and mitotic checkpoint defects.^3^ Tumor-specific alterations, like tetraploidy, aneuploidy and centrosome amplification, may render cancer cells more prone to mitotic aberrations and hence more sensitive to MC than normal cells.^4–6^

The reported modes of cell death under mitotic catastrophe have been attributed to intrinsic apoptosis.^2,7,8^ To date, whether pyroptosis, a recently defined programmed necrotic cell death, is involved in MC remains unreported. Pyroptosis is specifically mediated by gasdermin family, including GSDMA, GSDMB, GSDMC, GSDMD, GSDME and DFNB59.^9^ Except DFNB59, gasdermins share highly conserved N-terminal and C-terminal domains separated by a variable linker. Once the linker is cleaved, the N-terminal domain binds to the acidic phospholipids of cytoplasmic membranes and forms membrane pores,^10,11^ which disrupts the osmotic potential and results in characteristic morphology and biochemical features, like cell swelling with large ballooning bubbles, double-staining for annexin V and propidium iodide (PI), and release of lactate dehydrogenase (LDH).^12^

During mitosis phase (M-phase), both transcription^13,14^ and translation^15,16^ of mRNAs are globally repressed, the proteasome-mediated protein degradation thus plays important roles in regulating M-phase.^17^ The proteasome consists of the 20S catalytic particle (20S-CP) and the 19S regulatory particle (19S-RP).^18,19^ The β1, β2 and β5 subunits of the 20S-CP possess proteolytic activities, and β5 subunit has been identified as a rate-limiting effector and a primary target of clinically available proteasome inhibitors.^20,21^ Cancer cells are addicted to enhanced proteasome activity for efficient protein turnover to support their survival and proliferation, and thus may be sensitive to the treatment of proteasome inhibitor.^22–24^ Bortezomib (BTZ), which binds reversibly to β5 subunit and inhibits proteasome activity, is the first proteasome inhibitor approved to treat human hematologic cancers, like multiple myeloma and mantle cell lymphoma.^25,26^ However, primary resistance to proteasome inhibitors remains a big challenge for their clinic uses in solid tumors.^25,27^ On the other hand, the heterohexameric AAA+ ATPase ring of the 19S-RP, consisting of PSMC1, PSMC2, PSMC3, PSMC4, PSMC5 and PSMC6, plays essential roles in unfolding and delivering the proteasomal substrates into the proteolytically active sites.^28,29^ Nevertheless, there still lacks effective inhibitors for these AAA+ ATPases of 19S-RP.^30^ To date, whether proteasome inhibition can elicit pyroptosis has not been reported yet. It is suggested that BTZ can induce cell cycle arrest at G2/M phase based on increased proportion of 4N cells,^31,32^ which cannot distinguish the M-phase from G2-phase cells. Whether and how proteasome inhibitor affects the fates of cells in M-phase remain unexplored. Moreover, the suggestion that BTZ triggers MC is based on abnormal karyotype^33^ without analyzing the mitosis process of living cells by using high-throughput video microscopy or time-lapse fluorescence microscopy and co-staining of mitotic marker with the indicator of cell death. Obviously, the roles of proteasome inhibitor in M-phase and MC required further characterization.

The G2/M phase arrest is regulated by checkpoint kinase 1/2 (CHK1/2) and WEE family kinases.^34^ Unfortunately, inhibitors of CHK1/2 and WEE family kinases display high-grade hematological toxicity and low efficacy, leading to termination of further clinical development.^35,36^ ATR is an apical kinase of the CHK1/2-WEE1 pathway and some ATR inhibitors have better toxicity profiles.^36^ Whether inhibitors of these kinases can synergize with proteasome inhibitor to trigger mitotic abnormality, pyroptosis and MC, or to lower drug toxicity, is still unknown.

In this study, we found that proteasome inhibition by BTZ treatment or PSMC5 silencing resulted in abnormal mitosis and subsequent GSDME-mediated pyroptosis in M-phase. In addition, BTZ treatment upregulated WEE family kinases (WEE1 and PKMYT1) and CHK1, and caused cell cycle arrest at G2-phase. PD0166285,^37–39^ the inhibitor of WEE1 and PKMYT1, but not the inhibitor of ATR or CHK1/2, efficiently abrogated BTZ-induced G2-phase arrest, and exacerbated BTZ-induced pyroptosis. Furthermore, the combined BTZ and PD0166285 treatment (designated as BP-Combo) displayed synergistic effect in selectively killing various types of solid tumor cells in vitro and significantly lessened the IC50 of both BTZ and PD0166285. Consistently, multiple in vivo models revealed that BP-Combo could suppress tumor growth and metastasis, and prolong the survival of tumor-bearing mice with low toxicity. These findings disclose the regulatory role of proteasome inhibitor in mitotic pyroptosis, and identify the combined inhibition of proteasome and WEE family kinases as an attractive anti-cancer strategy that lowers the drug doses and has better safety profiles as well as more potent anti-tumor efficacies.

## Results

### Inhibition of proteasome triggers M-phase arrest and multi-polar spindle formation

**Fig. 1:**
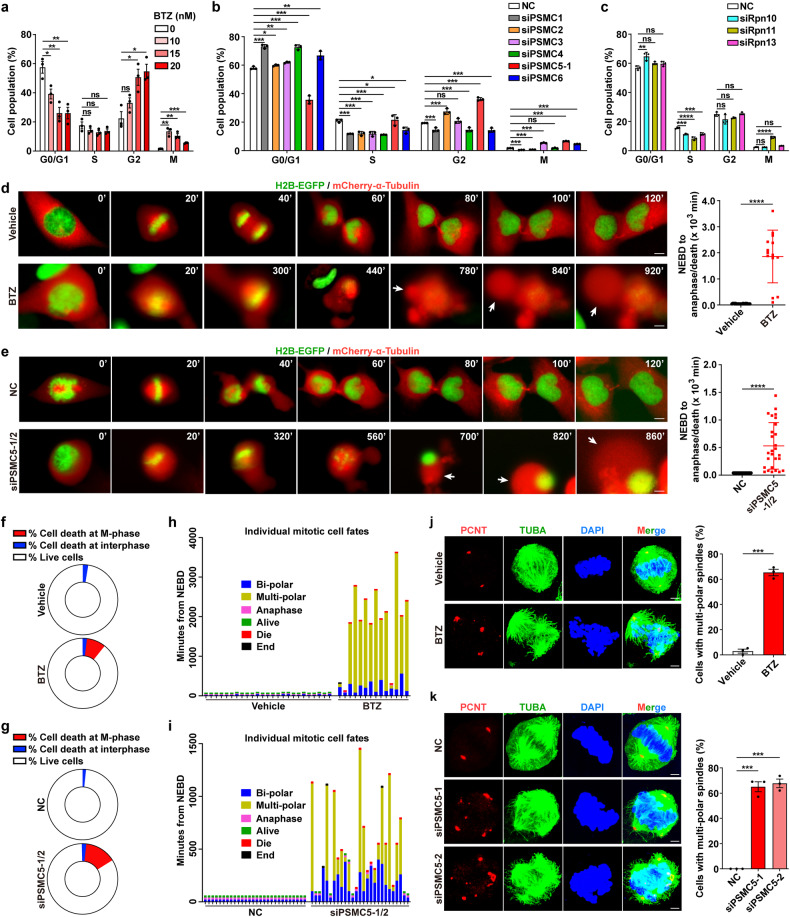
Inhibition of proteasome induces M-phase arrest, multi-polar spindle formation and mitotic catastrophe. **a**–**c** The effects of proteasome inhibition on cell cycle progression. SNU449 cells were treated with the indicated dose of BTZ (**a**) for 30 h or transfected with the indicated RNA duplexes for 60 h (**b**, **c**), then stained for Ser-10-phosphorylated histone H3 (pH3-S10) to indicate M-phase cells and stained with propidium iodide (PI) to indicate DNA content, followed by FACS for phase distribution of the cell cycle. **d**–**i** Inhibition of proteasome induced mitotic arrest, multi-polar spindle formation and ballooning bubbles from cell membranes. SNU449 subline that stably expressed histone H2B-EGFP and mCherry-α-tubulin were treated with vehicle or 30 nM BTZ (**d**, **f**, **h**), or transfected with NC or siPSMC5-1/2 (mixture of siPSMC5-1 and siPSMC5-2) for 24 h (**e**, **g**, **i**), followed by live-cell imaging for 46 h (**d**, **f**, **h**) or 70 h (**e**, **g**, **i**). For **d** (Vehicle, *n* = 25; BTZ, *n* = 14) and **e** (NC, *n* = 28; siPSMC5-1/2, *n* = 27), the time from nuclear envelope breakdown (NEBD) to the end of anaphase or cell death was designated as mitotic duration (*right* panel). White arrows indicate the large bubbles blowing from the plasma membrane. Scale bar, 5 μm. For **f** and **g**, cell death was determined by the emergence of pyroptosis characteristics or cell detachment, and the fractions of cells died at interphase or M-phase were quantified based on at least 118 cells in each group. For **h** (Vehicle, *n* = 25; BTZ, *n* = 14) and **i** (NC, *n* = 28; siPSMC5-1/2, *n* = 27), the fates of individual mitotic cell are shown. For **d**, **e**, **h** and **i**, the time point of NEBD was set as 0. **j**, **k** Inhibition of proteasome caused multi-polar spindle formation. SNU449 cells were treated with vehicle or 15 nM BTZ for 30 h (**j**), or transfected with the indicated RNA duplexes for 60 h (**k**), then stained for pericentrin (PCNT, red), α-tubulin (TUBA, green) and DAPI (blue) to indicate centrosome, spindle and chromosome, respectively. The proportion of mitotic cells possessing multi-polar spindles was calculated (*right* panel). Scale bar, 2.5 μm. Error bars: SEM from at least three independent experiments. One-way ANOVA (**a**–**c** and **k**) and Student’s *t* test (**d**, **e** and **j**) were used. *P < 0.05; **P < 0.01; ***P < 0.001; ****P < 0.0001; ns not significant

We first assessed whether inhibition of proteasome affected the fates of cells in M-phase. Staining of Ser-10-phosphorylated histone H3 (pH3-S10) was used to mark M-phase cells. Flow cytometry analysis showed that treatment with BTZ significantly increased the ratio of cells at M-phase (Fig. 1a; Supplementary Fig. 1a, b). And BTZ treatment also enhanced the proportion of G2-phase cells in a dose-dependent manner, and high dose of BTZ showed a reduced capability to increase the fraction of M-phase cells due to significant G2-phase arrest (Fig. 1a; Supplementary Fig. 1a, b). These findings were validated with the treatment of carfilzomib (CFZ) (Supplementary Fig. 1c), the second-generation proteasome inhibitor,^25^ which irreversibly binds to 20S-CR. We then validated the effects of proteasome inhibition by silencing the individual AAA+ ATPase or representative non-ATPase subunits of proteasomal 19S regulatory particle (Supplementary Fig. 1d, e). Knockdown of PSMC5, but not other PSMCs, mimicked the effects of BTZ and CFZ in increasing both G2- and M-phase populations in all three cell lines examined (Fig. 1b; Supplementary Fig. 1f, j). However, silencing non-ATPase subunits of 19S regulatory particle, like Rpn10, Rpn13 (ubiquitin receptors) or Rpn11 (de-ubiquitinating enzyme),^18^ failed to raise the population of G2-phase cells (Fig. 1c), implying that inhibiting the non-ATPase subunits of 19S-RP may not mimic the effects of BTZ.

The fates of mitotic cells after proteasome inhibition were then examined by the live-cell imaging under a time-lapse microscopy, using SNU449 subline stably expressing histone H2B-EGFP and mCherry-α-tubulin fusion proteins, which indicated chromosomes and microtubules, respectively. Upon treatment with BTZ or CFZ or silencing of PSMC5, a great majority of mitotic cells showed a significant extension of mitotic duration (mean time of vehicle vs. BTZ: 46 vs. 1857 min; vehicle vs. CFZ: 43 vs. 515 min; NC vs. siPSMC5: 40 vs. 530 min) (Fig. 1d; Supplementary Fig. 2a; Fig. 1e). Compared to control group, inhibition of proteasome resulted in a significantly increased cell death at M-phase (vehicle vs. BTZ: 0% vs. 8.8%; vehicle vs. CFZ: 0% vs. 12.1%; NC vs. siPSMC5-1/2: 0% vs. 14.4%) (Fig. 1f; Supplementary Fig. 2b; Fig. 1g), while a very low cell population died at interphase in both control group and proteasome-inhibiting group (vehicle vs. BTZ: 2.54% vs. 2.04%; vehicle vs. CFZ: 2.2% vs. 2.4%; NC vs. siPSMC5-1/2: 1.60% vs. 2.06%) (Fig. 1f; Supplementary Fig. 2b; Fig. 1g), indicating that most of the BTZ-, CFZ- or siPSMC5-treated cells which underwent mitotic arrest eventually died without exit from M-phase. We also observed that during mitotic arrest, multi-polar spindles appeared after spindle bipolarization (Fig. 1h; Supplementary Fig. 2c; Fig. 1i), and further immunofluorescent staining for microtubules and centrosomes confirmed that a large proportion of mitotic cells in BTZ- or siPSMC5-treated group displayed multi-polar spindles (Fig. 1j, k; Supplementary Fig. 3a–d). These results suggest that inhibition of proteasome may promote mitotic catastrophe by inducing aberrant spindle assembly and mitotic failure.

### Inhibition of proteasome induces pyroptosis in M-phase via cGAS-caspase-3-GSDME cascade

**Fig. 2:**
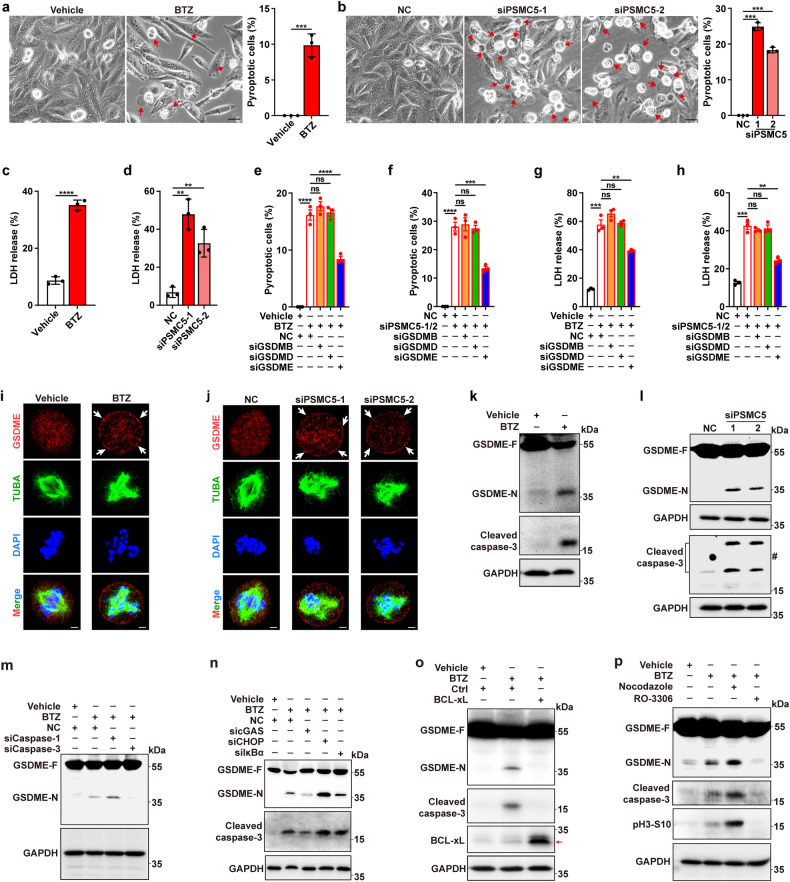
Inhibition of proteasome induces mitotic pyroptosis via GSDME. **a**, **b** Proteasome inhibition induced morphology of pyroptosis. Five random fields in each well were captured and then subjected to analysis for the rate of cells with pyroptosis morphology. One of the five fields is shown as representative image for each group. Red arrows indicate the pyroptotic cells with large ballooning bubbles. The proportion of pyroptotic cells was calculated (*right* panel). Scale bar, 20 μm. **c**, **d** Proteasome inhibition stimulated LDH release. **e**, **f** GSDME silencing attenuated the proteasome inhibition-induced increase of pyroptotic cells. **g**, **h** GSDME knockdown abrogated proteasome inhibition-induced LDH release. For **e**–**h**, SNU449 cells were transfected with NC or siRNA targeting the indicated gasdermins (siGSDMs) for 24 h, then treated with 15 nM BTZ for another 48 h (**e**, **g**), or cells were co-transfected with siGSDMs and siPSMC5-1/2 for 72 h (**f**, **h**) before phase-contrast imaging (**e**, **f**) or LDH release assay (**g**, **h**). **i**, **j** Proteasome inhibition induced translocation of GSDME to the plasma membrane of multi-polar mitotic cell. White arrows indicate the clusterization of GSDME on cell membrane. Scale bar, 2.5 μm. **k**, **l** Proteasome inhibition induced the cleavage of caspase-3 and GSDME. SNU449 cells were treated with 15 nM BTZ for 48 h (**a**, **c**, **i**, **k**), or transfected with the indicated RNA duplexes for 72 h (**b**, **d**, **j**, **l**) before phase-contrast imaging (**a**, **b**), LDH detection (**c**, **d**), immunofluorescent staining for GSDME (Red), α-tubulin (TUBA, green) and chromosomes (DAPI, blue) (**i**, **j**), or immunoblotting (**k**, **l**). #, unspecific band. m Silencing caspase-3 but not caspase-1 blocked the BTZ-induced GSDME cleavage. **n** Silencing cGAS but not CHOP or IκBα attenuated the BTZ-induced cleavage of caspase-3 and GSDME. For **m**, **n**, SNU449 cells were transfected with NC or the indicated siRNA for 24 h, then treated with 15 nM BTZ for another 48 h before immunoblotting. **o** Ectopic expression of BCL-xL attenuated the BTZ-induced cleavage of caspase-3 and GSDME. SNU449-BCL-xL and its control line SNU449-Ctrl were treated with vehicle or 15 nM BTZ for 48 h before immunoblotting. Red arrows indicate the target band. **p** BTZ-induced cleavage of GSDME was enhanced by nocodazole but was inhibited by CDK1 inhibitor RO-3306. SNU449 cells were pretreated with vehicle, 50 ng/mL nocodazole or 10 μM RO-3306 for 6 h, followed by treatment with vehicle or 15 nM BTZ for another 48 h before immunoblotting. Error bars: SEM from at least three independent experiments. Student’s *t* test (**a** and **c**) and one-way ANOVA (**b** and **d**–**h**) were used. **P < 0.01; ***P < 0.001; ****P < 0.0001; ns not significant

Notably, live-cell imaging assays disclosed that the mitotic cells in BTZ/CFZ/siPSMC5 groups exhibited morphology features of pyroptosis, that is, cells swell and form balloon-like membrane structure (Fig. 1d; Supplementary Fig. 2a; Fig. 1e), which we termed mitotic pyroptosis. We therefore verified the effects of BTZ/siPSMC5 on the mitotic pyroptosis of different tumor cell lines, based on characteristic morphology, cleavage of gasdermin, and release of LDH. Compared to control group, BTZ treatment or PSMC5 silencing significantly increased the proportion of cells with pyroptosis morphology (Fig. 2a, b; Supplementary Fig. 4a, b) and induced the release of LDH (Fig. 2c, d; Supplementary Fig. 4c, d), with very few cells undergoing apoptosis (Supplementary Fig. 4e, f).

We next explored which member of gasdermin family mediated the proteasome inhibition-induced mitotic pyroptosis. Examination on the levels of five gasdermin members revealed high level of GSDME in all three cell lines used in this study, and detectable levels of GSDMB and GSDMD in two of them (Supplementary Fig. 5a). Silencing of GSDME, but not GSDMB and GSDMD (Supplementary Fig. 5b), attenuated the roles of BTZ and siPSMC5 in increasing the fraction of cells with pyroptosis features (Fig. 2e, f) and in promoting LDH release (Fig. 2g, h; Supplementary Fig. 5c), suggesting GSDME may mediate BTZ/siPSMC5-induced pyroptosis. Consistently, accumulation of GSDME foci on plasma membrane was observed in the BTZ- or siPSMC5-induced multi-polar mitotic cells (Fig. 2i, j). And immunoblotting assays confirmed that both BTZ and siPSMC5 promoted the cleavage of GSDME (Fig. 2k, l; Supplementary Fig. 5d, e).

We further explored the mechanisms for BTZ-induced mitotic pyroptosis, especially how BTZ regulates GSDME cleavage. It has been reported that GSDME is cleaved by caspase-3,^12^ while GSDMD is cleaved by caspase-1.^40^ We found that BTZ- or siPSMC5-treatment increased the level of active caspase-3 (Fig. 2k, l; Supplementary Fig. 5d, e), and the depletion of caspase-3, but not caspase-1 (Supplementary Fig. 5f), blocked BTZ-induced GSDME cleavage (Fig. 2m), suggesting that caspase-3-GSDME cascade, but not caspase-1-GSDMD, mediates BTZ/siPSMC5-induced pyroptosis. It is shown that inhibition of proteasome can activate caspase-3 by upregulating IκBα or inducing ER-stress,^25,41^ and mitotic arrest can activate caspase-3 through cGAS signaling, that is, cGAS-activated STING induces IRF3 phosphorylation, leading to inhibition of BCL-xL, and in turn permeabilization of mitochondrial outer membrane and consequent caspase-3 activation.^7^ Therefore, we examined whether these pathways were involved in proteasome inhibitor-induced pyroptosis. The results showed that BTZ-induced caspase-3 activation and GSDME cleavage were attenuated by silencing cGAS (Supplementary Fig. 5g; Fig. 2n) or overexpression of BCL-xL (Fig. 2o). However, silencing of CHOP to inhibit ER stress signaling, or inhibition of IκBα to enhance NF-κB activity were unable to abrogate BTZ-induced caspase-3 and GSDME cleavage (Fig. 2n). Moreover, BTZ-induced caspase-3/GSDME activation was strengthened by nocodazole that triggered mitotic arrest, but it was inhibited by CDK1 inhibitor (RO-3306) that blocked mitotic entry (Fig. 2p). These results suggest that proteasome inhibitor-induced mitotic arrest may activate cGAS signaling, which inactivate BCL-xL, resulting in the activation of caspase-3-GSDME cascade and subsequent mitotic catastrophe in a form of mitotic pyroptosis.

### Combined inhibition of proteasome and WEE family kinases displays synergistic effect in inducing mitotic pyroptosis and selectively killing cancer cells

**Fig. 3:**
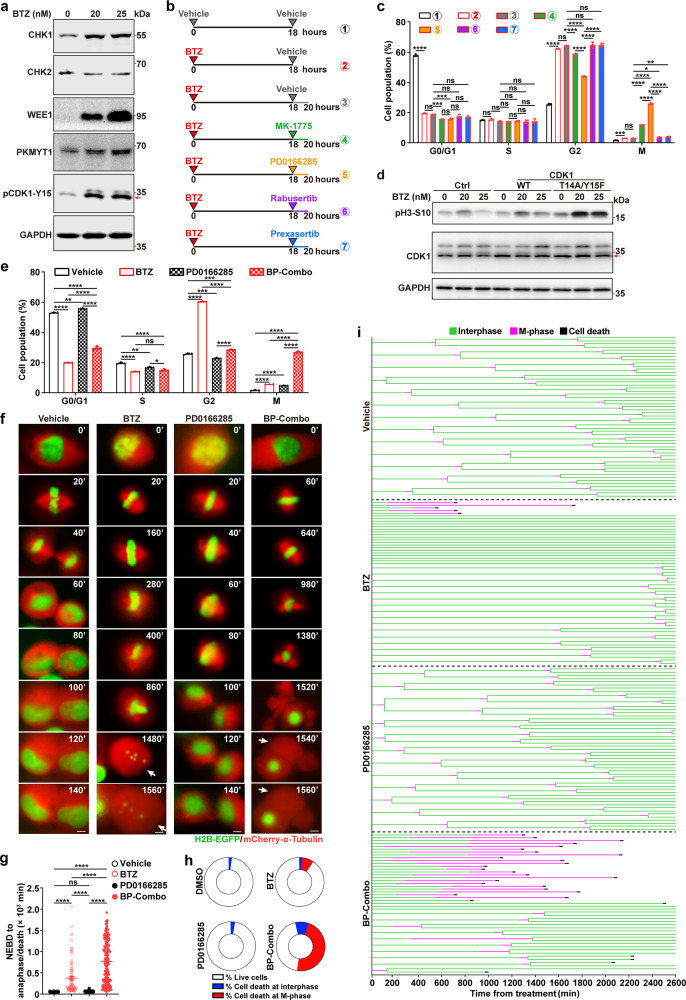
PD0166285 abrogates BTZ-induced G2-phase arrest and enhances BTZ-induced mitotic catastrophe. **a** BTZ increased the protein levels of CHK1, WEE1, PKMYT1 and Tyr15-phosphorylated CDK1. SNU449 cells were treated with BTZ at the indicated concentrations for 18 h before immunoblotting. **b**, **c** PD0166285 effectively alleviated BTZ-induced G2-phase arrest. Schematic diagrams of study design are shown in **b**. The triangles indicate the time points for the indicated treatment. SNU449 cells were pretreated with vehicle or 20 nM BTZ for 18 h, followed by treatment with vehicle or 0.5 μM of the indicated inhibitors for another 2 h before pH3-S10/PI staining and FACS (**c**). **d** Ectopic expression of the dominant active mutant CDK1-T14A/Y15F enhanced the BTZ-induced up-regulation of pH3-S10. SNU449-CDK1, SNU449-CDK1-T14A/Y15F and control line SNU449-Ctrl were treated with BTZ at the indicated concentrations for 30 h before immunoblotting. **e** Concurrent exposure to PD0166285 potentiated BTZ-induced accumulation of mitotic cells. SNU449 cells were treated with vehicle, 20 nM BTZ, 0.25 μM PD0166285, or BP-Combo (20 nM BTZ and 0.25 μM PD0166285) for 24 h before pH3-S10/PI staining and FACS. **f**–**i** PD0166285 amplified the effects of BTZ in inducing mitotic arrest and mitotic cell death. SNU449 subline that stably expressed histone H2B-EGFP and mCherry-α-tubulin were treated with 20 nM BTZ, 0.25 μM PD0166285, or BP-Combo, followed by live-cell imaging for a total of 2600 min. Representative images (**f**) and quantification of mitotic duration (**g**) are shown. White arrows indicate the large bubbles blowing from the plasma membrane (**f**). Scale bar, 5 μm. The cell death was determined by the emergence of pyroptosis characteristics or cell detachment, and the fractions of cell death at interphase or M-phase were quantified based on at least 146 cells in each group (**h**). In **i**, the fates of each cell within 2600 min are presented, each horizontal line represents one cell, and a fork in the line indicates cell division and cell fate of each daughter cell is also shown. The beginning time of BTZ treatment was set as 0. For **a** and **d**, red arrows indicate the target band. Error bars: SEM from at least three independent experiments. One-way ANOVA (**c**, **e** and **g**) was used. *P < 0.05; **P < 0.01; ***P < 0.001; ****P < 0.0001; ns not significant

We found that BTZ treatment arrested a large number of cells at G2-phase (Fig. 1a; Supplementary Fig. 1a, b). Therefore, we explored whether abrogation of G2/M checkpoint could facilitate the mitotic entry and subsequent mitotic catastrophe of BTZ-treated tumor cells. The levels of key regulators of G2/M checkpoint,^42,43^ including checkpoint kinase 1/2 (CHK1/2) and WEE family kinases (WEE1, PKMYT1) and their downstream effector (Y15-phosphorylated CDK1), were first analyzed. As shown, CHK1, WEE1, PKMYT1, but not CHK2, were upregulated upon BTZ treatment (Fig. 3a; Supplementary Fig. 6a). Consistently, WEE1/PKMYT1-induced phosphorylation at the Tyr15 site of CDK1 (pCDK1-Y15), which inactivated CDK1, was enhanced in BTZ-treated cells (Fig. 3a; Supplementary Fig. 6a). Next, cells were treated with BTZ first, then exposed to an inhibitor that repressed WEE1 alone (MK-1775) or inhibited both WEE1 and PKMYT1 (PD0166285), or exposed to an inhibitor that suppressed CHK1 alone (rabusertib) or inhibited both CHK1 and CHK2 (prexasertib) (Fig. 3b). The results showed that compared with BTZ monotreatment, sequential treatment with BTZ and MK-1775 or PD0166285 significantly reduced G2-phase population and increased M-phase cells, and PD0166285 showed a much stronger effect than MK-1775 (Fig. 3c; Supplementary Fig. 6b). Furthermore, overexpression of CDK1-T14A/Y15F, the dominant active mutant CDK1 that is resistant to WEE family kinase-induced inhibitory phosphorylation, increased the levels of pH3-S10 in BTZ-treated cells (Fig. 3d), which mimicked the effects of WEE family kinase inhibitors to promote mitotic entry. Notably, treatment with rabusertib or prexasertib could not alleviate the BTZ-induced G2-phase arrest (Fig. 3b, c; Supplementary Fig. 6b). Consistently, inhibition of ATR, the activator of CHK1/CHK2, by AZD6738 failed to relieve G2-phase arrest and promote mitotic entry upon BTZ treatment (Supplementary Fig. 6c, d).

We then analyzed the expression level of representative proteasome subunits and WEE family kinases in patients of 17 cancer types from The Cancer Genome Atlas (TCGA).^44^ As shown, the mean expression level of a subset of proteasome subunit genes, including 20S core subunits (α1–7, β1–7) and 19S regulatory subunits (PSMC1–6), was significantly upregulated in various human malignancies (15/17, Supplementary Fig. 6e), compared with their normal counterparts. Interestingly, WEE1 was upregulated in only 3/17 cancer types, whereas PKMYT1 elevated in 16/17 cancer types (Supplementary Fig. 6e). These results suggest that the combined treatment of proteasome and WEE family inhibitors may be effective for a wide range of cancer types. And PD0166285, which can inhibit both PKMYT1 and WEE1, may be more powerful than MK-1775 in the combination treatment. Therefore, we chose the treatment of concurrent BTZ and PD0166285 exposure (designated as BP-Combo) for further study. As shown, the BP-Combo treatment had the same effect (Fig. 3e; Supplementary Fig. 6f) as sequential BTZ and PD0166285 treatment on the mitosis (Fig. 3c; Supplementary Fig. 6b). Consistently, combined PD0166285 treatment diminished BTZ-enhanced phosphorylation of CDK1-Y15 (Supplementary Fig. 6g). However, PD0166285 didn’t affect BTZ-induced accumulation of WEE1 (Supplementary Fig. 6h), indicating that PD0166285 may abrogate BTZ-induced G2-phase arrest via blocking the WEE1/PKMYT1-induced CDK1-T14/Y15 phosphorylation, without affecting proteasome activity.

The fates of cells treated with BP-Combo were further confirmed by live-cell imaging assay. Compared to control group, treatment with BTZ alone or BP-Combo, but not PD0166285 alone, induced significant M-phase arrest, multi-polar spindles and subsequent mitotic pyroptosis (Fig. 3f). The mean time of mitotic duration in the cells treated with vehicle, BTZ, PD0166285, or BP-Combo was 50, 377, 64, or 769 min, respectively (Fig. 3g). And longer mitotic duration was correlated with higher mitotic cell death (vehicle, BTZ, PD0166285, BP-Combo: 0%, 7.8%, 0%, 47.3%) (Fig. 3h, i). Notably, mitotic arrest and spindle multi-polarization appeared no earlier than three and four hours, respectively, after addition of BTZ (Fig. 3i, data not shown). The significant interphase extension upon BTZ treatment was diminished in BP-Combo group (Fig. 3i), which was reminiscent of the above findings that BP-Combo could overcome the BTZ-induced G2-phase arrest (Fig. 3c, e; Supplementary Fig. 6b, f). These data suggest that PD0166285 may augment the BTZ-induced mitotic catastrophe by releasing the cells from G2-phase arrest and promoting mitotic entry.

**Fig. 4:**
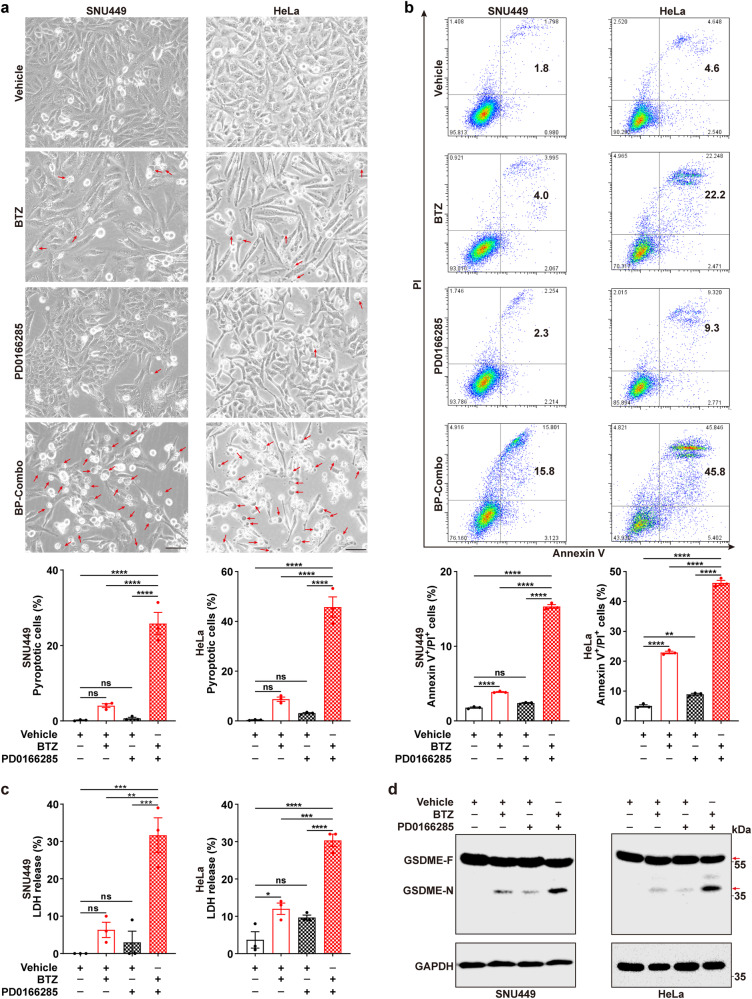
PD0166285 augments BTZ-induced pyroptosis. **a** PD0166285 enhanced the role of BTZ in increasing the proportion of cells with pyroptosis morphology. Red arrows indicate the pyroptosis cells with large bubbles. Five random fields in each well were captured and then subjected to analysis for the rate of cells with pyroptosis morphology. One of the five fields is shown as representative image for each group. Scale bar, 50 μm. **b** PD0166285 enhanced the role of BTZ in increasing the fraction of cells with Annexin V/PI double-staining. **c** PD0166285 promoted the effects of BTZ in promoting LDH release. **d** PD0166285 enhanced the role of BTZ in promoting GSDME cleavage. Red arrows indicate the target band. For **a**–**d**, SNU449 (*left* panel) and HeLa (*right* panel) cells were exposed to vehicle, 20 nM BTZ, 0.25 μM PD0166285, or BP-Combo for 30 h (SNU449) or 24 h (HeLa) before phase-contrast imaging (**a**), Annexin V/PI staining (**b**), LDH release assay (**c**) and immunoblotting (**d**). Error bars: SEM from at least three independent experiments. One-way ANOVA (**a**–**c**) was used. *P < 0.05; **P < 0.01; ***P < 0.001; ****P < 0.0001; ns not significant

Next, we investigated whether PD0166285 enhanced the BTZ-induced mitotic pyroptosis. Compared with BTZ or PD0166285 monotreatment, BP-Combo displayed much stronger effects in increasing the fraction of cells with pyroptosis morphology (Fig. 4a; Supplementary Fig. 7a) or with Annexin V/PI double-staining (Fig. 4b; Supplementary Fig. 7b), and BP-Combo also had greater ability in enhancing LDH release (Fig. 4c; Supplementary Fig. 7c) and cytotoxic GSDME cleavage (Fig. 4d; Supplementary Fig. 7d). In contrast, very few apoptotic cells were observed in BTZ, PD0166285 and BP-Combo group (Supplementary Fig. 7e), indicating that pyroptosis but not apoptosis mainly contributed to the synergistic lethality effect. The synergistic effect was also verified using the combination of CFZ and PD0166285 (Supplementary Fig. 7f). Nevertheless, AZD6738, an ATR inhibitor, was unable to promote BTZ-induced pyroptosis (Supplementary Fig. 7g), in accord with its failure in promoting mitotic entry of BTZ-treated cells. These findings indicate that the combined treatment of BTZ with PD0166285 may have a synergistic effect in inducing mitotic catastrophe by promoting mitotic entry and pyroptosis.

**Fig. 5:**
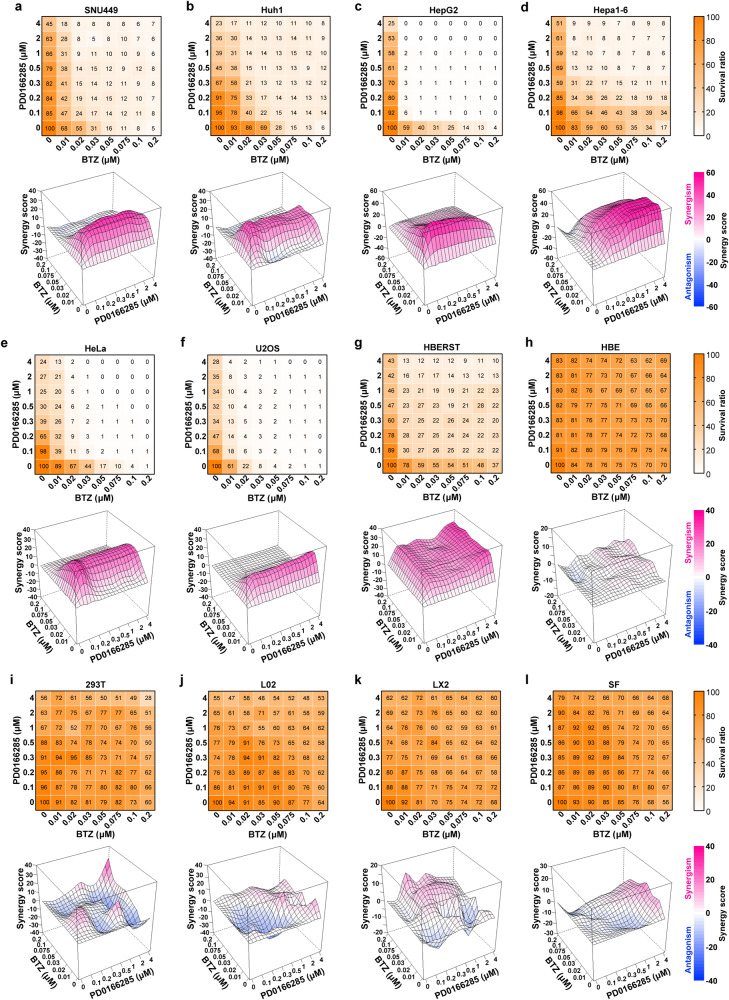
BTZ and PD0166285 shows synergistic effect in killing cancer cells but not immortalized cells. **a**–**g** Various cancer cell lines were sensitive to BP-Combo treatment. **h**–**l** Immortalized cell lines and normal cells were resistant to BP-Combo treatment. Cancer cell lines from hepatoma (SNU449, Huh1, HepG2, Hepa1-6), cervical cancer (HeLa) and osteosarcoma (U2OS), transformed human bronchial epithelial cell line (HBERST), immortalized cell lines (HBE, 293T, L02, LX2) and normal cell (SF) were treated for 48 h with a combination of BTZ and PD0166285 at the indicated concentration. Cell survival was measured by Alamar Blue assay. Cooperativity screens (*upper* panels) and Loewe plots (*down* panels) for the synergistic effect of BTZ and PD0166285 are shown based on at least three independent experiments. In *upper* panels, color bars indicate the percentage of surviving cells in BP-Combo-treated group, which was normalized to untreated group. In *down* panels, color bars indicate synergy score in the Lowe plots; a score greater than 0 indicates synergism, and less than 0 indicates antagonism

We then used Bliss combination index (Bliss CI, value below 1 indicates synergy) to evaluate the combinatorial effect of BTZ and PD0166285 in killing cancer cells at different concentrations. Significant synergistic lethality between BTZ and PD0166285 was observed in different solid tumor cells originated from various tissue types, including the liver (SNU449, Huh1, HepG2, SK-Hep-1 and Hepa1-6), cervix (HeLa) and bone (U2OS), and also in transformed human bronchial epithelial cell (HBERST), as evidenced by Bliss CI value below 1 (0.21–0.74, Supplementary Table 1). Importantly, much higher IC50 and no cooperative lethality between BTZ and PD0166285 were detected in immortalized cell lines (HBE, 293T, L02, LX2) and normal SF (Supplementary Table 1). The Loewe plots also revealed synergistic effects of BTZ and PD0166285 in killing cancer/transformed cells rather than immortalized/normal cells (Fig. 5 and Supplementary Fig. 8). Furthermore, the IC50 of BTZ and PD0166285 in the combined treatment was much lower than that in BTZ or PD0166285 monotreatment across tested cancer/transformed cell lines (Supplementary Table 1).

Collectively, BP-Combo treatment may be effective for a wide range of solid tumor types and have a potential to specifically eliminate cancer cells, at least partly via mitotic pyroptosis.

### Combined inhibition of proteasome and WEE family kinases significantly represses tumor growth and metastasis in vivo

**Fig. 6:**
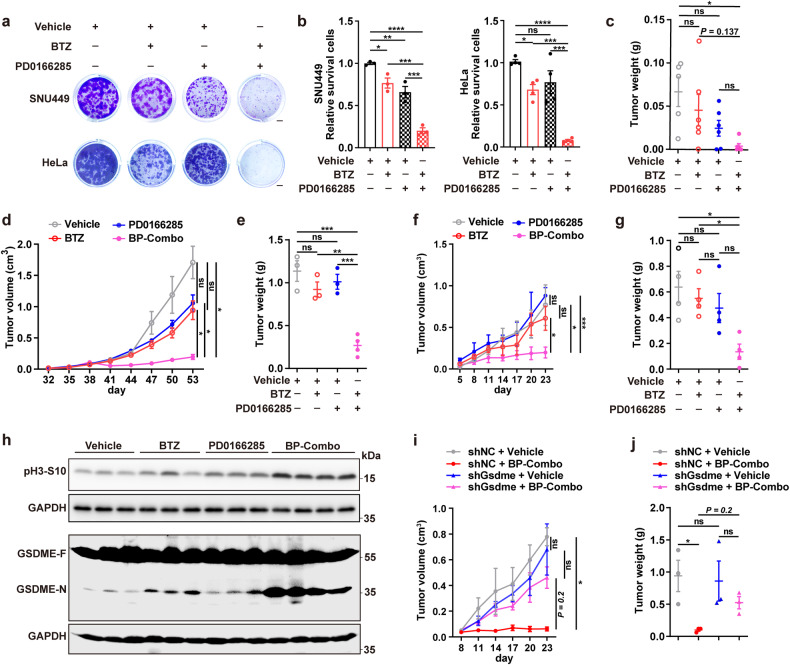
BTZ and PD0166285 has a synergistic effect in suppressing growth of subcutaneous tumor xenografts. **a**, **b** BP-Combo showed much stronger effect than BTZ or PD0166285 monotreatment in repressing colony formation of tumor cells. SNU449 and HeLa cells were treated with vehicle, BTZ or PD0166285 alone, or with BP-Combo for 10 days before staining with 0.1% crystal violet. The representative images (**a**) and colony quantification (**b**) are shown. Scale bar, 2 mm. Error bars: SEM from at least three independent experiments. **c**–**g** BP-Combo showed much stronger effect than BTZ or PD0166285 monotreatment in inhibiting subcutaneous tumor xenograft development. The BALB/c nude mice were subcutaneously injected with HeLa cells, and then intraperitoneally injected with the indicated inhibitors one day after tumor cell implantation (early treatment, **c**), or when tumor volumes reached ~50 mm^3^ (late treatment, **d**, **e**). Early treatment: *n* = 5 (vehicle), 6 (BTZ), 6 (PD0166285) and 6 (BP-Combo). Late treatment: *n* = 3 (vehicle), 3 (BTZ), 3 (PD0166285) and 4 (BP-Combo). For **f**, **g**, C57BL/6J mice were subcutaneously injected with Hepa1-6 cells, and then intraperitoneally injected with the indicated inhibitors when tumor volumes reached ~50 mm^3^. *n* = 4 for each group. **h** BP-Combo had much stronger effect than BTZ or PD0166285 monotreatment in increasing pH3-S10 level and inducing GSDME cleavage in tumor xenografts. Mouse xenograft tissues from late treatment groups in Figures **d** and **e** were analyzed. **i**, **j** Silencing of GSDME diminished the anti-tumor effect of BP-Combo. C57BL/6J mice were subcutaneously injected with Hepa1-6-shNC or Hepa1-6-shGsdme cells, and then intraperitoneally administered with vehicle or BP-Combo treatment when tumor volumes reached ~50 mm^3^. *n* = 3 for each group. One-way ANOVA (**b**, **c**, **e**, **g**) and two-way ANOVA (**d**, **f**, **i**, **j**) were used. *P < 0.05; **P < 0.01; ***P < 0.001; ****P < 0.0001; ns not significant

Next, we explored the effect of BP-Combo on tumor development. The in vitro studies revealed that compared with BTZ or PD0166285 monotreatment, BP-Combo showed a much stronger inhibition on colony formation of tumor cells (Fig. 6a, b; Supplementary Fig. 9a, b). The in vivo studies were conducted by administering BTZ or/and PD0166285 at the early or late stage of subcutaneous xenograft development in immunodeficient mice. We found that early treatment with BP-Combo, from day one after cancer cell inoculation, dramatically inhibited the formation of HeLa xenografts, whereas BTZ or PD0166285 monotreatment only showed modest effect (Fig. 6c; Supplementary Fig. 9c). And late treatment with BP-Combo, beginning when tumor volumes reached appropriately 50 mm^3^, also exerted a more prominent inhibitory role on xenograft growth of both HeLa (Fig. 6d, e; Supplementary Fig. 9d) and Hepa1-6 (Fig. 6f, g; Supplementary Fig. 9e) cancer cells, compared with BTZ or PD0166285 monotreatment. We found that combined treatment with PD0166285 did not increase the concentration of BTZ in xenograft tissue (Supplementary Fig. 9f). Notably, compared with BTZ or PD0166285 group, xenografts from BP-Combo group showed a much higher level of mitotic marker pH3-S10 and cleaved GSDME (Fig. 6h), suggesting the enhanced mitotic arrest and pyroptosis. To verify the in vivo function of GSDME in BP-Combo treatment, Hepa1-6 cells which stably expressing shNC or shGsdme (Supplementary Fig. 9g) were injected subcutaneously into immunocompetent mice. As shown, the role of BP-Combo treatment in repressing xenograft growth was attenuated after GSDME in Hepa1-6 was knocked down (Fig. 6i, j and Supplementary Fig. 9h). These findings imply that the anti-tumor effect of BP-Combo depends on GSDME-mediated pyroptosis in cancer cells.

**Fig. 7:**
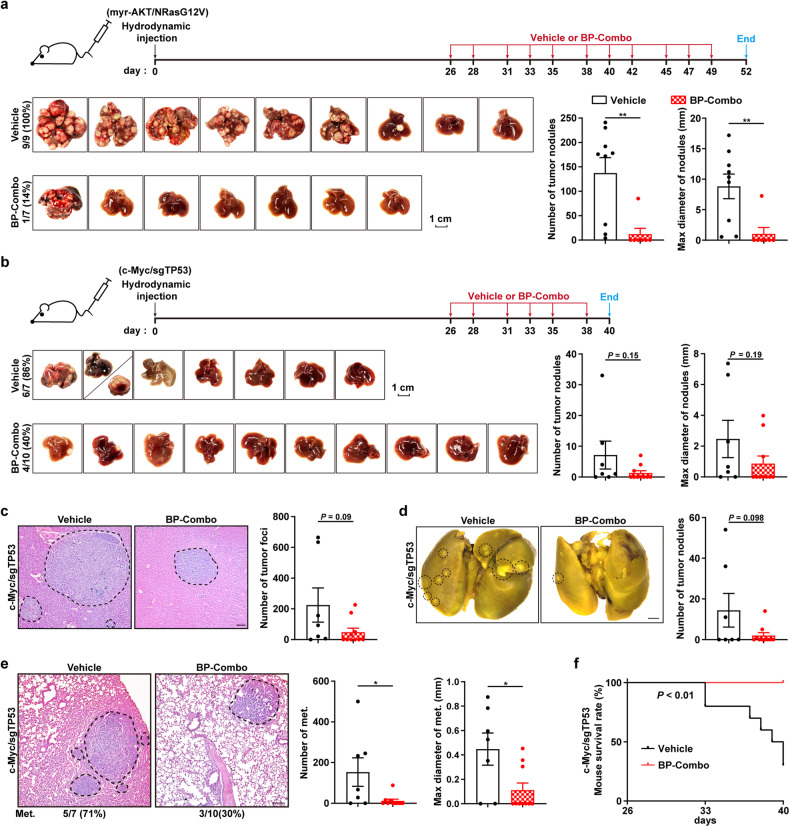
BP-Combo inhibits the growth and metastasis of mouse autochthonous liver tumors. **a**–**c** BP-Combo suppressed the growth of mouse autochthonous liver tumors. The day when C57BL/6J mice were hydrodynamically injected with the indicated plasmids was set as day 0 (*upper* panels). The tumor incidences and photographs of the livers (*left* panels), the number of macroscopic tumor nodules in the livers (*middle* panel) and the maximal diameter of macroscopic tumor nodules (*right* panels) are shown in **a** and **b**. The representative images of hematoxylin-eosin (H&E) staining and the numbers of microscopic tumor foci in the livers of c-myc/sgTP53-injected mice are shown in **c**. Scale bars, 1 cm (**a**, **b**) and 100 μm (**c**). **d**, **e** BP-Combo inhibited pulmonary metastasis of c-Myc/sgTP53-induced liver tumors. Photographs of the lungs (*left* panel) and the number of macroscopic metastatic nodules (*right* panel) are shown in **d**. H&E staining (*left* panel), the number (*middle* panel) and maximal diameter (*right* panel) of microscopic pulmonary metastatic foci are shown in **e**. Metastasis rates are indicated under the images (**e**). Scale bars, 1 mm (**d**) and 100 μm (**e**). Met. metastases. **f** BP-Combo improved the survival of mice with c-Myc/sgTP53-induced liver tumors. Student’s *t* test (**a**–**e**) and the log-rank test (**f**) was used. *P < 0.05; **P < 0.01

We also assessed the therapeutic efficacy of dual proteasome and WEE kinase inhibition on the development of autochthonous liver cancers in immunocompetent mice, which were hydrodynamically injected with plasmids expressing myr-AKT and NRasG12V or c-Myc and sgTP53. Drug treatment was performed at 26 days after hydrodynamic injection of plasmids. Compared with vehicle-treated group, BP-Combo significantly reduced the incidences of both myr-AKT/NRasG12V- (vehicle vs. BP-combo: 9/9 vs. 1/7) and c-Myc/sgTP53-induced liver tumors (vehicle vs. BP-combo: 6/7 vs. 4/10), and also decreased the number and size of tumor nodules (Fig. 7a–c), suggesting an inhibitory role of BP-Combo on liver cancer formation and growth. For c-Myc/sgTP53 injection model, in comparison with vehicle control group, BP-Combo decreased the incidence of pulmonary metastasis (vehicle vs. BP-combo: 5/7 vs. 3/10) as well as the number and size of metastatic foci (Fig. 7d, e), and greatly prolonged mouse survival (Fig. 7f). Both picric acid staining and histology analysis revealed no pulmonary metastasis (Supplementary Fig. 10a, b) and no mice died at the end of experiment in AKT/NRasG12V injection model.

Collectively, BP-Combo may represent an effective regimen in repressing in vivo tumor development.

### Combined inhibition of proteasome and WEE family kinases has no obvious side effects in the treated mice

**Fig. 8:**
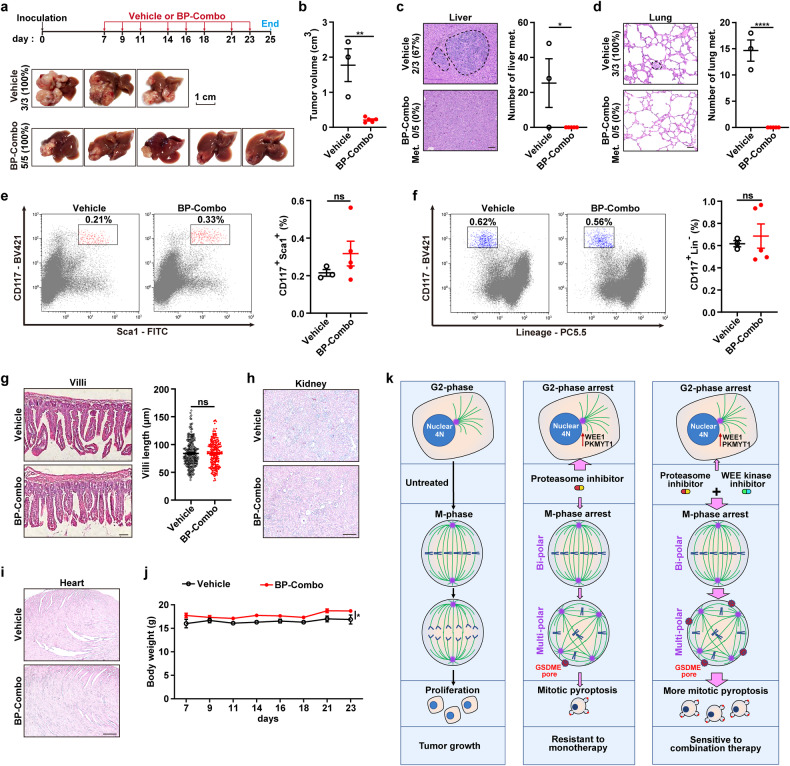
BP-Combo represses the growth and metastasis of liver orthotopic xenografts and has no obvious toxicity on normal tissues. **a**, **b** BP-Combo suppressed the growth of liver orthotopic xenografts. Hepa1-6 cells were inoculated under the capsule of the left hepatic lobe of C57BL/6 mice. Vehicle or BP-Combo treatment was intraperitoneally administered at the indicated time. The tumor incidences and photographs of dissected livers (**a**) and the tumor volume (**b**) are shown. **c**, **d** BP-Combo suppressed metastasis of liver xenografts. The representative images of H&E staining and metastasis rates (*left* panel) and the number of the intrahepatic (**c**) or pulmonary (**d**) metastatic foci (*right* panel) are shown. Scale bar, 50 μm (**c**) and 25 μm (**d**). Met. metastases. **e**, **f** The proportion of hematopoietic stem and progenitor cells were not affected in BP-Combo-treated mice. Bone marrow cells were isolated from the vehicle or BP-Combo-treated mice and analyzed by flow cytometry to detect CD117^+^Sca1^+^ population (**e**) and CD117^+^Lin^–^ population (**f**). **g** BP-Combo did not change the lengths of ileum villi. H&E staining of the ileum villi are shown (*left* panel) and the villi lengths were calculated (*right* panel). Scale bar, 25 μm. BP-Combo did not affect the size and tissue structure of kidney (**h**) and heart (**i**). Scale bar, 50 μm (**h**, **i**). **j** BP-Combo did not influence mouse body weight. For **b**–**j**, samples/mice from **a** were subjected to the indicated analyses. **k** The working model of synergistic interaction between proteasome and WEE kinase inhibitors. Student’s *t* test (**b**–**g**) and two-way ANOVA (**j**) were used. *P < 0.05; **P < 0.01; ****P < 0.0001; ns not significant

We further validated the role of BP-Combo in liver orthotopic xenograft model and evaluated its potential toxicities in immunocompetent mice. Consistent with the above findings, BP-Combo significantly inhibited the growth (Fig. 8a, b) and metastasis (Fig. 8c, d) of Hepa1-6 xenografts.

We then examined the toxicity of BP-Combo in normal tissues that have rapidly proliferating cells and are susceptible to chemotherapy. Inhibition of bone marrow is one of the most common dose-limiting side effects for cancer therapy. Compared with control mice, BP-Combo-treated mice did not show significant changes in the subpopulation percentage of CD117^+^Sca1^+^ (hematopoietic stem and multipotent progenitor cells) (Fig. 8e) or CD117^+^Lin^−^ (hematopoietic precursors) (Fig. 8f) cells in the bone marrow. Neither the villi length of the small intestine (Fig. 8g) nor the size and tissue structure of the kidney (Fig. 8h) and heart (Fig. 8i) were affected by BP-Combo treatment. And none of the BP-Combo-treated mice showed reduction in body weight (Fig. 8j) or changes in food intake and fecal character (data not shown). These data suggest that BP-Combo may significantly suppress the growth and metastasis of tumors at the safe treatment dose and duration.

Collectively, this study suggest that inhibition of proteasome causes mitotic arrest and subsequent multi-polar spindle formation of tumor cells, resulting in mitotic catastrophe via caspase-3/GSDME-dependent pyroptosis; proteasome inhibitor also activates WEE family kinases (WEE1 and PKMYT1) to induce G2-phase arrest, and inhibition of WEE family kinases potentiates proteasome inhibitor-induced pyroptosis by promoting mitotic entry. The combined inhibition of proteasome and WEE family kinases represents a promising new strategy for cancer therapy (Fig. 8k).

## Disscusion

Stimulation of mitotic catastrophe is considered as an attractive strategy to selectively kill cancer cells. Aberrant mitosis drives cells to mitotic catastrophe.^1,3^ During M-phase, increased chromatin compaction, loss of long-range intrachromosomal interactions, and displacement of many transcription regulators result in decreased RNA synthesis.^13,14^ The cap-dependent translation is also repressed.^15,16^ These alterations in M-phase make the proteasome-mediated protein degradation more crucial in the regulation of mitosis-associated proteins. Nevertheless, whether inhibition of proteasome function can affect the fates of M-phase cells and activate pyroptosis in mitosis remains unknown. Here, we revealed that proteasome inhibitor induced mitotic aberrations, including multi-polar spindle formation and mitotic arrest, and then drove cells to pyroptosis without exiting mitosis. These observations support that inhibitor of proteasome, like BTZ, may kill cancer cells via eliciting mitotic catastrophe in a form of mitotic pyroptosis.

Previous studies often defined apoptosis as the cell death form of mitotic catastrophe.^2^ Combined CHK1 and MK2 inhibition synergistically induces mitotic apoptosis in *KRAS*-mutant cancer cells.^45^ During taxol-induced mitotic arrest, cGAS functions to promote apoptosis via accumulating phosphorylation of IRF3.^7^ Speeding up mitosis by abrogation of the spindle checkpoint causes a temporal overlap of the enzymatic activities of NEK2A and separase, and consequent apoptosis during M-phase.^8^ Different from apoptosis, pyroptosis is characterized as a type of lytic cell death which is uniquely mediated by the gasdermin family.^9^ It remains unknown whether pyroptosis can be a death form of mitotic catastrophe. We found that either BTZ monotreatment or combined BTZ and PD0166285 (BP-Combo) treatment elicited significant mitotic pyroptosis but very few apoptosis, supporting pyroptosis as a new endpoint of mitotic catastrophe.

BTZ has been approved for the treatment of multiple myeloma and mantle cell lymphoma.^25,27^ Despite its success in treating hematological malignancies, BTZ did not received satisfactory therapeutic effect on solid tumors.^25,27^ Therefore, optimization of combination regimens may help to overcome the resistance of BTZ monotreatment in solid tumors. Here, we showed that BTZ treatment not only induced mitotic pyroptosis, but also substantially triggered G2-phase arrest, which was mediated by WEE family kinases, including WEE1 and PKMYT1. PD0166285, the inhibitor of WEE1 and PKMYT1, effectively abrogated the BTZ-induced G2-phase arrest, promoted M-phase entry, and remarkably exacerbated BTZ-stimulated pyroptosis. Combined BTZ and PD0166285 treatment showed synergistic lethality effect in repressing in vivo tumor growth and metastasis, and prolonged survival of tumor-bearing mice. Furthermore, the combined treatment induced a strong synergistic lethality in multiple solid tumor/transformed cell lines, but not in immortalized/normal cell lines, and BP-Combo has no obvious side effects in treated mice, indicating BP-Combo as a potential therapeutic regimen to specifically kill cancer cells. The reasons why cancer cells are more sensitive to BP-Combo treatment than untransformed cells may be attributed to: 1. Aberrant mitosis triggers MC. Tumor-specific alterations, like tetraploidy, aneuploidy, centrosome amplification and impaired SAC may render cancer cells more prone to mitotic aberrations and hence more sensitive to MC than untransformed cells.^4,6^ 2. Cancer cells are addicted to enhanced proteasome activity for efficient protein turnover to support their survival and proliferation.^22–25^

We found that the proteasome inhibitor-induced G2-phase arrest limited mitotic death, and inhibitors of WEE family kinases but not checkpoint kinase 1/2 (CHK1/2) or their upstream ATR released the BTZ-treated cells from G2 to M-phase. This may result from two reasons: 1) Only CHK1 of CHK1/2 was upregulated upon BTZ treatment, while two main members (WEE1 and PKMYT1) of WEE family were increased by BTZ treatment; 2) WEE family kinases directly phosphorylated CDK1 at Thr14/Tyr15 to inactivate CDK1 and prevent premature mitotic entry, while ATR/CHK1/2 indirectly inhibited CDK1 activity via regulating phosphatase CDC25.^46^ We also noticed that PD0166285, the inhibitor of both WEE1 and PKMYT1, was more powerful than MK-1775, the inhibitor of WEE1, in relieving BTZ-induced G2-phase arrest. Interestingly, we found that the expression level of PKMYT1 but not WEE1 were extensively upregulated in various human malignancies according to the TCGA data. In addition, the compensatory upregulation of PKMYT1 has been demonstrated to promote acquired resistance to MK-1775.^35,47,48^ These evidences indicate the unignorable role of PKMYT1 and suggest that combination of BTZ with PD0166285 may be more optimal than with MK-1775 in anti-cancer therapy. We also noted that compared to BTZ or PD0166285 monotreatment, BP-Combo significantly lessened the IC50 of both BTZ and PD0166285, which is important for reducing their side effect, especially for PD0166285, since the clinical development of WEE kinase inhibitor has been hampered by high rate of grade 3 toxicity.^35^

Proteasome and autophagy are two major systems responsible for the degradation of proteins.^49^ Inhibition of proteasome or overload of protein can activate autophagy to compensate the reduced capacity of proteasome and clear the threat arising from accumulation of toxic protein aggregates.^50^ Recently, it is shown that CDK1 functions to ensure a system-wide repression of autophagy during M-phase.^51^ We therefore speculate that when cells enter M-phase in the presence of proteasome inhibitor, autophagy is also repressed by the activated CDK1, which may cause cells to undergo persistent exposure to toxic protein aggregates, and this may be one of the reasons driving proteasome inhibitor to induce MC.

Although BP-Combo shows significant effects in selectively killing cancer cells and inhibiting tumor progression without obvious toxicity, more assessments in animal models and clinical trials are required to evaluate the effectiveness and toxicity of BP-Combo before clinical application. Moreover, the resistance to either BTZ or PD0166285 may also elicit resistance to BP-Combo strategy, like: 1. The mutations in the highly conserved binding pocket targeted by BTZ in cancer cells;^52^ 2. The overexpression of proteasome subunits β5 and other subunits, such as β2 and β1;^53^ 3. The induction of protein chaperones, like heat shock proteins, and other pathways for proteolysis, such as autophagy;^54–56^ 4. Mutations or overexpression of WEE1 family kinases;^47^ 5. The decrease of CDK1 or CDC25C activity, which prevents mitotic entry;^57,58^ 6. Activation of DNA damage repair genes.^36^

To our knowledge, this is the first study to disclose the effect of proteasome inhibition in inducing pyroptosis in mitosis, to characterize intrinsic pyroptosis as a new death form of MC, and to identify the combined inhibition of proteasome and WEE family kinases as an attractive strategy to specifically kill cancer cells, which is of great clinic significance and merits further investigations for cancer therapy.

## Materials and methods

Reagents, antibodies, siRNAs and details of experiments are provided in Supplementary Materials and Methods.

### Plasmids

The following plasmids were used: lentivirus expression vectors pCDH-H2B-EGFP, pCDH-BCL-xL and pCDH-CDK1, pCDH-CDK1 (T14A/Y15F) were generated using pCDH-CMV-MCS-EF1-copGFP (copepod green fluorescent protein) (System Biosciences, Palo Alto, CA, USA), which contained a copGFP expression cassette and was designated pCDH-Ctrl (control) in this study. pLenti6-mCherry-α-Tubulin was a gift from Dr. Zifeng Wang (Sun Yat-sen University Cancer Center). pT3EF1aH-myr-Akt and pT2-Caggs-N-RasG12V (gifts from Dr. Junfang Ji, Zhejiang University),^59^ pT3EF1aH-c-Myc (gift from Dr. Xin Chen, University of Hawaii Cancer Center),^60^ pX330-U6-sgTP53-CBh-hspCas9 (gift from Dr. Bin Zhao, Zhejiang University)^61^ and pCMV-SB11 (available via Addgene, plasmid number: 26552) were used for hydrodynamic injection.

### Cell lines

Human hepatoma cell line SNU449 and normal human skin fibroblast cells (SF) was maintained in RPMI 1640 medium (10-040-CVRC; Corning, New York, USA) supplemented with 10% fetal bovine serum (FBS, HyClone, Logan, UT, USA). The SV40 large T antigen-immortalized human bronchial epithelial cell line (HBE) and the H-Ras and SV40 small T antigen-transformed HBE cell line (HBERST) (gifts from Dr. Wen Chen, School of Public Health, Sun Yat-sen University),^62^ human cell lines from hepatoma (Huh1, SK-Hep-1), cervical adenocarcinoma (HeLa) or osteosarcoma (U2OS), immortalized human embryonic kidney (HEK) 293T cell line, immortalized human liver cell line L02, immortalized human hepatic stellate cell line (LX2) and mouse hepatoma cell line Hepa1-6 were cultured in Dulbecco’s modified Eagle’s medium (DMEM, 10-013-CVRC; Corning) supplemented with 10% FBS. All cells were grown with 1% penicillin/streptomycin (15140163; ThermoFisher Scientific) in a humidified atmosphere of 5% CO2 at 37 °C.

The SNU449 subline stably expressing pCDH-Ctrl (control), H2B-EGFP/mCherry-α-Tubulin, BCL-xL, CDK1 and CDK1 (T14A/Y15F) was constructed by infecting SNU449 cells with lentivirus that expressed the target sequences. SNU449 subline stably expressing H2B-EGFP/mCherry-α-Tubulin was screened with puromycin and sorting for EGFP^+^mCherry^+^ cells using flow cytometer (MoFlo Astrios EQs, Beckman Coulter, Miami, FL, USA).

### Cell transfection

RNA oligoribonucleotides were reversely transfected into cells using Lipofectamine RNAiMAX (Invitrogen, Carlsbad, CA, USA). The final concentration of RNA duplex was 20 nM.

### Analysis of gene expression

The mRNA and protein levels of target genes were analyzed by real-time quantitative polymerase chain reaction (qPCR) and western blotting, respectively. The sequences of primers for qPCR are provided in Supplementary Table 2.

### Cell cycle analysis

Cells were stained with pH3-S10 to indicate M-phase cells and with propidium iodide (PI) to indicate cellular DNA content, then subjected to flow cytometry analysis (FACS; Gallios, Beckman Coulter). Cellular DNA content (PI staining) is plotted against pH3-S10 signal. G2/M-phase cells were further separated into pH3-S10-positive (M-phase cells, 4n) and -negative cells (G2-phase cells, 4n).

### Live cell imaging

To monitor mitosis, SNU449 cells stably expressing H2B-EGFP and mCherry-α-Tubulin were seeded on a CellCarrier-96 Ultra plate (6055302; PerkinElmer, Waltham, MA, USA), and placed in an incubator chamber of the Operetta CLS High Content Analysis System and maintained in a 5% CO2 atmosphere at 37 °C (PerkinElmer). Images were captured at 20 min interval using 40× objective lens. Mitotic duration was calculated as the interval between nuclear envelope breakdown (NEBD, indicated by the first evidence of chromosome condensation) to the onset of anaphase or the occurrence of mitotic death. The fate of each cell was tracked and cell death was determined by the emergence of pyroptosis characteristics or loss of cell attachment.

### Immunofluorescence staining

Cells were fixed with 4% paraformaldehyde, permeabilized with 0.2% Triton X-100, pre-incubated in blocking buffer (1% BSA in PBS containing 0.1% Tween-20) for 30 min, incubated with antibody against tubulin, pericentrin or GSDME for 1 h at room temperature, and then with Alexa Fluor 488-conjugated goat anti-mouse IgG or Alexa Fluor 555-conjugated donkey anti-rabbit IgG, followed by counterstaining with 4’,6’-diamidino-2-phenylindole (DAPI, Sigma-Aldrich) and photographing using a confocal microscope (TCS SP8; Leica, Wetzlar, Germany).

### Cell death analysis

To calculate the proportion of pyroptosis or apoptosis cells, the images of cells were captured under phase-contrast brightfield after treatment. Five random fields in each well of a 12 well plate were captured under a 20× objective lens and then subjected to analysis for the rate of cells with pyroptosis or apoptosis morphology. Around 1000–3000 cells were counted for each well.

Pyroptotic or apoptotic cells were distinguished according to their typical morphology respectively. For pyroptosis, the dying cells showed evident swelling with characteristic large ballooned bubbles and became flattened and semi-transparent.^12,63^ For apoptosis, it is featured by cell shrinkage, membrane blebbing, breakage of cells and the subsequent formation of membrane-bound apoptotic bodies.^12,63^

### LDH release assay

The release of LDH was measured using the CytoTox 96 Non-Radioactive Cytotoxic Assay Kit (G1780; Promega, Madison, WI) and calculated as follows: LDH release (%) = (extracellular LDH/total LDH) × 100%. Total LDH includes the extracellular and intracellular LDH.

### Annexin V/Propidium Iodide (PI) double staining

Cells were stained using an Annexin V-Alexa 647/PI Apoptosis Detection Kit (FXP023-050; 4Abio, Beijing, China), followed by FACS analysis.

### Cell viability measurement

Cells (5000/well) were seeded into a 96-well plate for 24 h, then treated with 0.01–0.2 μM of BTZ and 0.1–4 μM of PD0166285 for 48 h before cell viability analysis using Alamar blue kit (TL-Y056B; Telenbiotech, Guangzhou, China).

### Colony formation assay

Cells (1000 SNU449, 1500 Huh1 and 1500 HeLa) were cultured in a 24-well plate. Cell culture media containing the indicated compounds were refreshed every three days for 10 days. Colonies were fixed in methanol, stained with a 0.1% crystal violet solution in 20% methanol for 15 min before photographing. To relatively quantify the survived colonies, the crystal violet was redissolved in methanol, and the optical density of each well was measured at 570 nm (OD570) using Varioskan LUX Multimode Microplate Reader (ThermoFisher Scientific).

### Detection of bortezomib concentration

The concentration of bortezomib in xenograft tissues of HeLa cells was measured using ultraperformance liquid chromatography/tandem mass spectrometry (UPLC-MS/MS, Guangzhou Multispectral Technology Co., Ltd).

### Mouse subcutaneous xenograft models

Female BALB/c nude mice and male C57BL/6J mice at four weeks of age were used for HeLa and Hepa1-6 xenografts, respectively.

To evaluate the effects of drug treatments at early stage of tumor development, HeLa cells (5 × 10^6^) were resuspended in 100 μL DMEM/Matrigel (3432-005-01; R&D Systems, Minneapolis, MN, USA) and then subcutaneously injected into both side of the posterior flanks of 11 mice and only on right side of one mouse. The day when tumor cells were inoculated (day 0), mice were randomly divided into four groups, and then intraperitoneally injected on day 1, 4 and 7 with vehicle (PBS), BTZ (0.65 mg/kg diluted in PBS), PD0166285 (0.26 mg/kg diluted in PBS), or combined BTZ (0.65 mg/kg) and PD0166285 (0.26 mg/kg). Mice were sacrificed on day 30 (Supplementary Fig. 9c).

To evaluate the effects of drug treatments at late stage of tumor development, HeLa cells (5 × 10^6^) or Hepa1-6 cells (5 × 10^5^) were resuspended in 100 μL DMEM/Matrigel (R&D Systems) and then subcutaneously injected into the right posterior flank of 13 or 16 mice. When tumor volumes reached ~50 mm^3^, mice were randomly divided into four groups and intraperitoneally treated with vehicle (PBS), BTZ (0.65 mg/kg), PD0166285 (0.26 mg/kg), or combined BTZ (0.65 mg/kg) and PD0166285 (0.26 mg/kg) every three days for five or six times (Supplementary Fig. 9d, e). Mice were sacrificed three days after the last treatment. Tumor growth was measured every three days, and the volume of tumor was measured with callipers and calculated as follows: volume = length × width^2^/2.

To verify the effect of GSDME in BP-Combo-induced tumor remission in vivo, Hepa1-6 cells (1 × 10^6^) which stably expressing shNC (control) or shGsdme (silencing Gsdme) were resuspended in 100 μL DMEM/Matrigel (R&D Systems) and then subcutaneously injected into the right posterior flank of 12 mice (shNC: *n* = 6; shGsdme: *n* = 6). For shNC or shGsdme, eight days after implantation when tumor volume reached approximately 50 mm^3^, mice were randomly divided into two groups and intraperitoneally administered with vehicle (PBS) or combined BTZ (0.65 mg/kg) and PD0166285 (0.26 mg/kg) every three days for a total of 5 times (Supplementary Fig. 9h). Tumor growth was measured every three days. Mice were sacrificed 23 days after implantation.

Tumors were photographed, weighed, and freshly frozen in liquid nitrogen.

### Mouse autochthonous liver tumor model

Male C57BL/6J mice at eight weeks of age were used. Plasmid mixture of pT3EF1aH-myr-Akt and pT2-Caggs-N-RasG12V, or pT3EF1aH-c-Myc and pX330-U6-sgTP53-CBh-hspCas9 (20 μg for each plasmid), together with 1.6 μg transposase-encoding vector (pCMV-SB11) were dissolved in saline (0.1 mL/g mouse body weight) and hydrodynamically injected into the tail veins of mice. Beginning on the 26th day after hydrodynamic injection, the mice were intraperitoneally administered with vehicle (PBS) or a combination of BTZ (1 mg/kg) and PD0166285 (0.26 mg/kg) three times a week for 11 times and then sacrificed on the 52th day (myr-Akt/NrasG12V-injecting mice) or for six times and then sacrificed on the 40th day (c-Myc/sgTP53-injecting mice) (Fig. 7a, b). Tumors, livers and lungs were collected and photographed. The average diameter of the top three tumor nodules was calculated as the maximal diameter of nodules. Lungs were stained overnight in picric acid solution (Sigma-Aldrich), and the number of metastatic nodules were counted. Thirty serial tissue sections from the liver and lung were stained with hematoxylin-eosin and then screened for metastatic foci independently by two researchers who were blinded to the treatment. The total number of primary tumor or metastatic foci and the maximal diameter (the average diameter of the largest three foci) among 30 serial sections were recorded.

### Mouse liver orthotopic xenograft model

Female C57BJ/6J mice at five weeks of age were used. Hepa1-6 cells (2 × 10^5^) were resuspended in 25 μL of 50% Matrigel (R&D Systems) and inoculated under the capsule of the left hepatic lobe. One week later, the mice were intraperitoneally administered with vehicle (PBS) or a combination of BTZ (1 mg/kg) and PD0166285 (0.26 mg/kg) three times a week for eight times before sacrificed (Fig. 8a). The primary tumors, livers, lungs, hearts, kidneys and small intestines (ilea) were dissected, fixed in formalin, embedded in paraffin, and sectioned.

To evaluate the metastasis of xenografts, 30 serial sections from the liver excluding the lobe with primary tumor and from the lung were stained with hematoxylin-eosin and then screened for metastatic foci independently by two researchers who were blinded to the treatment. The total number of metastatic foci from 30 serial sections were calculated.

To assess the toxicity of combined BTZ and PD0166285 treatment, mouse bone marrow cells were isolated from the femur by centrifugation at 380 *×* *g* for seven minutes, followed by resuspended in FACS buffer (1% FBS in 1× PBS). Approximately 1 × 10^6^ cells were stained with Brilliant Violet 421-tagged anti-mouse CD117 antibody, FITC-tagged anti-mouse Ly-6A/E (Sca1) antibody and PerCP-Cy5.5-tagged anti-mouse Lineage Cocktail, followed by FACS (Gallios, Beckman Coulter). The tissue sections of heart, kidney and ilea were stained with hematoxylin-eosin, and the villi length of ilea was measured. Body weight was measured three times weekly and fecal character was observed.

### Study approval

All mouse experiments were approved by the Institutional Animal Care and Use Committee at Sun Yat-sen University (ethical number: SYSU-IACUC-2022-B1173). All procedures for animal experiments were performed in accordance with the NIH Guide for the Care and Use of Laboratory Animals (National Academies Press, 2011) and according to the institutional ethical guidelines for animal experiments.

### Bioinformatics and statistical analysis

Gene expression profiling data were collected from The Cancer Genome Atlas (TCGA) (https://portal.gdc.cancer.gov/). Data are expressed as the mean ± standard error of the mean (SEM) of at least three independent experiments. Student’s *t* test was used to compare the differences between two groups. One-way ANOVA (analysis of variance) was used to compare the means of a dependent variable across three or more groups, typically evaluating the impact of one independent variable, while two-way ANOVA was applied to evaluate the impact of two independent variables. Kaplan–Meier survival curves and the log-rank test was used for survival analysis. A *P* value of less than 0.05 was considered the criterion of statistical significance, and all statistical tests were two-sided. These analyses were performed using GraphPad Prism version 8.0 software (GraphPad Software, Inc., San Diego, CA, USA). Loewe plots were plotted using the R package synergyfinder (version 3.4.5) by R studio (version 1.4.1106, R Studio Inc. Boston, MA).

### Supplementary information

Supplementary material

Original data

## Data Availability

This study did not generate any unique datasets or codes. The data generated in this study are available within the article and its supplementary data files.
