# Supplementary material for: Synergistic induction of mitotic pyroptosis and tumor remission by inhibiting proteasome and WEE family kinases
Source: Signal Transduct Target Ther. 2024 Jul 12;9:181. doi: 10.1038/s41392-024-01896-z (PMC11239683; doi:10.1038/s41392-024-01896-z)
Supplement: Supplementary file 1 — Supplementary material [file 41392_2024_1896_MOESM1_ESM.docx]

Supplementary Materials for

**Synergistic induction of mitotic pyroptosis and tumor remission by inhibiting proteasome and WEE family kinases**

Zhan-Li Chen, Chen Xie, Wei Zeng, Rui-Qi Huang, Jin-E Yang, Jin-Yu Liu, Ya-Jing Chen, Shi-Mei Zhuang

Correspondence to: zhuangshimei@163.com or LSSZSM@mail.sysu.edu.cn

**This file includes:**

Materials and Methods

Supplementary Figures 1-10

Supplementary Tables 1-2

**Materials and Methods**

**Reagents**

Bortezomib (S1013), Carfilzomib (S2853), RO-3306 (S7747), Nocodazole (S2775), Rabusertib (S2626), Prexasertib (S7178), AZD6738 (S7693), MK-1775 (S1525), PD0166285 (S8148) were purchased from Selleckchem (Houston, TX, USA).

**Antibodies**

Mouse monoclonal antibody (mAb) against α-Tubulin (T9026; Sigma-Aldrich, St Louis, MO, USA), GAPDH (BM1623; Boster, Wuhan, China), CDK1 (9116T; Cell Signaling Technology, CST, Beverly, MA, USA); rabbit polyclonal antibody (pAb) against pericentrin (ab4448; Abcam, Cambridge, MA, USA), PSMC5 (ab178681; Abcam), Cleaved Caspase-3 (ab32042; Abcam), BCL-xL (2764T; CST), Ser10-phosphorylated histone H3 (9701L; CST), CHK2 (2662T; CST), PKMYT1 (4282T; CST); rabbit mAb against GSDME (ab215191; Abcam), CHK1 (ab40866; Abcam), WEE1 (13084T; CST), Tyr15-phosphorylated CDK1 (4539T; CST); Alexa Fluor 647-conjugated rabbit mAb against Ser10-phosphorylated histone H3 (3458S; CST); Alexa Fluor 488-conjugated goat anti-mouse IgG (A11008; ThermoFisher Scientific, MA, USA); Alexa Fluor 555-conjugated donkey anti-rabbit IgG (A31572; ThermoFisher Scientific); Brilliant Violet 421-tagged mouse mAb against CD117 (105828; BioLegend, San Diego, CA, US); FITC-tagged mouse mAb against Ly-6A/E (Sca1) (11-5981-82; eBioscience, San Diego, CA, USA); PerCP-Cy5.5-tagged anti-mouse Lineage Cocktail (561317; BD Biosciences, Franklin Lakes, NJ, USA).

**Vector Construction**

For pLenti6-mCherry-α-Tubulin, the fusion product of mCherry and α-Tubulin was cloned and inserted into the *Bam*HI/*Apa*I sites of pLenti6-VSV-Puro vector. To create pCDH-H2B-EGFP, the full-length of human histone H2B was cloned and fused with EGFP at C-terminal by fusion PCR and inserted into the *Bam*HI/*Xba*I sites of pCDH-CMV-MCS-EF1-Puro vector (System Biosciences, Mountain View, CA, USA). To create pCDH-BCL-xL and pCDH-CDK1, the full-length of human BCL-xL and CDK1 were cloned and inserted into the *Eco*RI/*Not*I and *Eco*RI/*Bam*HI sites of pCDH-CMV-MCS-EF1-Puro vector. The mutant CDK1-T14A/Y15F was generated through fusion PCR and inserted into the *Eco*RI/*Bam*HI sits of pCDH-CMV-MCS-EF1-Puro vector to construct pCDH-CDK1-T14A/Y15F. The sequences of primers for cloning are provided in Supplementary Table 2.

To construct the lentiviral vectors that expressed short hairpin RNAs (shRNAs) targeting mouse Gsdme (pCDH-shGsdme) and the control vector pCDH-shNC, the shRNA sequences (Supplementary Table S2), the spacer sequence (5’-TTC AAG AGA) and the flanking *Eco*RI and *Bam*HI sites were chemically synthesized, annealed, and then inserted into the *Eco*RI/*Bam*HI sites of the pCDH-U6 vector. pCDH-U6 was produced based on the pCDH-Ctrl vector in which the CMV promoter was replaced by the U6 promoter.

**RNA Oligoribonucleotides**

The small interfering RNAs (siRNAs) targeting human PSMC1 (GeneBank accession No. NM_002802.3), PSMC2 (NM_002803.4), PSMC3 (NM_002804.5), PSMC4 (NM_006503.4), PSMC5 (NM_002805.6), PSMC6 (NM_002806.5), Rpn10 (NM_002810.4), Rpn11 (NM_005805.6), Rpn13 (NM_007002.4), GSDMB (NM_178171.5), GSDMD (NM_024736.7), GSDME (NM_004403.3), Caspase-1 (NM_001223.5), Caspase-3 (NM_001354777.2), cGAS (NM_138441.3), CHOP (NM_001195053.1) and IκBα (NM_020529.3) transcripts were designated as siPSMC1, siPSMC2, siPSMC3, siPSMC4, siPSMC5, siPSMC6, siRpn10, siRpn11, siRpn13, siGSDMB, siGSDMD, siGSDME, siCaspase-1, siCaspase-3, sicGAS, siCHOP and siIκBα, respectively. The negative control (NC) RNA duplex for siRNA was non-homologous to any human genome sequences. All RNA oligoribonucleotide were purchased from Ribo-bio (Guangzhou, China) and their sequences are provided in Supplementary Table 2.

**Lentivirus Production**

To produce lentivirus, HEK293T cells were co-transfected with lentivirus expression vector containing target sequence and packing plasmid mix (Lenti-X HTX Packaging Mix; Clontech, Palo Alto, CA, USA) by calcium phosphate precipitation. The lentivirus supernatant was harvested and stored in aliquots at -80℃ until use.

**LDH Release Assay**

Cells (5000/well) were seeded into a 96-well plate and treated with the indicated compounds or transfected with the indicated RNA duplexes. Two wells were included for each treatment. For extracellular LDH detection, 50 μL of cell culture supernatant was collected from one well. For total LDH detection, 10 μL of 10×Lysis Solution was added to another well and incubated for 45 minutes, then the supernatant was collected. Next, the supernatant was mixed with 50 μL of the CytoTox 96® Reagent for 30 minutes, followed by addition of 50 μL Stop Solution and the measurement of optical density at 490 nm (Varioskan LUX Multimode Microplate Reader, ThermoFisher Scientific).

**Cell Cycle Analysis**

Cells were pelleted, washed in cold phosphate-buffered saline (PBS), fixed with 70% ethanol at -20℃ overnight, permeabilized with 0.25% Triton X-100 for 10 minutes, blocked with 1% BSA for 30 minutes, incubated with Alexa Fluor 647-conjugated antibody against Ser-10-phosphorylated histone H3 (pH3-S10) for 1 hours at room temperature, then washed twice with PBS and incubated with 1 mg/mL RNase A and 0.05 mg/mL propidium iodide (PI) for 20 minutes before flow cytometry analysis (FACS; Gallios, Beckman Coulter). pH3-S10 staining indicates cells at M-phase. Cellular DNA content (PI staining) is plotted against pH3-S10 signal.


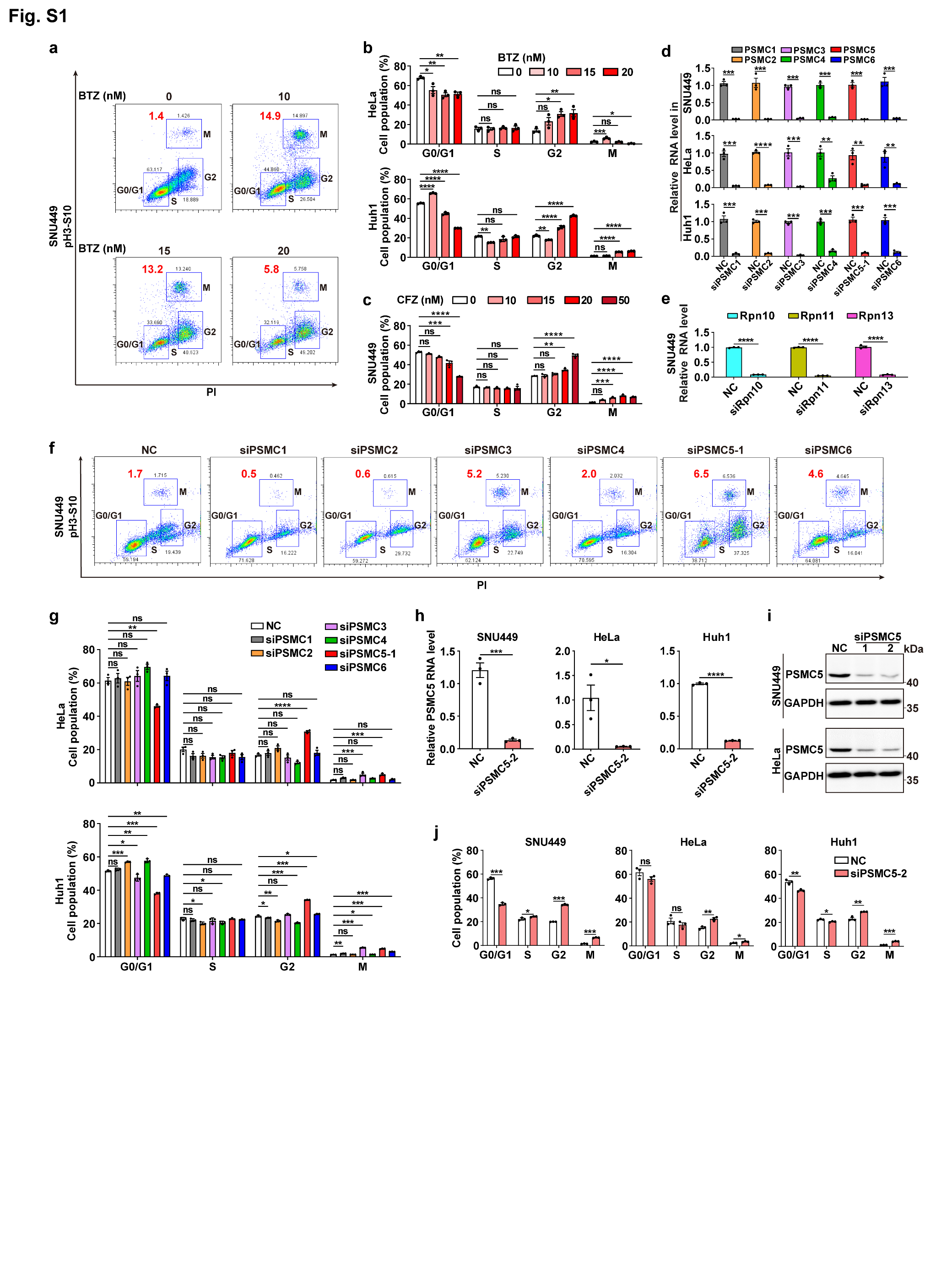


**Supplementary Fig. 1. Inhibition of proteasome induces M-phase arrest.** (**a**) Representative images of pH3-S10/PI staining for BTZ-treated SNU449 cells. (**b**) BTZ increased the proportion of G2- and M-phase cells. HeLa and Huh1 cells were treated with the indicated dose of BTZ for 24 and 30 hours, respectively, then stained for pH3-S10 to indicate M-phase cells and stained with PI to indicate DNA content, followed by FACS for the phase distribution of cell cycle (G0/G1, S, G2, M). (**c**) CFZ treatment increased the population of G2- and M-phase cells. SNU449 cells were treated with indicated doses of CFZ for 30 hours, then stained for pH3-S10/PI and subjected to FACS analysis. (**d-e**) The effects of siRNAs targeting AAA-ATPases or non-ATPases of 19S regulatory particles. Cells transfected with the indicated siRNAs were subjected to qPCR analysis. (**f**) Representative images of pH3-S10/PI staining for siRNA-transfected SNU449 cells. (**g**) Silencing PSMC5 increased the proportion of G2- and M-phase cells. HeLa and Huh1 cells were transfected with the indicated siRNAs, followed by pH3-S10/PI staining and FACS. (**h**-**i**) The cellular mRNA (h) and protein (i) levels of PSMC5 were reduced by siPSMC5-1 and siPSMC5-2. (**j**) The effect of PSMC5 knockdown on cell cycle was confirmed by siPSMC5-2. Numbers in red font indicate the proportion of M-phase cells (a, f). Error bars: SEM from at least three independent experiments. One-way ANOVA (b-c and g) and Student’s *t* test (d, e, h, j) were used. *, *P* < 0.05; **, *P* < 0.01; ***, *P* < 0.001; ****, *P* < 0.0001; ns, not significant.

**
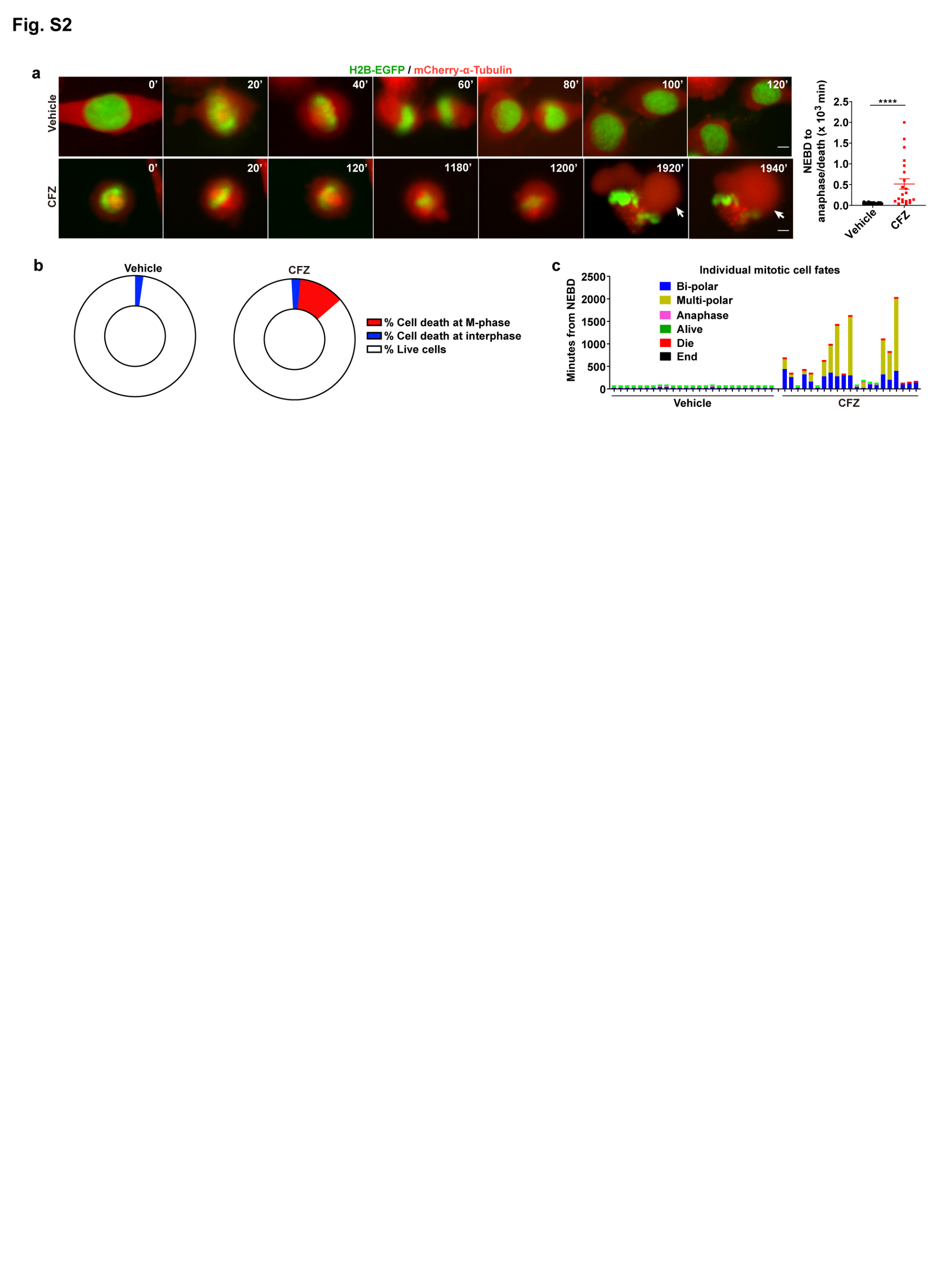
**

**Supplementary Fig. 2. Inhibition of proteasome by CFZ induces mitotic arrest, multi-polar spindle formation and ballooning bubbles.** SNU449 subline stably expressing histone H2B-EGFP and mCherry-α-tubulin was treated with 30 nM CFZ, followed by live cell imaging. For (**a**), the time from nuclear envelope breakdown (NEBD) to the end of anaphase or cell death was designated as mitotic duration (*right* panel; Vehicle, *n* = 67; CFZ, *n* = 21). White arrows indicate the large bubbles blowing from the plasma membrane. Scale bar, 5 μm. For (**b**), cell death was determined by the emergence of pyroptosis characteristics or cell detachment and the fractions of cells died at interphase or M-phase were quantified (Vehicle, *n* = 134; CFZ, *n* = 124). For (**c**), the fates of individual mitotic cell are shown. For (a, c), the time point of NEBD was set as 0. Error bars: SEM from at least three independent experiments. Student’s *t* test (a) was used. ****, *P* < 0.0001.

**
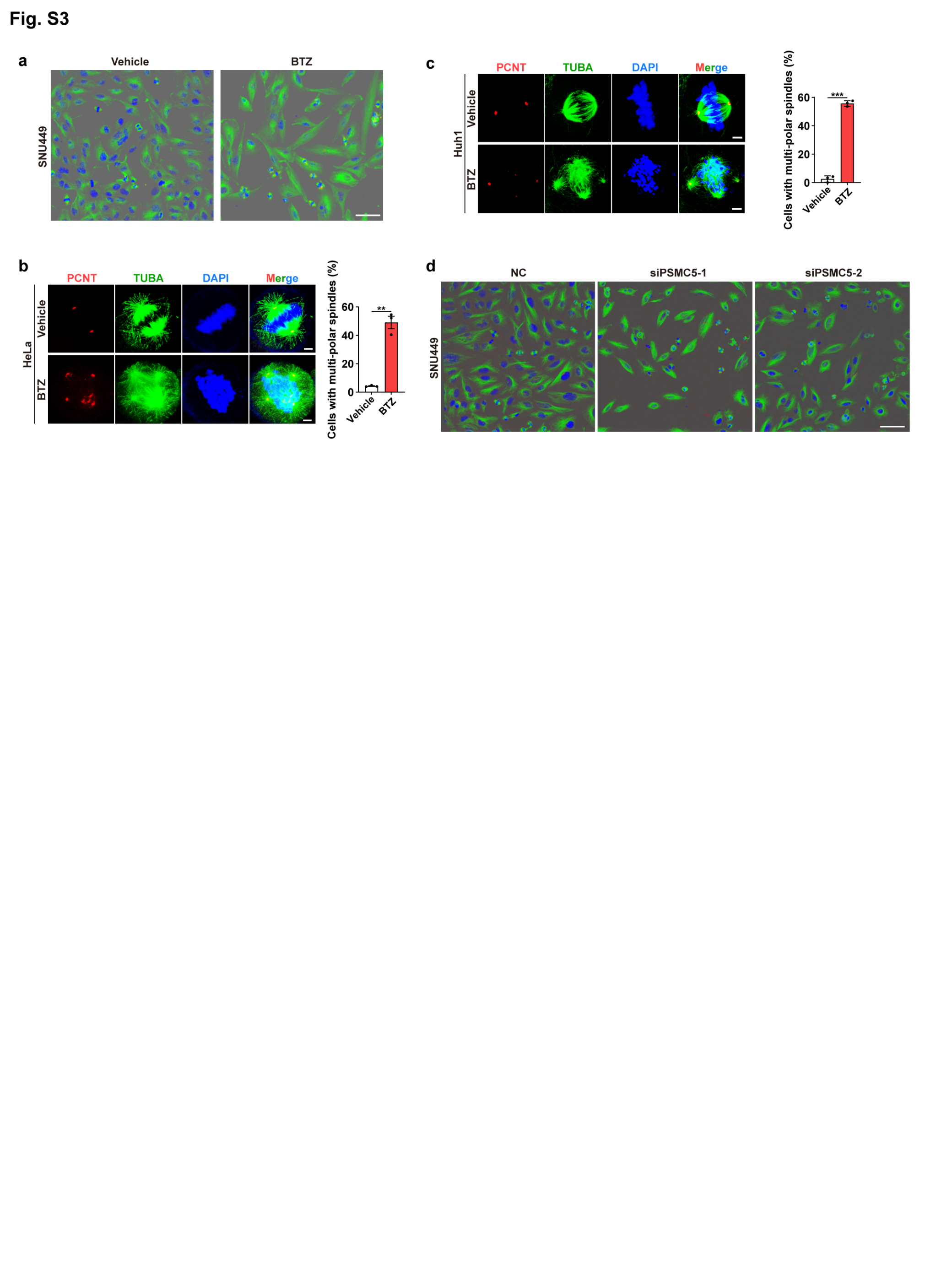
**

**Supplementary Fig. 3. Inhibition of proteasome induces multi-polar spindle formation.**  (**a-c**) BTZ induced multipolar spindles in tumor cells. (**d**) Knockdown of PSMC5 induced multipolar spindles in tumor cells. SNU449 (a), HeLa (b) and Huh1 (c) cells were treated with BTZ for 30 (SNU449) or 24 (HeLa and Huh1) hours, and SNU449 (d) cells were transfected with the indicated siRNAs for 60 hours, before staining for pericentrin (PCNT, red), α-Tubulin (TUBA, green) and nuclear (DAPI, blue) to indicate centrosome, spindle and chromosome, respectively. (a) and (d) are low-power images of immunofluorescence in Figure 1j-k. For (a, d), scale bar, 50 μm. For (b-c), scale bar, 2.5 μm. Error bars: SEM from at least three independent experiments. Student’s *t* test (b, c) was used. **, *P* < 0.01; ***, *P* < 0.001.

**
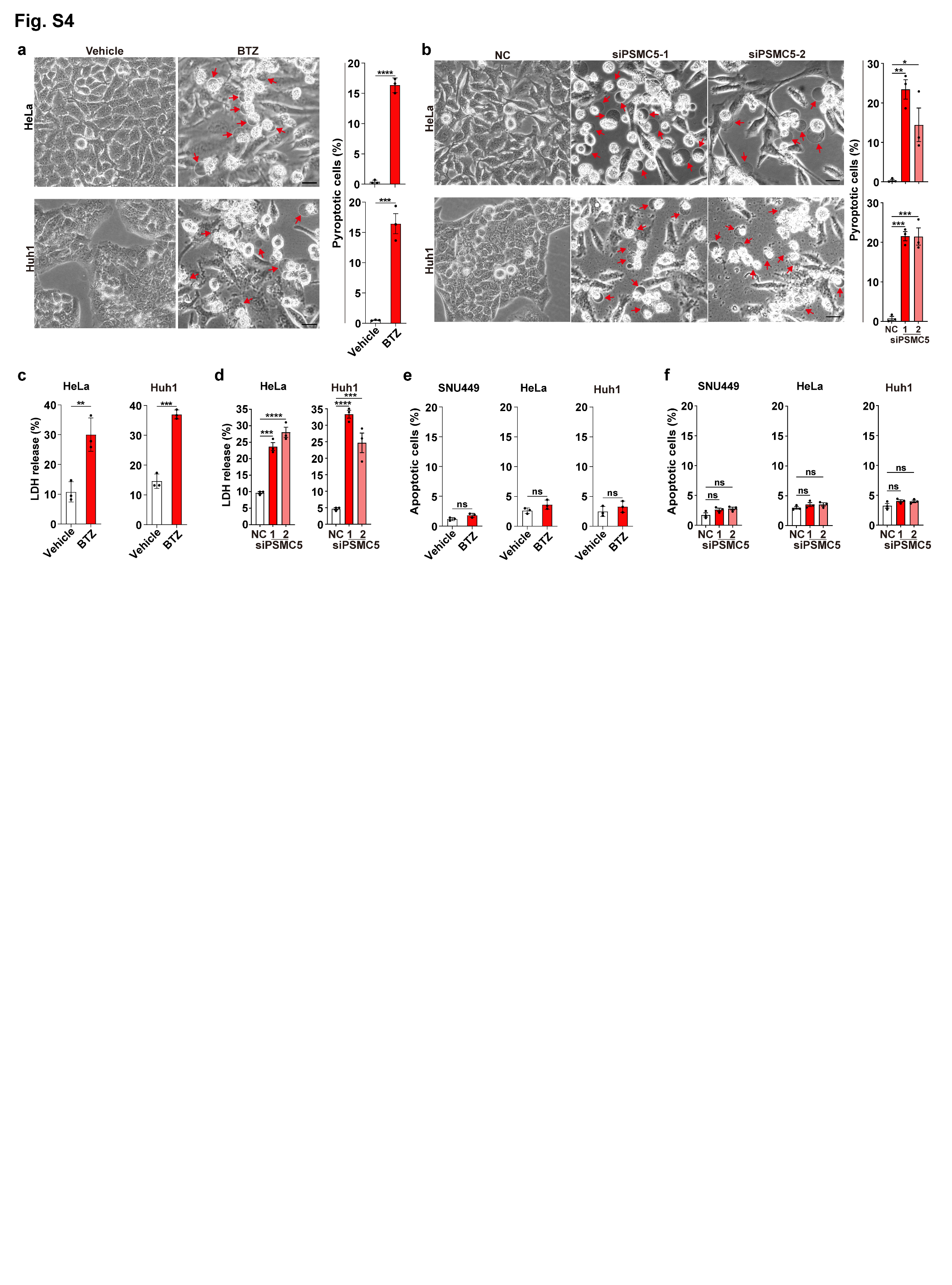
**

**Supplementary Fig. 4. Inhibition of proteasome induces pyroptosis.** (**a**-**b**) Proteasome inhibition induced morphology of pyroptosis. Red arrows indicate the pyroptotic cells with large bubbles. The proportion of pyroptotic cells was calculated (*right* panel). Five random fields in each well were captured and then subjected to analysis for the rate of cells with pyroptosis morphology. One of the five fields is shown as representative image for each group. Scale bar, 20 μm. (**c**-**d**) Proteasome inhibition stimulated LDH release. HeLa and Huh1 cells were exposed to 10 or 20 nM BTZ for 48 hours or transfected with NC, siPSMC5-1 or siPSMC5-2 for 72 hours before phase-contrast imaging (a-b) or LDH release assay (c-d). (**e-f**) Proteasome inhibition did not affect the proportion of apoptotic cells. Cells was counted based on images from Figure 2a-b and S4a-b. For (a-b) and (e-f), pyroptotic or apoptotic cells were distinguished according to their typical morphology. For pyroptosis, the dying cells showed evident swelling with characteristic large ballooned bubbles and became flattened and semi-transparent. For apoptosis, it is featured by cell shrinkage, membrane blebbing, breakage of cells and the subsequent formation of membrane-bound apoptotic bodies. Error bars: SEM from at least three independent experiments. Student’s *t* test (a, c, e) and one-way ANOVA (b, d, f) were used. *, *P* < 0.05; **, *P* < 0.01; ***, *P* < 0.001; ****, *P* < 0.0001; ns, not significant.

**
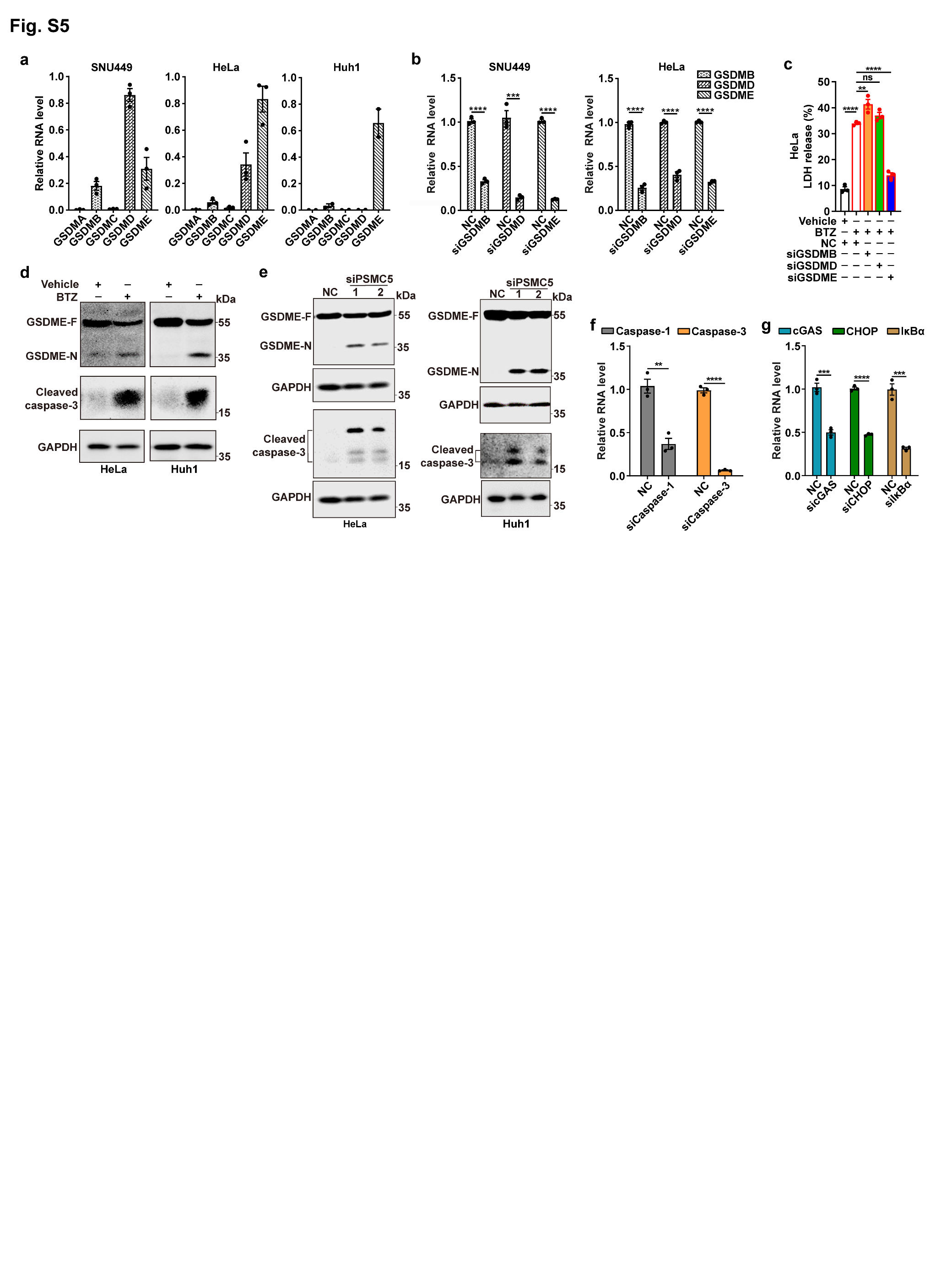
**

**Supplementary Fig. 5. GSDME mediates proteasome inhibition-induced pyroptosis.** (**a**) The mRNA levels of gasdermins in different cell lines. The total RNA of SNU449, HeLa and Huh1 cells were analyzed by qPCR assay. (**b**) The efficacy of siRNAs targeting gasdermins. Cells were transfected with NC or the indicated siRNAs for 48 hours before qPCR analysis. (**c**) GSDME knockdown attenuated BTZ-induced LDH release. HeLa cells were transfected with NC or siRNAs targeting the indicated gasdermins for 24 hours, then treated with vehicle or 10 nM BTZ for another 48 hours before LDH release assay. (**d-e**) BTZ or PSMC5 knockdown induced the cleavage of GSDME and caspase-3. HeLa or Huh1 cells were exposed to vehicle or BTZ for 48 hours or transfected with NC, siPSMC5-1 or siPSMC5-2 for 72 hours before immunoblotting. (**f-g**) The efficacy of siRNAs. RNA duplex targeting caspase-1 or caspase-3 (f), cGAS, CHOP and IκBα (g). Error bars: SEM from at least three independent experiments. Student’s *t* test (b, f-g) and one-way ANOVA (c) were used. **, *P* < 0.01; ***, *P* < 0.001; ****, *P* < 0.0001; ns, not significant.

**
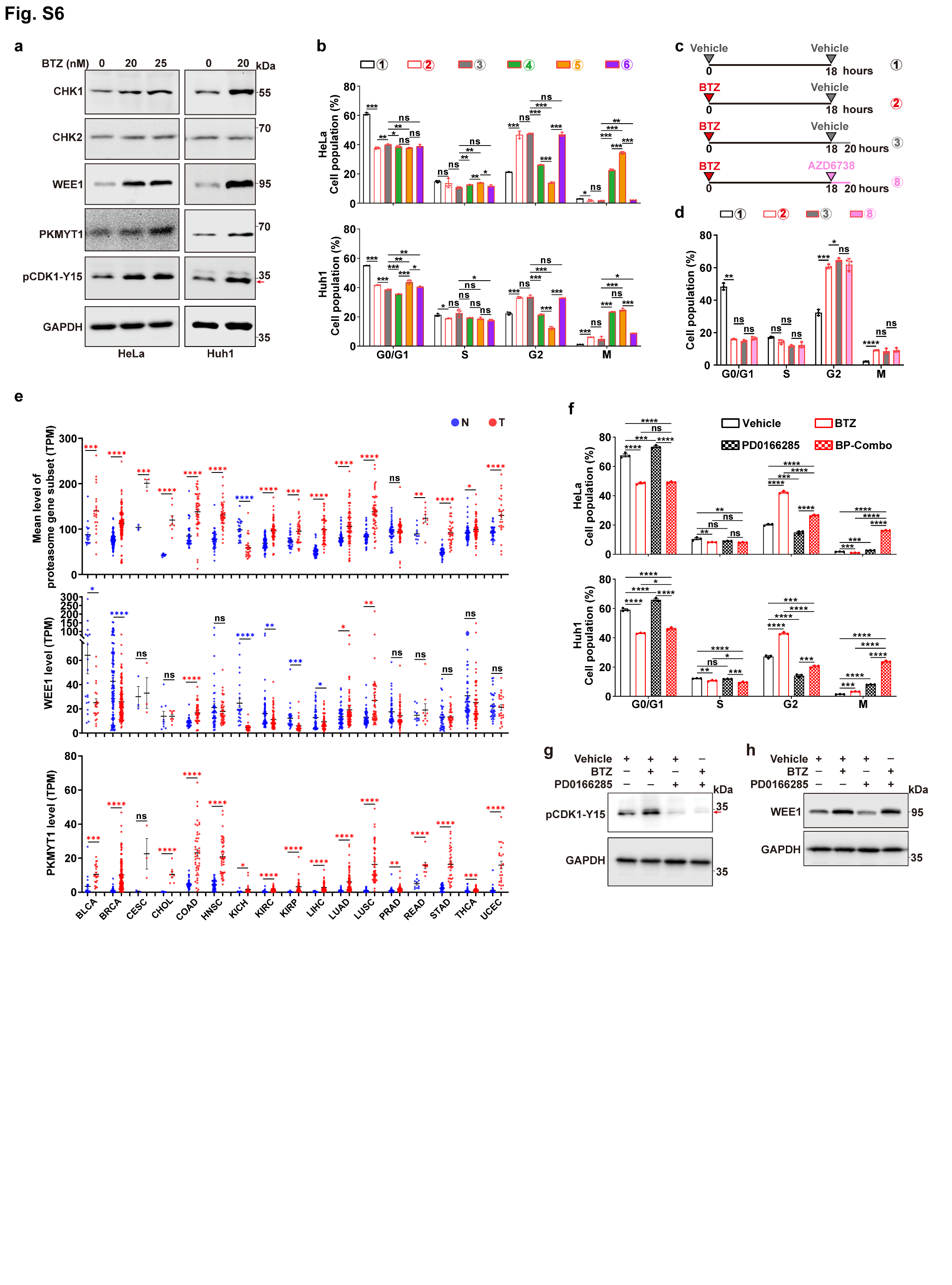
**

**Supplementary Fig. 6. PD0166285 abrogates BTZ-induced G2-phase arrest.** (**a**) BTZ increased the protein levels of CHK1, WEE1, PKMYT1 and inhibitory phosphorylation of CDK1 at Tyr-15. HeLa and Huh1 cells were treated with the indicated doses of BTZ for 18 hours before immunoblotting. (**b**) PD0166285 effectively alleviated BTZ-induced G2-phase arrest. Schematic diagrams of experiment design are shown in Figure 3b. HeLa or Huh1 cells were pretreated with vehicle or 20 nM BTZ for 18 hours, followed by treatment with vehicle or 0.5 μM indicated inhibitors for another 2 hours before pH3-S10/PI staining and FACS. (**c-d**) The ATR inhibitor AZD6738 failed to abrogate BTZ-induced G2-phase arrest. (**e**) The mean expression level of proteasome gene subset and the expression level of WEE1 and PKMYT1 were analyzed based on TCGA data. The subset of proteasome subunit genes included 20S core subunits (α1–7, β1–7) and 19S regulatory subunits (PSMC1–6). TPM, transcripts per kilobase per million mapped reads; N, adjacent non-tumor tissue; T, tumor tissue. Abbreviations: BLCA, bladder urothelial carcinoma; BRCA, breast invasive carcinoma; CESC, cervical squamous cell carcinoma and endocervical adenocarcinoma; CHOL, cholangiocarcinoma; COAD, colon adenocarcinoma; HNSC, head and neck squamous cell carcinoma; KICH, kidney chromophobe; KIRC, kidney renal clear cell carcinoma; KIRP, kidney renal papillary cell carcinoma; LIHC, liver hepatocellular carcinoma; LUAD, lung adenocarcinoma; LUSC, lung squamous cell carcinoma; PRAD, prostate adenocarcinoma; READ, rectum adenocarcinoma; STAD, stomach adenocarcinoma; THCA, thyroid carcinoma; UCEC, uterine corpus endometrial carcinoma. (**f**) Concurrent exposure to PD0166285 potentiated BTZ-induced accumulation of mitotic cells. HeLa and Huh1 cells were treated with vehicle, BTZ, PD0166285 or BP-Combo for 24 hours before pH3-S10/PI staining. (**g**) PD0166285 attenuated BTZ-induced accumulation of pCDK1-Y15. (**h**) PD0166285 can not affect BTZ-induced WEE1 accumulation. For (g-h), SNU449 cells were treated with vehicle, 20 nM BTZ, 0.25 μM PD0166285 or BP-Combo for 24 hours before immunoblotting. For (a) and (g), red arrows indicate the target band. Error bars: SEM from at least three independent experiments. One-way ANOVA (b, d-f) and Student’s *t* test (e) were used. *, *P* < 0.05; **, *P* < 0.01; ***, *P* < 0.001; ****, *P* < 0.0001; ns, not significant.

**
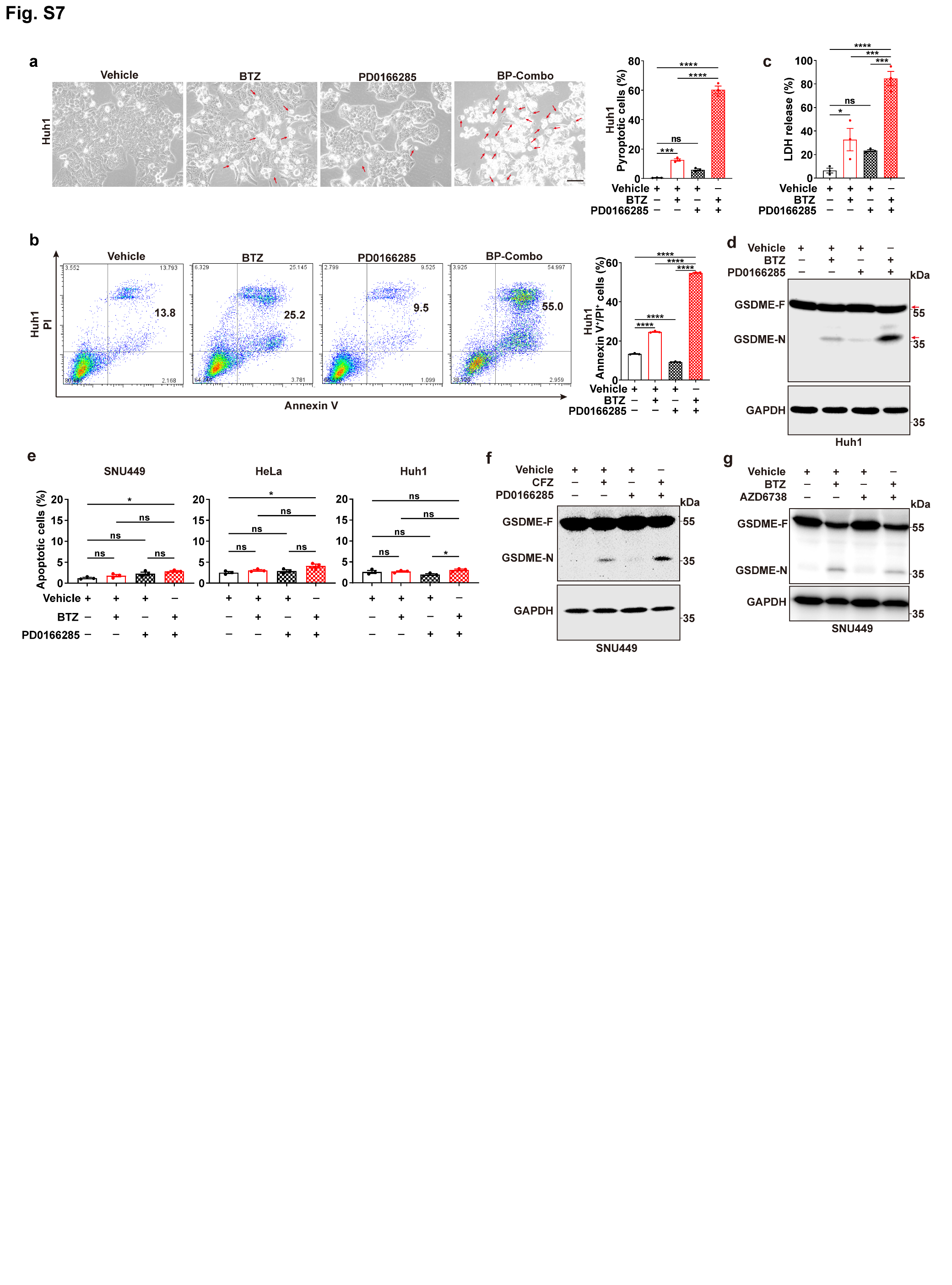
**

**Supplementary Fig. 7. PD0166285 augments proteasome inhibitor-induced pyroptosis.** (**a**) PD0166285 enhanced the role of BTZ in increasing the proportion of cells with pyroptosis morphology. Red arrows indicate the pyroptotic cells with large bubbles. Five random fields in each well were captured and then subjected to analysis for the rate of cells with pyroptosis morphology. One of the five fields is shown as representative image for each group. Scale bar, 50 μm. (**b)** PD0166285 promoted the role of BTZ in increasing the fraction of cells with Annexin V/PI double-staining. (**c**) PD0166285 enhanced the role of BTZ in promoting LDH release. (**d**) PD0166285 potentiated the role of BTZ in promoting GSDME cleavage. Red arrow indicates the target band. For (a-d), Huh1 cells were treated with vehicle, BTZ, PD0166285 or BP-Combo before phase-contrast imaging (a), Annexin V/PI staining (b), LDH detection (c) and immunoblotting assays (d). (**e**) A very low propotion of apoptotic cells was observed upon mono- or combined-BTZ and PD0166285 treatment. The proportion of apoptotic cells was calculate based on cell morphology of the brightfield images in Figure 4a and S7a. (**f**) PD0166285 promoted CFZ-induced GSDME cleavage. (**g**) AZD6738 can not enhance BTZ-induced GSDME cleavage. For (f-g), SNU449 cells were treated with mono- or combined-CFZ/PD0166285, or BTZ/AZD6738 for 48 hours before western blotting. Error bars: SEM from at least three independent experiments. One-way ANOVA (a-c and e) was used. *, *P* < 0.05; ***, *P* < 0.001; ****, *P* < 0.0001; ns, not significant.

**
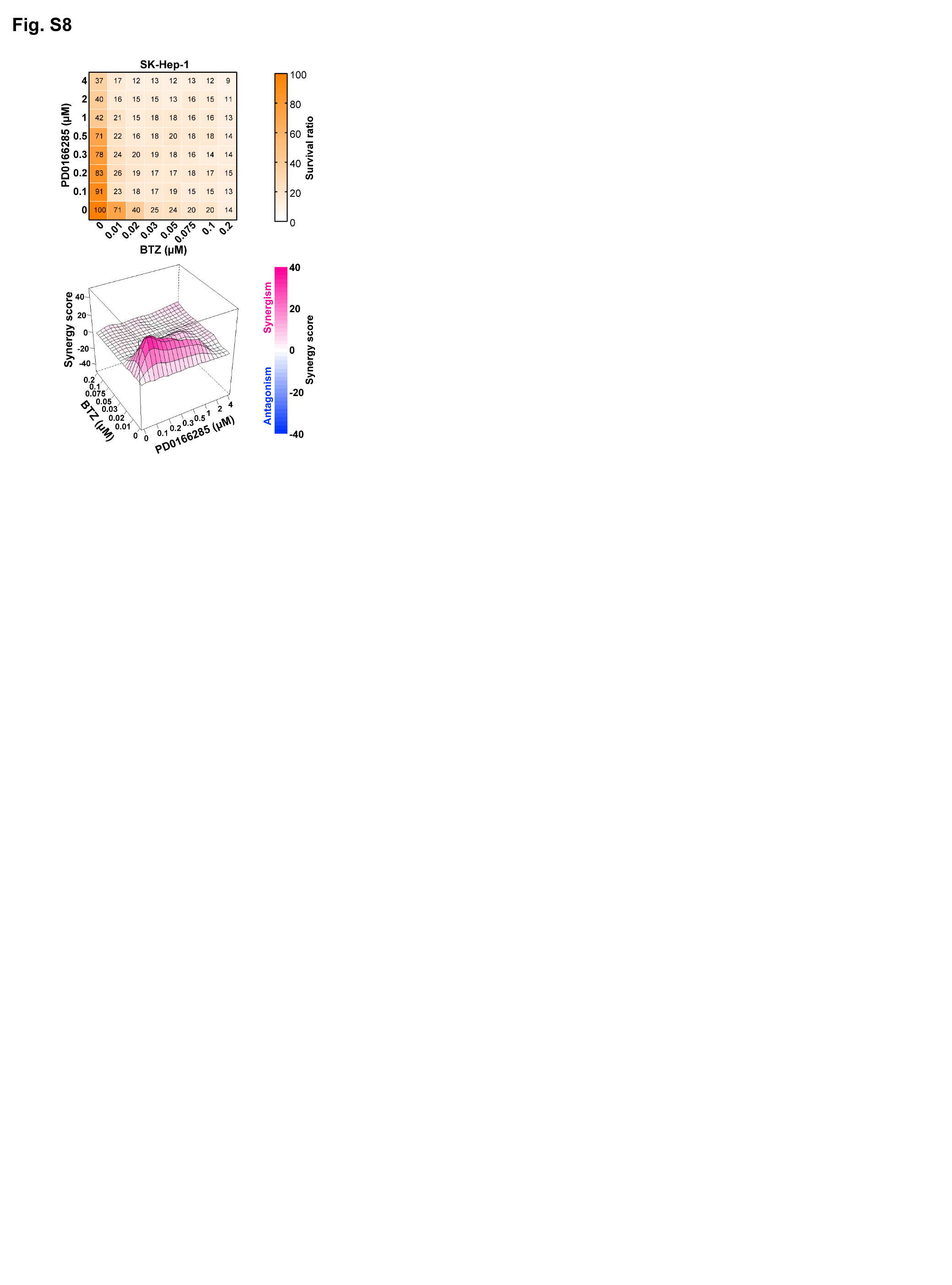
**

**Supplementary Fig. 8. BTZ and PD0166285 shows synergistic effect in killing SK-Hep-1 cells.** Cells were treated for 48 hours with a combination of BTZ and PD0166285 at the indicated concentration. Cell survival was measured by Alamar Blue assay. Cooperativity screens (*upper* panels) and Loewe plots (*down* panels) for the synergistic effect of BTZ and PD0166285 are shown based on at least three independent experiments. In *upper* panels, color bars indicate the percentage of surviving cells in BP-Combo-treated group, which was normalized to untreated group. In *down* panels, color bars indicate synergy score in the Lowe plots; a score greater than 0 indicates synergism, and less than 0 indicates antagonism.

**
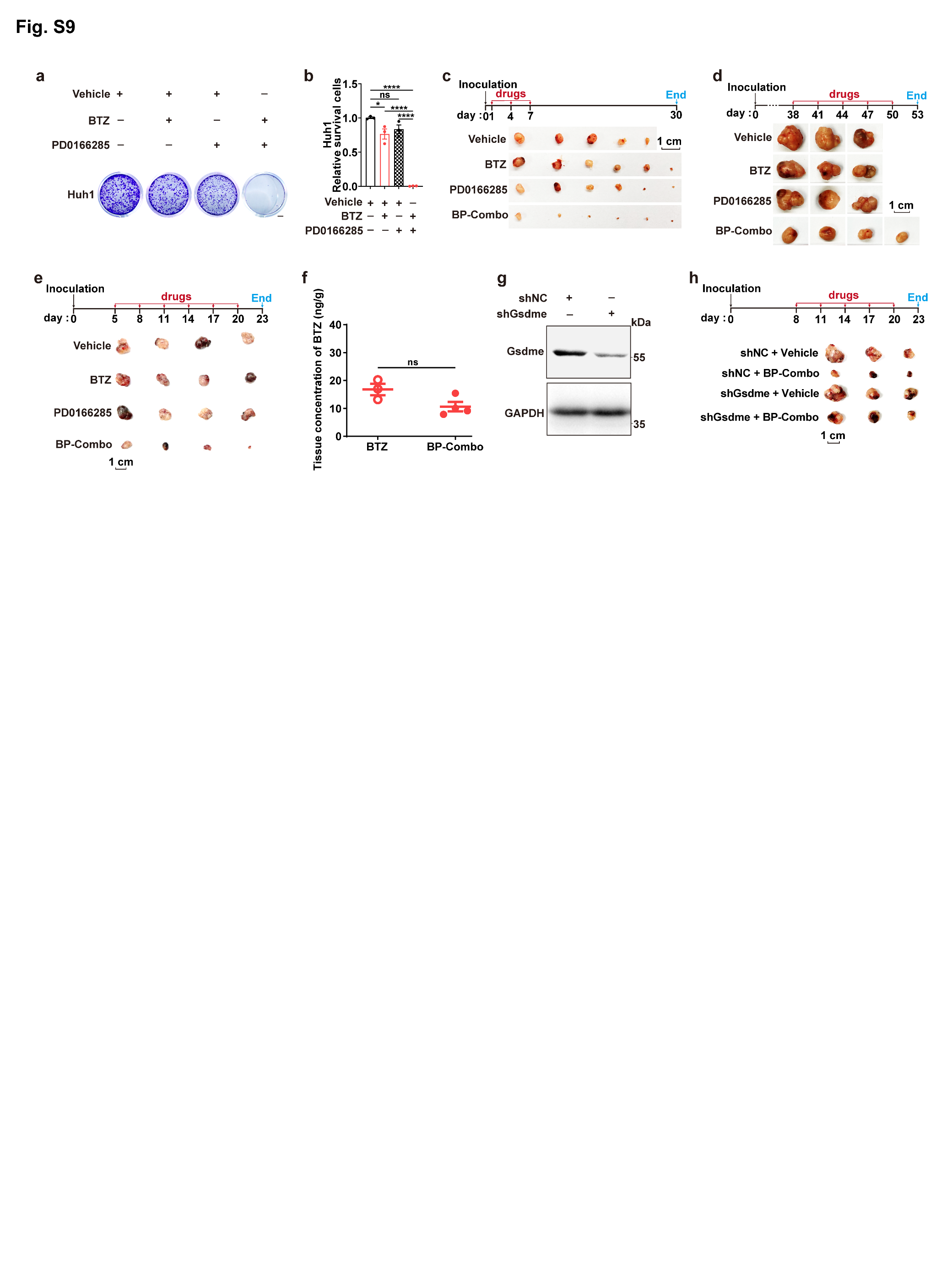
**

**Supplementary Fig. 9. BTZ and PD0166285 have a synergistic effect in suppressing the growth of subcutaneous tumor xenografts.** (**a**-**b**) BP-Combo showed much stronger effect than BTZ or PD0166285 monotreatment in repressing colony formation of tumor cells. Huh1 cells were exposed to the indicated treatment for 10 days, then stained with 0.1% crystal violet. The representative images (a) and colony quantification (b) are shown. Scale bar, 2 mm. (**c-e**) BP-Combo showed much stronger effect than BTZ or PD0166285 monotreatment in inhibiting tumor xenograft development. BALB/c nude mice were subcutaneously injected with HeLa cells, and then intraperitoneally injected with the indicated inhibitors one day after tumor cell implantation (early treatment, c), or when tumor volumes reached ~50 mm^3^ (late treatment, d). Early treatment: *n* = 5 (vehicle), 6 (BTZ), 6 (PD0166285) and 6 (BP-Combo). Late treatment: *n* = 3 (vehicle), 3 (BTZ), 3 (PD0166285) and 4 (BP-Combo). For (e), C57BL/6J mice were subcutaneously injected with Hepa1-6 cells, and then intraperitoneally injected with the indicated inhibitors when tumor volumes reached ~50 mm^3^. *n* = 4 for each group. (**f**) Combine treatment with PD0166285 did not increase the concentration of BTZ in tumor tissues. The concentration of BTZ in the tumor tissues of BTZ and BP-Combo groups was detected by LC-MS/MS analysis. (**g**) Silencing of Gsdme in mouse hepatoma cells. The Hepa1-6 cells with stable expression of shGsdme or their control lines were analyzed. (**h**) Silencing of Gsdme diminished the anti-tumor effect of BP-Combo. C57BL/6J mice were subcutaneously injected with Hepa1-6-shNC or Hepa1-6-shGsdme cells, and then intraperitoneally administered with vehicle or BP-Combo treatment when tumor volumes reached ~50 mm^3^. *n* = 3 for each group. Error bars: SEM from at least three independent experiments. One-way ANOVA (b) and Student’s *t* test (f) were used. *, *P* < 0.05; ****, *P* < 0.0001; ns, not significant.

**
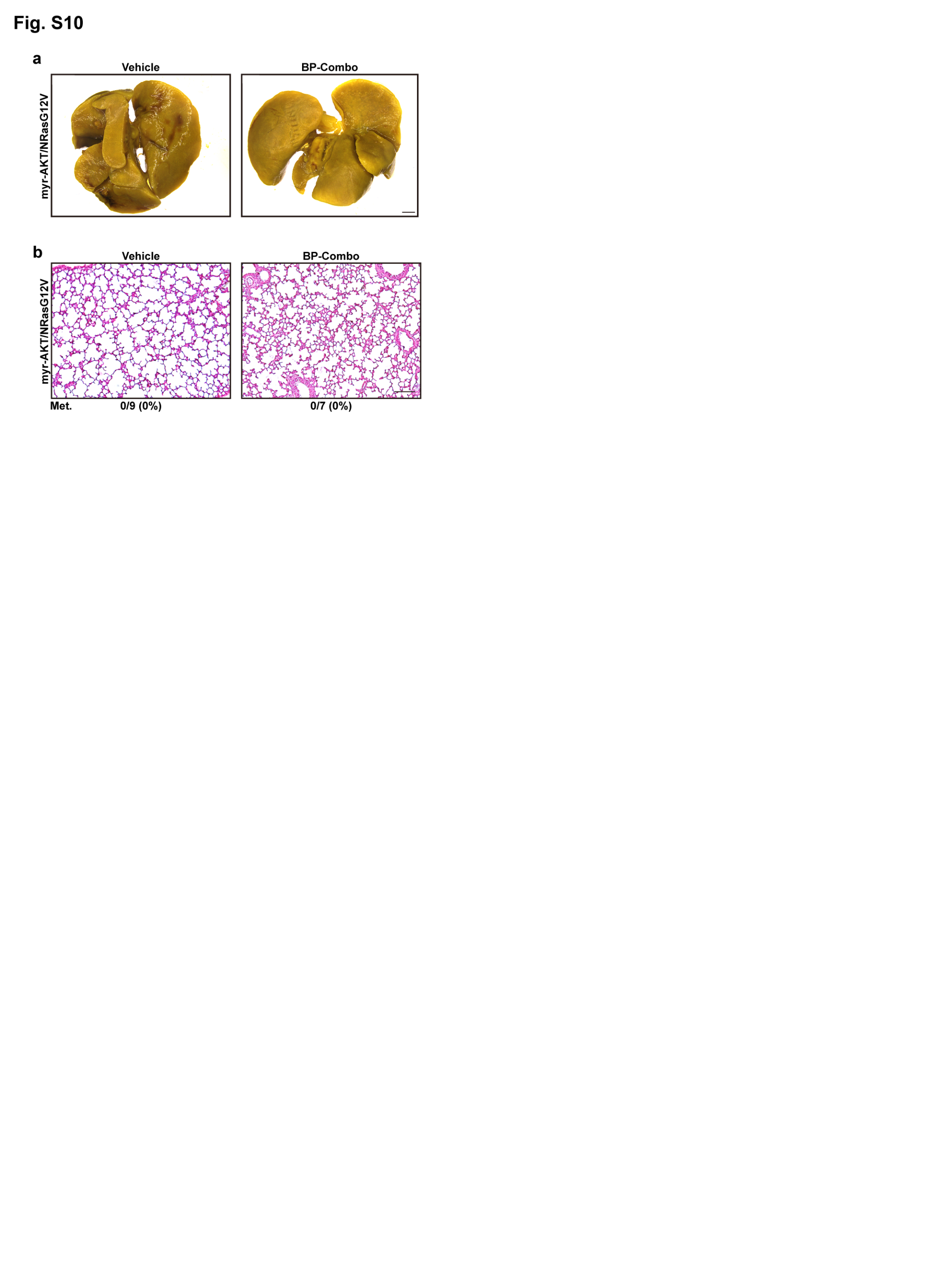
**

**Supplementary Fig. 10. The myr-AKT/NRasG12V mouse model has no pulmonary metastasis.** Representative macroscopic images (**a**) and H&E staining images (**b**) of the lungs are shown. Metastasis rates are indicated under the images (b). Scale bar, 1 mm (a) and 100 μm (b).

**Supplementary Table 1. BTZ and PD0166285 synergistically kill cancer cells**

| **Cell line** | **BTZ^a^**  **(nM)** | | | **PD0166285^a^**  **(nM)** | **Bliss CI^b^** | **BTZ**  **IC_50_ (nM)** | | | **PD0166285**  **IC_50_ (nM)** | |
| --- | --- | --- | --- | --- | --- | --- | --- | --- | --- | --- |
|  |  |  |  |  |  | **Single^c^** | | **Combined^d^** | **Single^c^** | **Combined^d^** |
| SNU449 | | 10 | 200 | | 0.74 | 19 | 8 | | 3612 | 633 |
| Huh1 | | 10 | 100 | | 0.50 | 39 | 18 | | 760 | 258 |
| HepG2 | | 10 | 100 | | 0.49 | 14 | 3 | | 1367 | 83 |
| SK-Hep-1 | | 10 | 100 | | 0.46 | 16 | 6 | | 1270 | 124 |
| Hepa1-6 | | 10 | 200 | | 0.46 | 44 | 2 | | 1959 | 131 |
| HeLa | | 10 | 100 | | 0.21 | 27 | 8 | | 351 | 40 |
| U2OS | | 10 | 100 | | 0.71 | 12 | 6 | | 175 | 61 |
| HBERST | | 10 | 100 | | 0.43 | 60 | 9 | | 821 | 79 |
| HBE | | 10 | 100 | | 1.32 | 1383 |  | | 10015 |  |
|  | | 10 | 200 | | 1.68 | 1383 |  | | 10015 |  |
|  | | 100 | 4000 | | 1.09 | 1383 |  | | 10015 |  |
| 293T | | 10 | 100 | | 0.95 | 553 |  | | 4411 |  |
|  | | 10 | 200 | | 1.13 | 553 |  | | 4411 |  |
|  | | 100 | 4000 | | 1.15 | 553 |  | | 4411 |  |
| L02 | | 10 | 100 | | 0.98 | 457 |  | | 10295 |  |
|  | | 10 | 200 | | 1.64 | 457 |  | | 10295 |  |
|  | | 100 | 4000 | | 1.10 | 457 |  | | 10295 |  |
| LX2 | | 10 | 100 | | 1.52 | 863 |  | | 3263 |  |
|  | | 10 | 200 | | 1.94 | 863 |  | | 3263 |  |
|  | | 100 | 4000 | | 1.15 | 863 |  | | 3263 |  |
| SF | | 10 | 100 | | 1.43 | 265 |  | | 11812 |  |
|  | | 10 | 200 | | 1.83 | 265 |  | | 11812 |  |
|  | | 100 | 4000 | | 1.00 | 265 |  | | 11812 |  |

^a^The drug concentrations for the combined bortezomib (BTZ) and PD0166285 treatment and Bliss combination index (Bliss CI) calculation.

^b^Bliss CI for the combined BTZ and PD0166285 treatment was calculated based on three independent experiments. Bliss CI of less than 1 indicates synergy, a CI of less than 0.75 indicates strong synergy, and a CI of greater than 1 indicates antagonism.

^c^The half maximal inhibitory concentration (IC50) of BTZ or PD0166285 in the monotreatment was calculated based on the average values of three independent experiments.

**^d^**The IC50 of BTZ in the combined treatment with the indicated dose of PD0166285 (in the 3^rd^ column) or the IC50 of PD0166285 in the combined treatment with the indicated dose of BTZ (in the 2^nd^ column) were calculated based on the average values of three independent experiments.

**Supplementary Table 2. Sequences of RNA and DNA oligonucleotides**

| **Name** | **Sense strand/sense primer (5′-3′)** | **Antisense strand/antisense primer (5′-3′)** | |
| --- | --- | --- | --- |
| **siRNA duplexes** | | | |
| NC | UUCUCCGAACGUGUCACGUdTdT | | ACGUGACACGUUCGGAGAAdTdT |
| siPSMC1 | GAACGUAGAAUGAAAGUAAdTdT | | UUACUUUCAUUCUACGUUCdTdC |
| siPSMC2 | GUGCUUCAUUCGAGUUAUUdTdT | | AAUAACUCGAAUGAAGCACdGdC |
| siPSMC3 | CCAUGAAGGACAAGAUAAAdTdT | | UUUAUCUUGUCCUUCAUGGdCdT |
| siPSMC4 | GGGAAGACCAUGUUGGCAAdTdT | | UUGCCAACAUGGUCUUCCCdAdC |
| siPSMC5-1 | AGAAGAAAGUGUUGGUCAAdTdT | | UUGACCAACACUUUCUUCUdTdA |
| siPSMC5-2 | GGAACGAACUAAAUGCUAAdTdT | | UUAGCAUUUAGUUCGUUCCdTdC |
| siPSMC6 | AGAGUUAUUUCAGCGUGUAdTdT | | UACACGCUGAAAUAACUCUdGdG |
| siGSDMB | GGAGACGGUAAAGGAGGAAdTdT | | UUCCUCCUUUACCGUCUCCdAdG |
| siGSDMD | GCAGGAGCUUCCACUUCUAdTdT | | UAGAAGUGGAAGCUCCUGCdCdA |
| siGSDME | GCAGCAAGCAGCUGUUUAUdTdT | | AUAAACAGCUGCUUGCUGCdCdT |
| siCaspase-1 | AGAGUGACUUUGACAAGAUdTdT | | AUCUUGUCAAAGUCACUCUdTdT |
| siCaspase-3 | GCAGCAAACCUCAGGGAAAdTdT | | UUUCCCUGAGGUUUGCUGCdAdT |
| siRpn10 | CCAAGGAGGAGGAUGAUUAdTdT | | UAAUCAUCCUCCUCCUUGGdCdT |
| siRpn11 | GGACAUGAACCAAGACAAAdTdT | | UUUGUCUUGGUUCAUGUCCdTdA |
| siRpn13 | GGAAAGGGCUGGUGUACAUdTdT | | AUGUACACCAGCCCUUUCCdGdC |
| sicGAS | GGAAGAAAUUAACGACAUUdTdT | | AAUGUCGUUAAUUUCUUCCdTdT |
| siCHOP | GGAAAUGAAGAGGAAGAAUdTdT | | AUUCUUCCUCUUCAUUUCCdTdT |
| siIκBα | AGUCAGAGUUCACGGAGUUdTdT | | AACUCCGUGAACUCUGACUdCdT |
| **Primers for cloning** | | | |
| Cloning into pCDH | | | |
| H2B | TGCTCTAGAATGCCTGAACCCTCTAAGTC | | TCCTCGCCCTTGCTCACTTTAGAGCTAGTGTACTTGG |
| EGFP | CAAGTACACTAGCTCTAAAGTGAGCAAGGGCGAGGAGC | | CGCGGATCCTCACTTGTACAGCTCGTCC |
| BCL-xL | CCGGAATTCGCCACCATGTCTCAGAGCAACCGGGAG | | ATTTGCGGCCGCTCATTTCCGACTGAAGAGTGAGC |
| CDK1 (WT) | CCGGAATTCGCCACCATGGAAGATTATACCAAAATAGAGAA | | CGCGGATCCCTACATCTTCTTAATCTGATTGTCCA |
| CDK1 (T14A/Y15F) | CCGGAATTCGCCACCATGGAAGATTATACCAAAATAGAGAAAATTGGAGAAGGTGCCTTTGGAGTTGTGTATAAGG | | CGCGGATCCCTACATCTTCTTAATCTGATTGTCCA |
| **Primers for qPCR** | | | |
| \| PSMC1 \| AACCTCCTGTACCAACTAG \| TGGTTTCATTTGTTCCTGAT \| \| --- \| --- \| --- \| \| PSMC2 \| GATATTGCCTTGTTGAA \| GTAAAGGCTGTTCACTCT \| \| PSMC3 \| ACTCCTATCTGATCCTGGA \| CAAGTTCTCAAACTTCTC \| \| PSMC4 \| AAGGAATTTCTCCATGC \| CGATCGATGGTGCTCAG \| \| PSMC5 \| GTGATCGAGCTGCCTGTTAAGC \| CAGTCCGTATGATGAGCCACAG \| \| PSMC6 \| GCTTCTCATCATGGCGGA \| GTAGGGCCTTCAGATCAT \| \| Rpn10 \| GACGATGCCCTGCTGAAGAT \| CTTGGCTGGCTCAGATGTGT \| \| Rpn11 \| CTGATTCATCGCCGGTTTGC \| CTGGCAGGTACAACTTCCCC \| \| Rpn13 \| TGCTGACGCCGGAGATAATG \| CAGACCGAACTGGCACATGA \| \| GAPDH \| GAGTCAACGGATTTGGTCGT \| GACAAGCTTCCCGTTCTCAG \| \| GSDMA \| TACGTCCGCACCGACTACA \| CAGAGTGCTGTTCTGCGAGA \| \| GSDMB \| ATGTAGACTCAACGGGAGAGTT \| GTAGCCAGATACTGCTGGGATA \| \| GSDMC \| TCCATGTTGGAACGCATTAGC \| CAAACTGACGTAATTTGGTGGC \| \| GSDMD \| GAGTGTGGCCTAGAGCTGG \| GGCTCAGTCCTGATAGCAGTG \| \| GSDME \| ACATGCAGGTCGAGGAGAAGT \| TCAATGACACCGTAGGCAATG \| \| Caspase-1 \| AGTCACACAAGAAGGGAGGAG \| TCCTTGTTCAGCACCCTTGTC \| \| Caspase-3 \| GTGCTATTGTGAGGCGGTTG \| TCACGGCCTGGGATTTCAAG \| \| cGAS \| GGGCGGTTTTGGAGAAGTTG \| CGTGCTCATAGTAGCTCCCG \| \| CHOP \| GCAGATGTGCTTTTCCAGACT \| GCAGGGTCAAGAGTGGTGAA \| \| IκBα \| AAGTGATCCGCCAGGTGAAG \| CTGCTCACAGGCAAGGTGTA \| \| **Target sequences for shRNA vectors** \| \| \| \| shNC \| TTGTACTACACAAAAGTACTG \| CAGTACTTTTGTGTAGTACAA \| \| shGsdme \| GCTGAACGTTGGTGGCAAA \| TTTGCCACCAACGTTCAGC \| | | | |
