# Supplementary material for: Synergistic induction of mitotic pyroptosis and tumor remission by inhibiting proteasome and WEE family kinases
Source: Signal Transduct Target Ther. 2024 Jul 12;9:181. doi: 10.1038/s41392-024-01896-z (PMC11239683; doi:10.1038/s41392-024-01896-z)
Supplement: Supplementary file 2 — Original data [file 41392_2024_1896_MOESM2_ESM.pptx]

## Slide 1
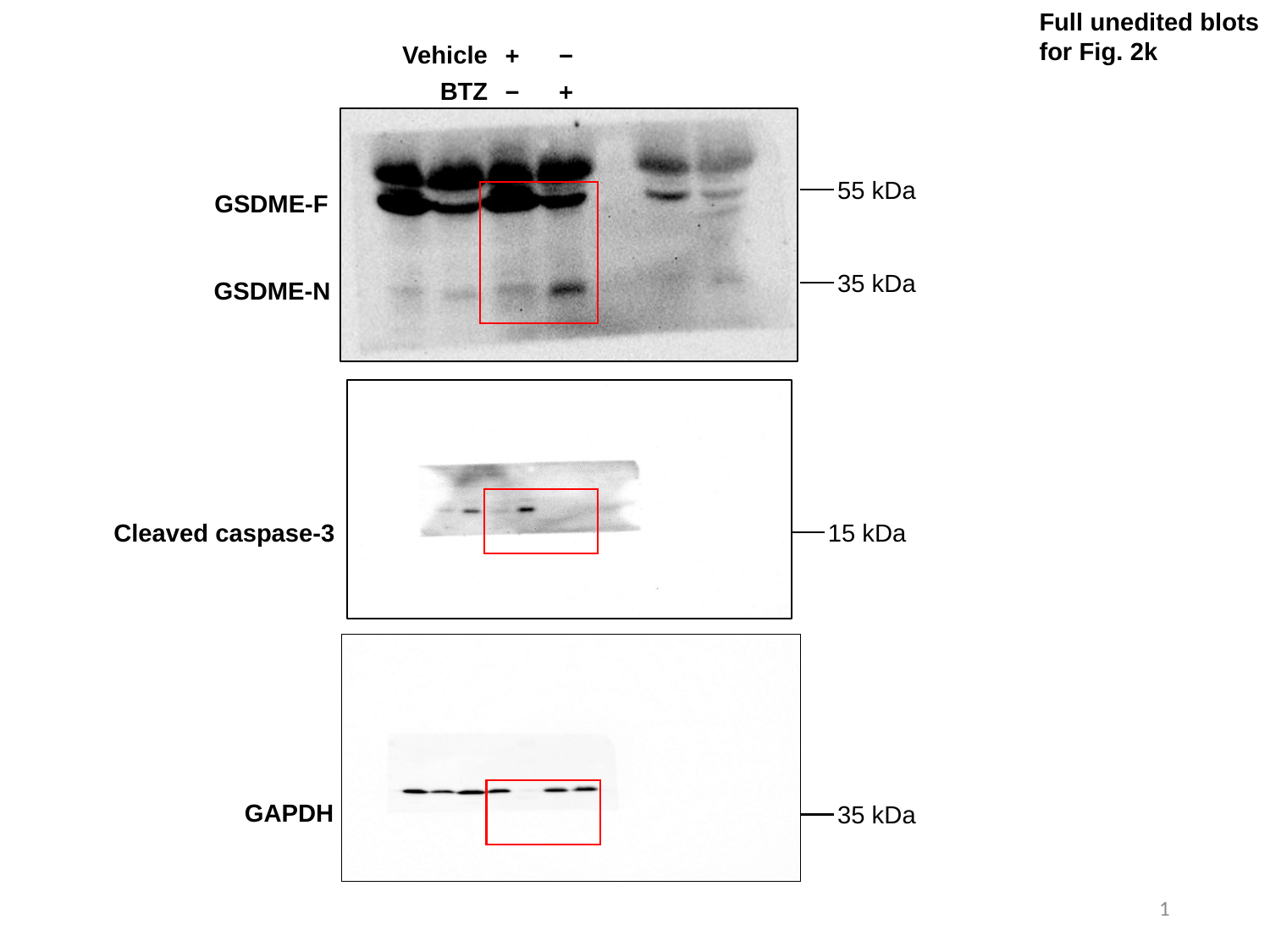

Full unedited blots
for Fig. 2k
Vehicle
+
−
BTZ
−
+
55 kDa
GSDME-F
35 kDa
GSDME-N
Cleaved caspase-3
15 kDa
GAPDH
35 kDa
1

## Slide 2
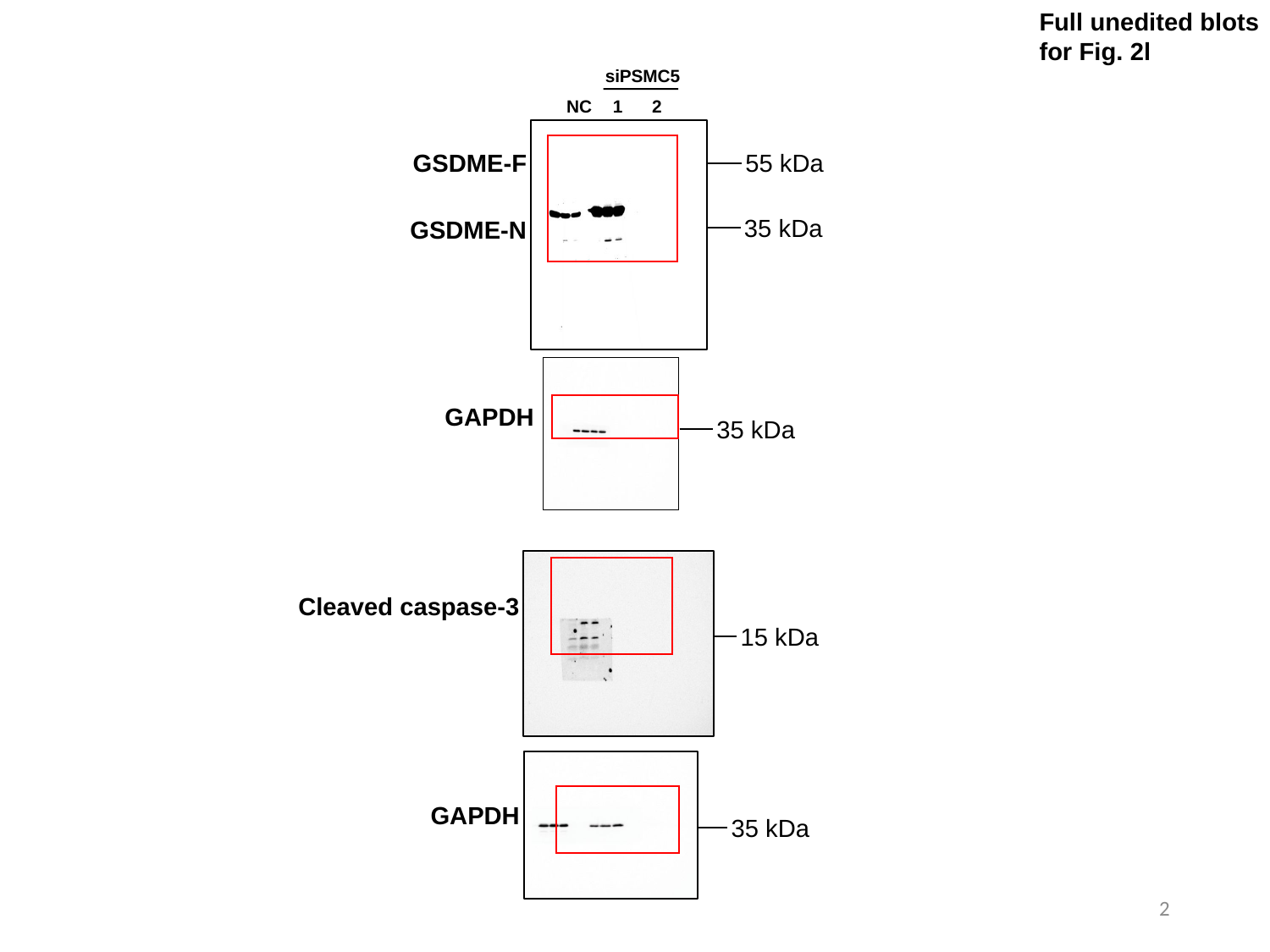

Full unedited blots
for Fig. 2l
siPSMC5
NC
1
2
GSDME-F
55 kDa
35 kDa
GSDME-N
GAPDH
35 kDa
Cleaved caspase-3
15 kDa
GAPDH
35 kDa
2

## Slide 3
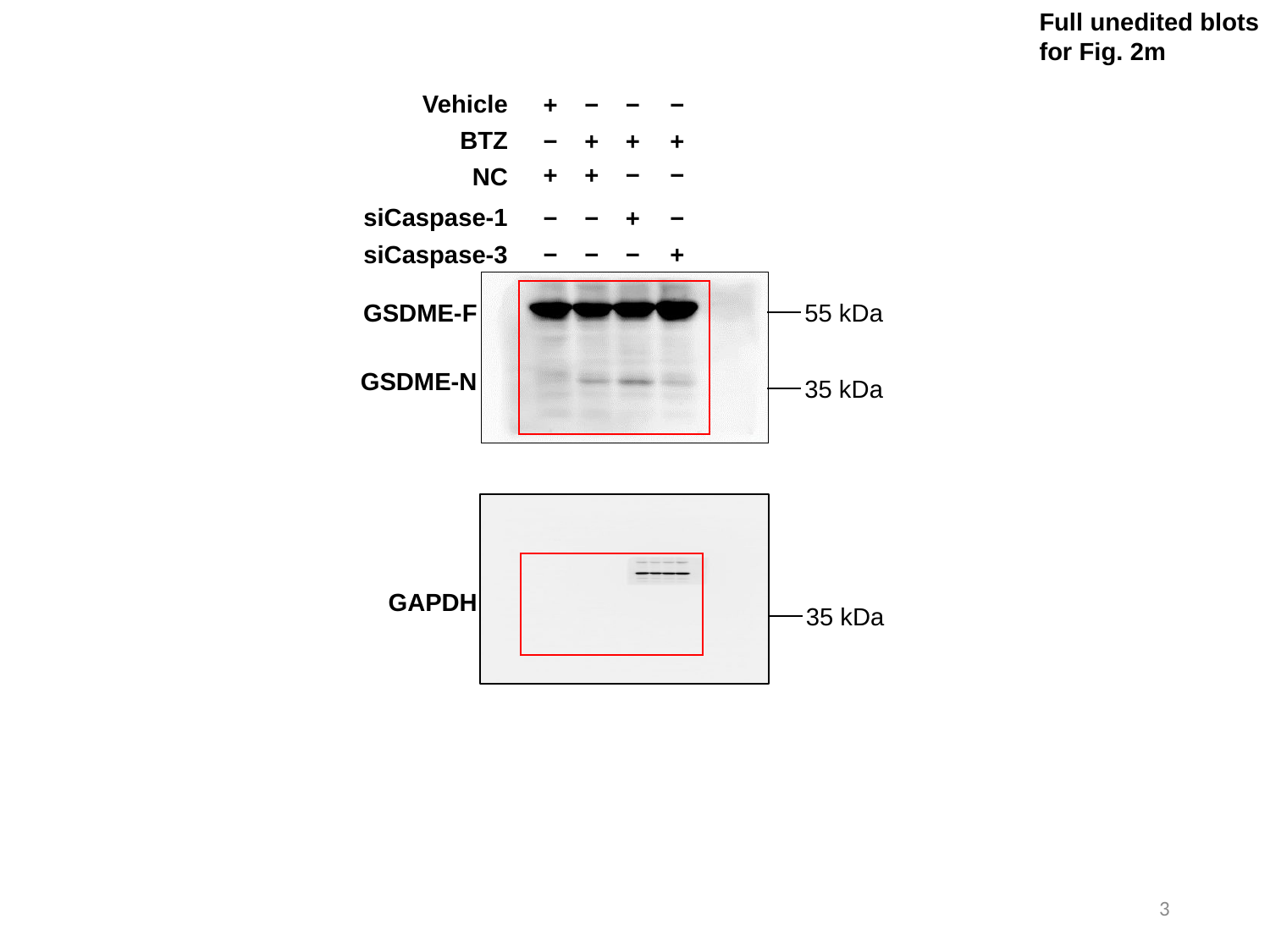

Full unedited blots
for Fig. 2m
Vehicle
+
−
−
−
BTZ
−
+
+
+
+
+
−
−
NC
siCaspase-1
−
−
+
−
siCaspase-3
−
−
−
+
GSDME-F
55 kDa
GSDME-N
35 kDa
GAPDH
35 kDa
3

## Slide 4
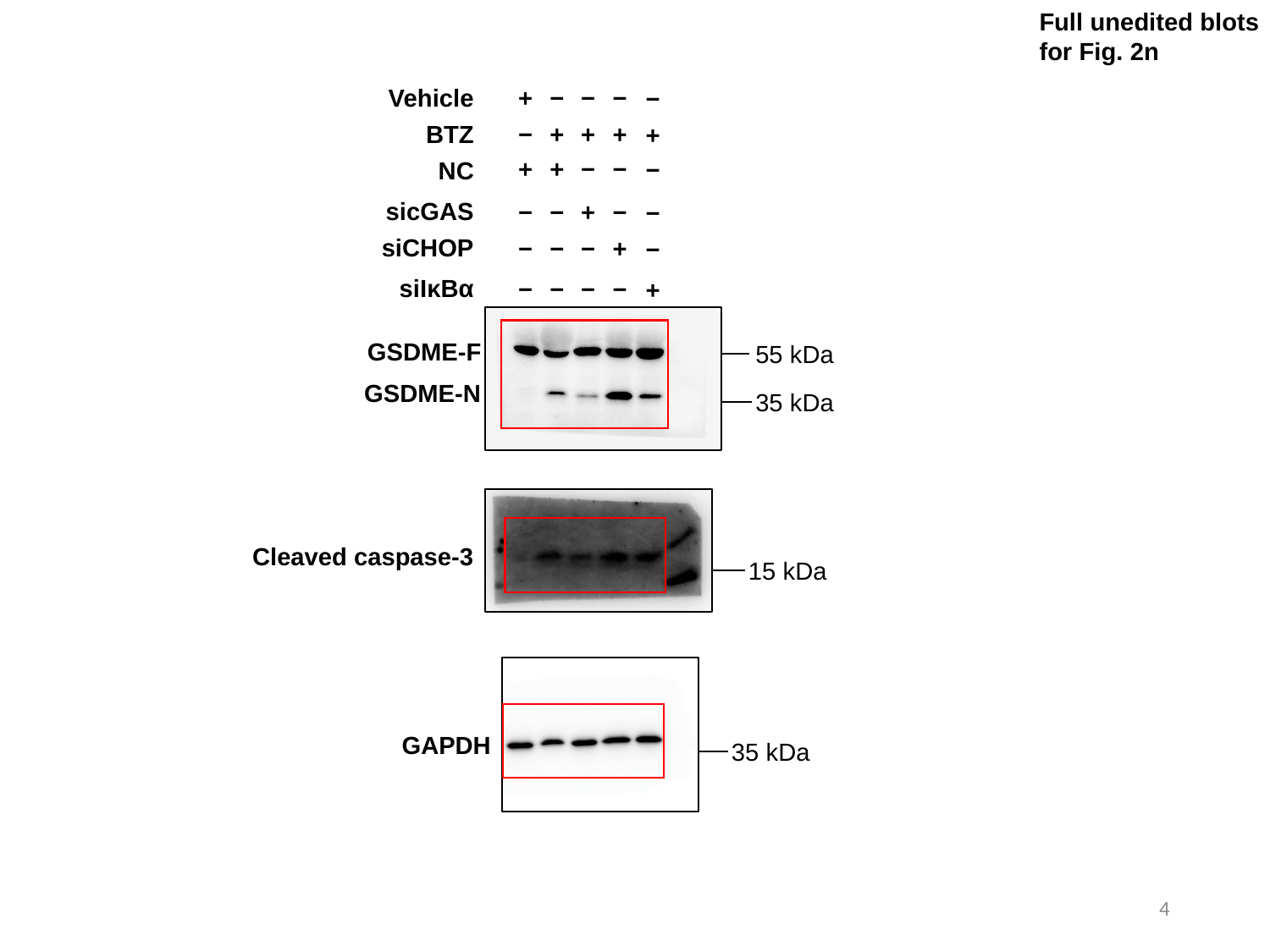

Full unedited blots
for Fig. 2n
Vehicle
+
−
−
−
−
BTZ
−
+
+
+
+
+
+
−
−
−
NC
sicGAS
−
−
+
−
−
siCHOP
−
−
−
+
−
siIκBα
−
−
−
−
+
GSDME-F
55 kDa
GSDME-N
35 kDa
Cleaved caspase-3
15 kDa
GAPDH
35 kDa
4

## Slide 5
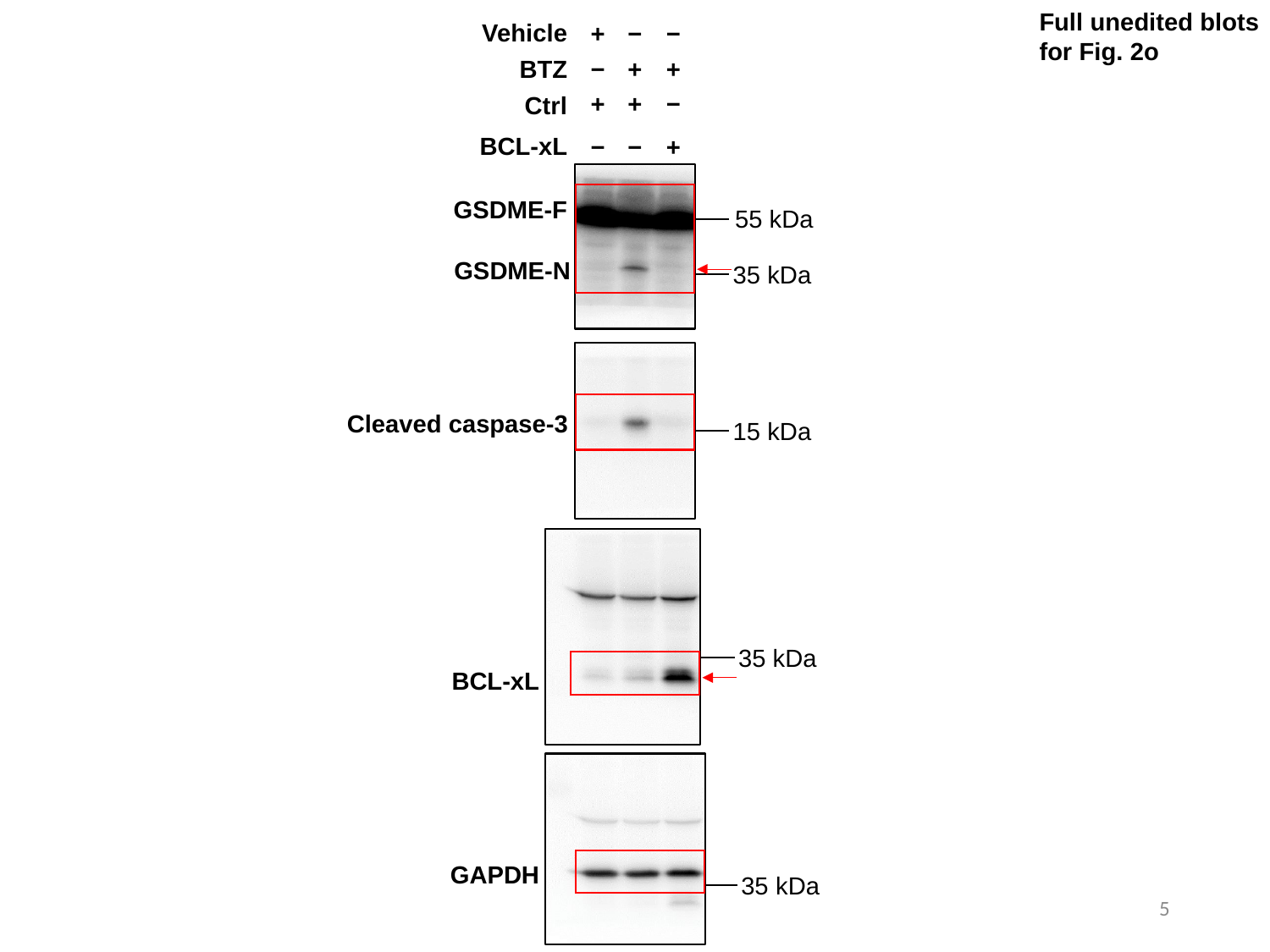

Full unedited blots
for Fig. 2o
Vehicle
+
−
−
BTZ
−
+
+
+
+
−
Ctrl
BCL-xL
−
−
+
GSDME-F
55 kDa
GSDME-N
35 kDa
Cleaved caspase-3
15 kDa
35 kDa
BCL-xL
GAPDH
35 kDa
5

## Slide 6
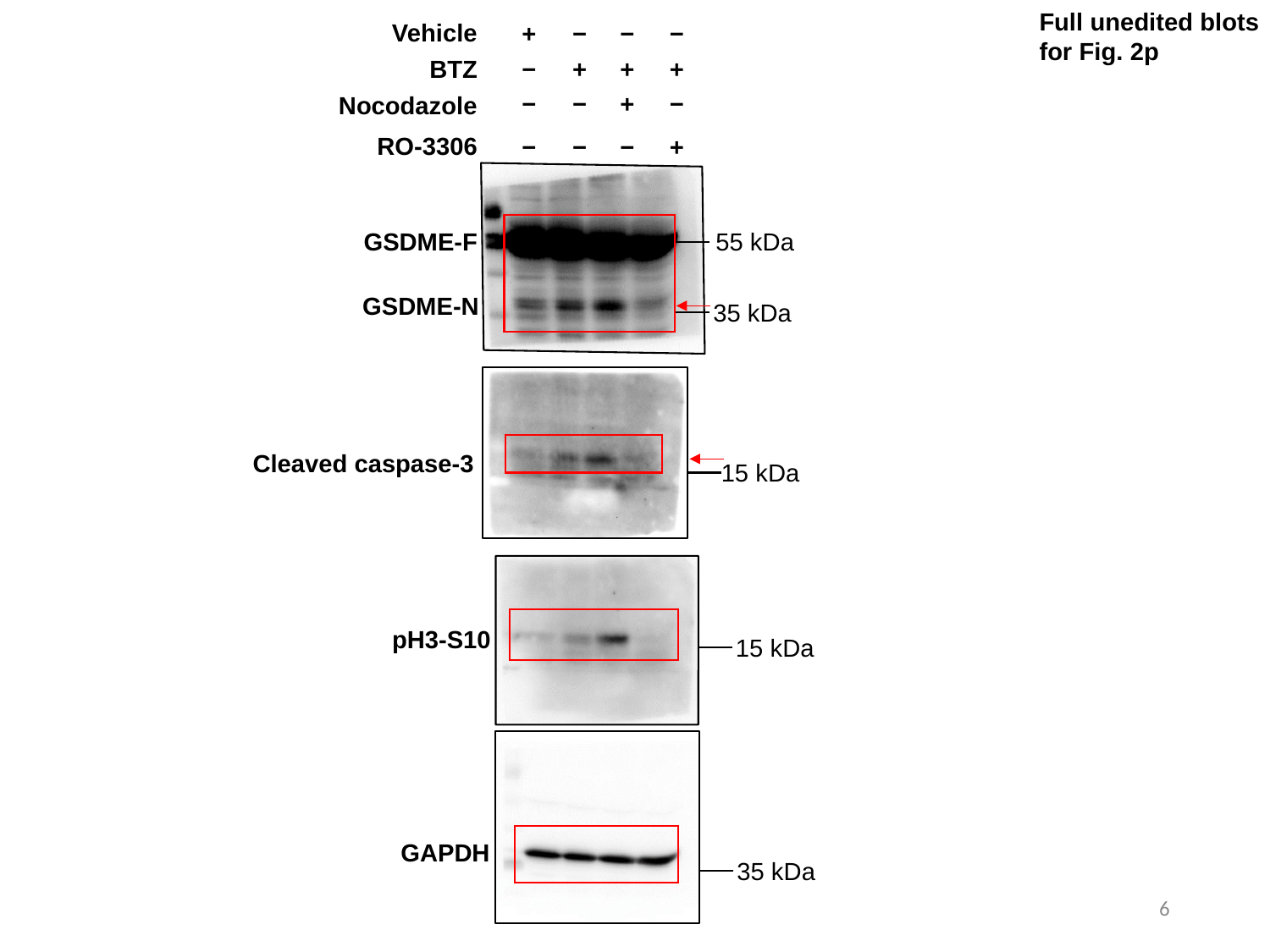

Full unedited blots
for Fig. 2p
Vehicle
+
−
−
−
BTZ
−
+
+
+
−
−
+
−
Nocodazole
RO-3306
−
−
−
+
GSDME-F
55 kDa
GSDME-N
35 kDa
Cleaved caspase-3
15 kDa
pH3-S10
15 kDa
GAPDH
35 kDa
6

## Slide 7
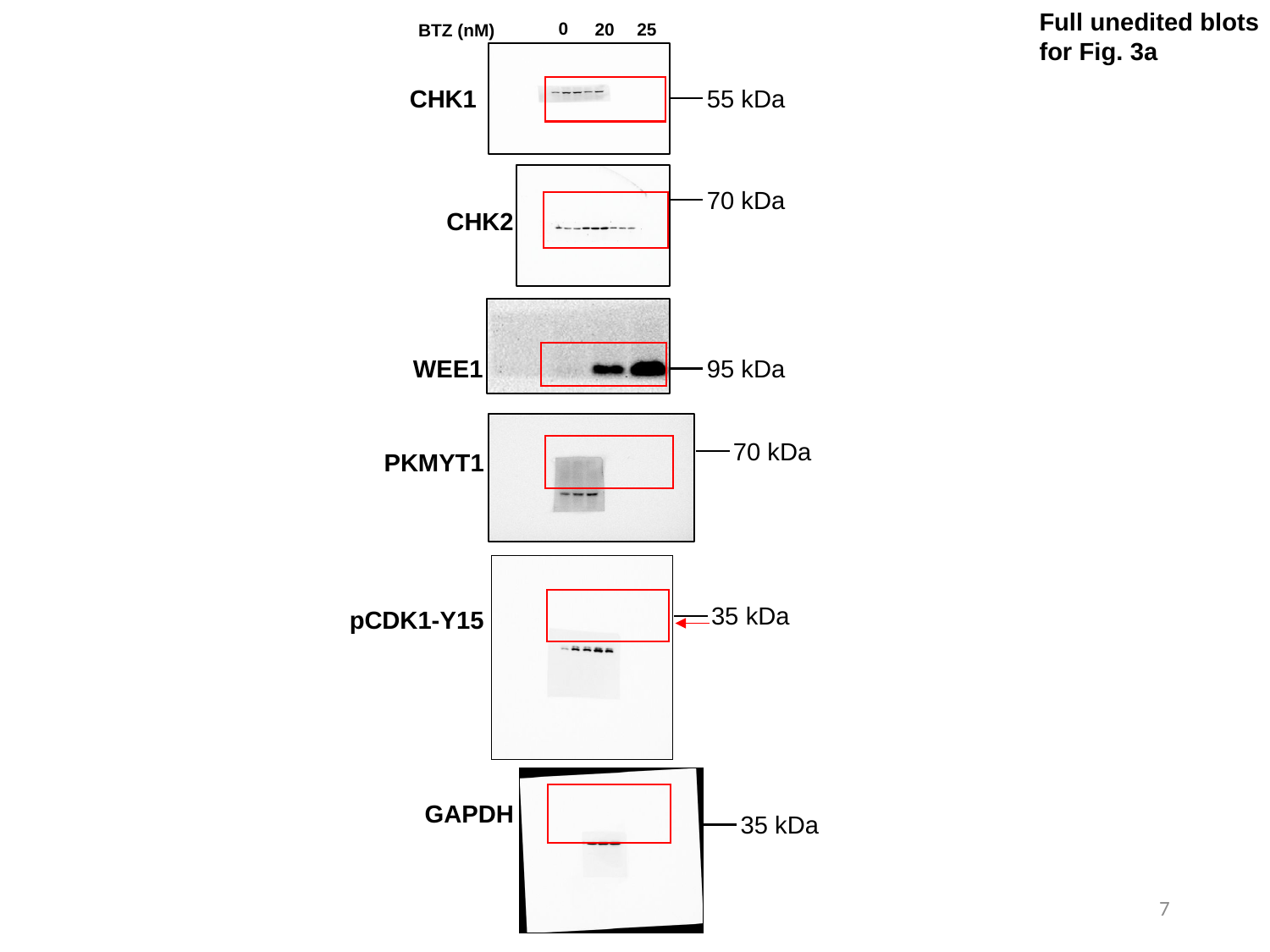

Full unedited blots
for Fig. 3a
0
20
25
BTZ (nM)
55 kDa
CHK1
70 kDa
CHK2
WEE1
95 kDa
70 kDa
PKMYT1
35 kDa
pCDK1-Y15
GAPDH
35 kDa
7

## Slide 8
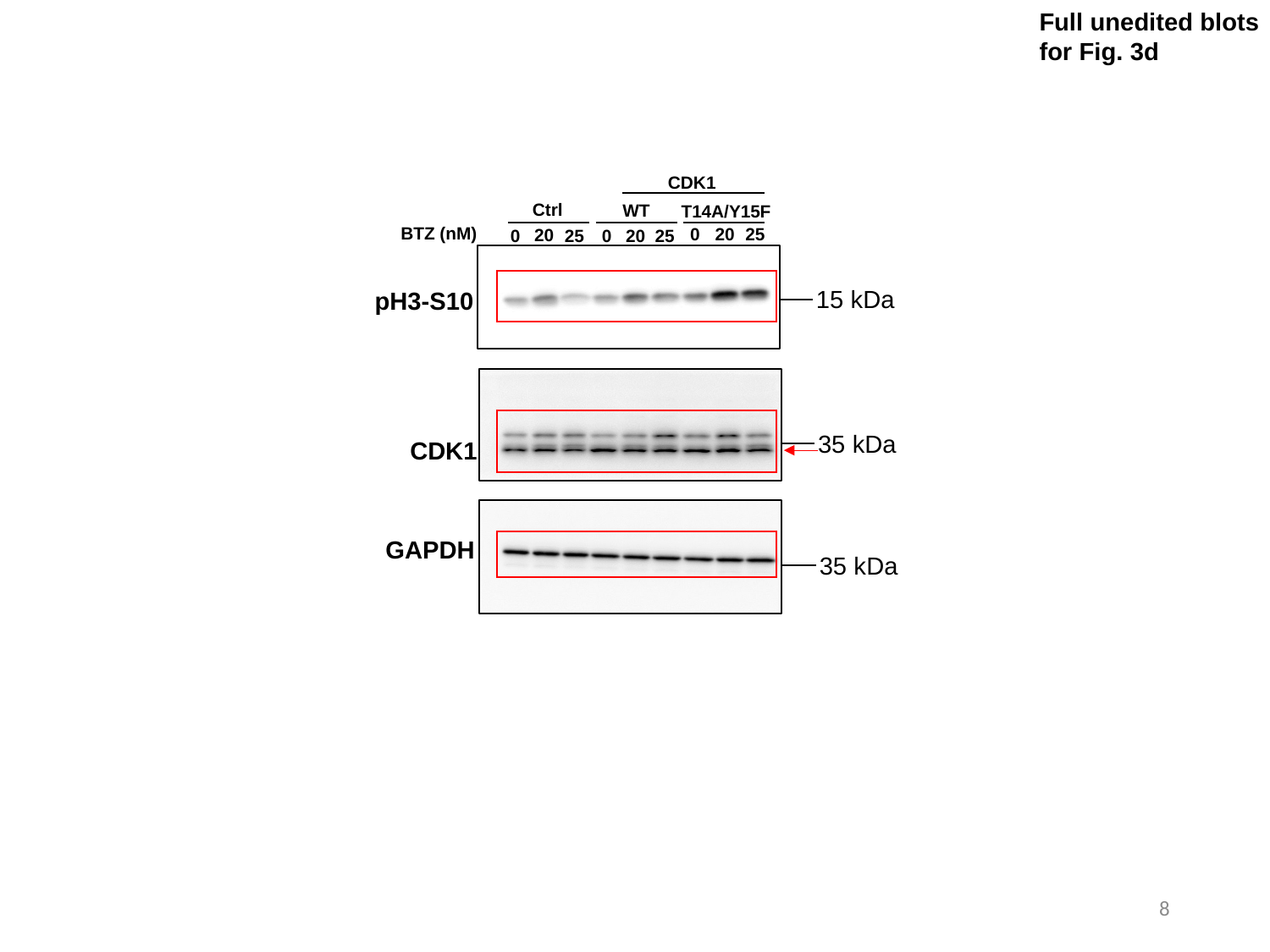

Full unedited blots
for Fig. 3d
CDK1
Ctrl
WT
T14A/Y15F
BTZ (nM)
20
25
0
20
25
0
20
25
0
15 kDa
pH3-S10
35 kDa
CDK1
GAPDH
35 kDa
8

## Slide 9
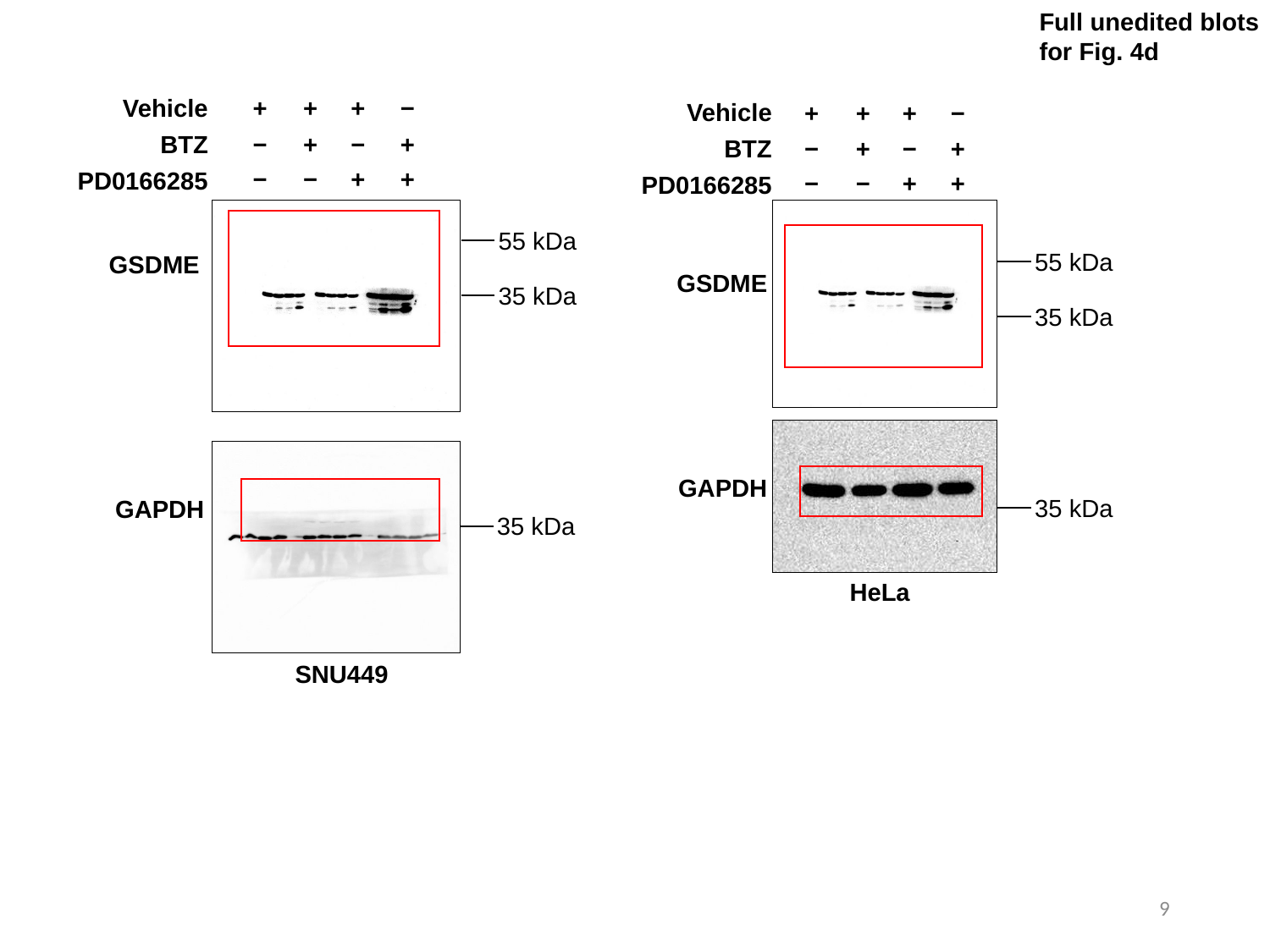

Full unedited blots
for Fig. 4d
Vehicle
+
+
+
−
Vehicle
+
+
+
−
BTZ
−
+
−
+
BTZ
−
+
−
+
−
−
+
+
PD0166285
−
−
+
+
PD0166285
55 kDa
55 kDa
GSDME
GSDME
35 kDa
35 kDa
GAPDH
35 kDa
GAPDH
35 kDa
HeLa
SNU449
9

## Slide 10
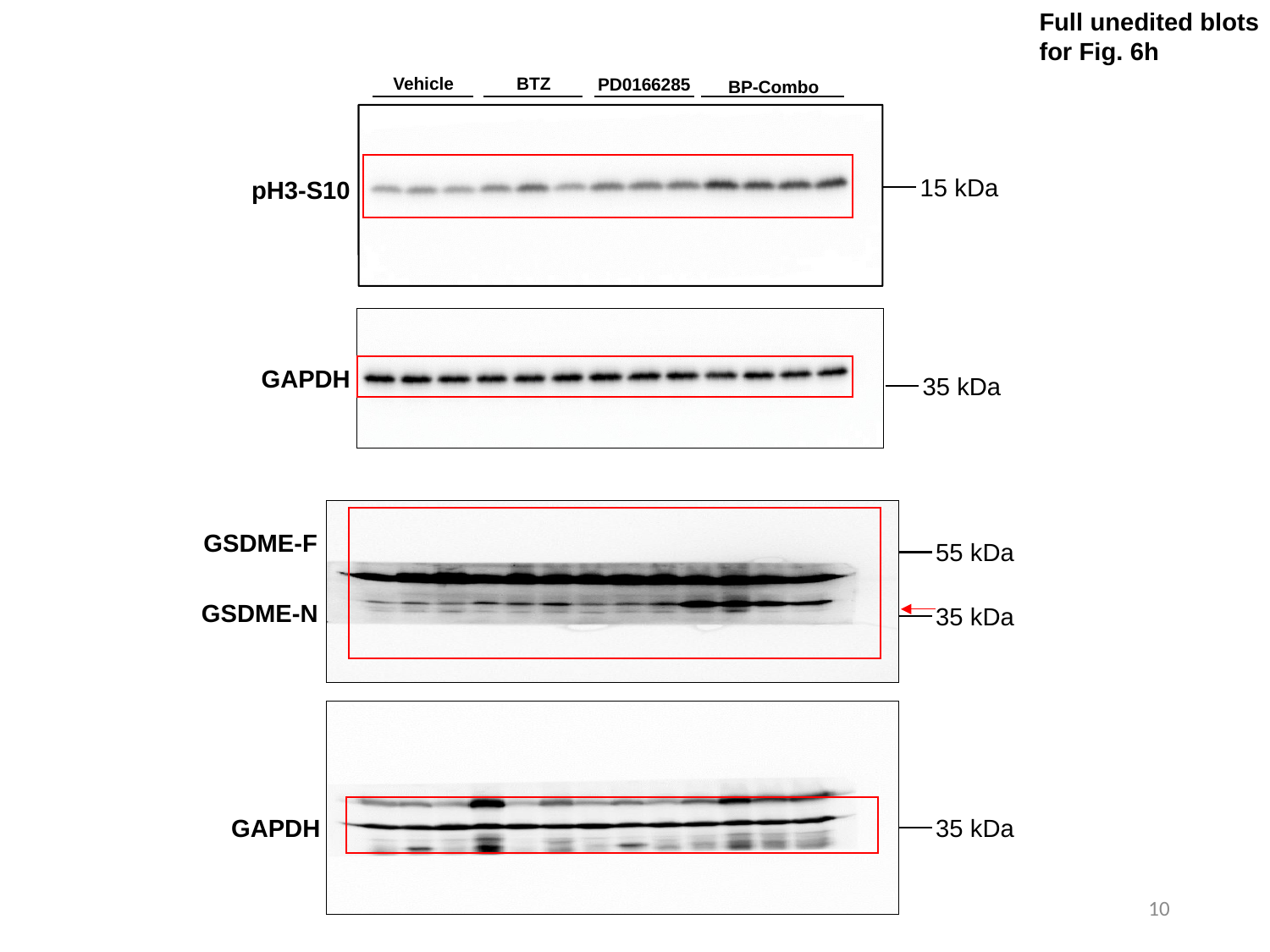

Full unedited blots
for Fig. 6h
Vehicle
BTZ
PD0166285
BP-Combo
15 kDa
pH3-S10
GAPDH
35 kDa
GSDME-F
55 kDa
GSDME-N
35 kDa
GAPDH
35 kDa
10

## Slide 11
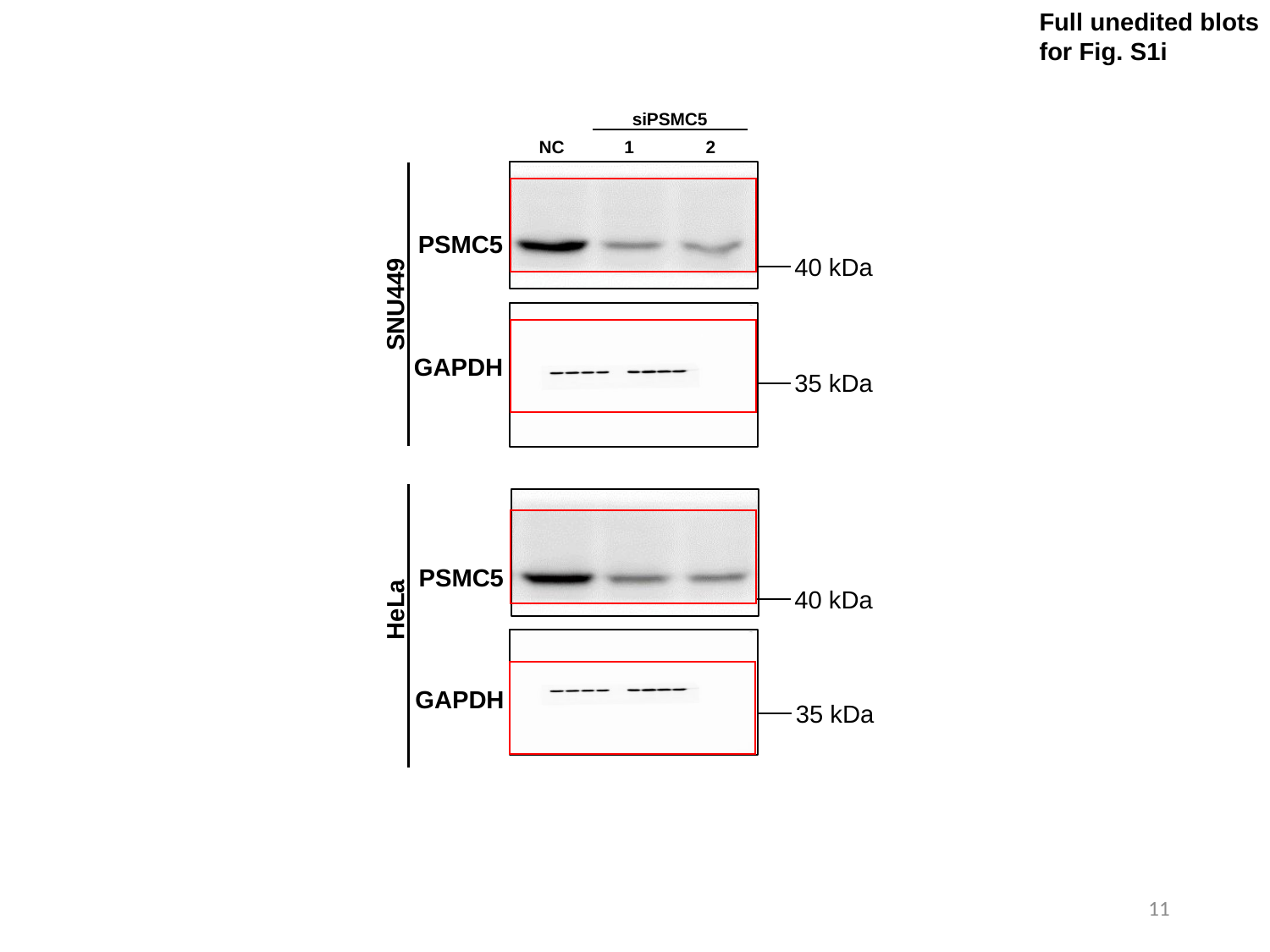

Full unedited blots
for Fig. S1i
siPSMC5
NC
1
2
PSMC5
40 kDa
SNU449
GAPDH
35 kDa
PSMC5
40 kDa
HeLa
GAPDH
35 kDa
11

## Slide 12
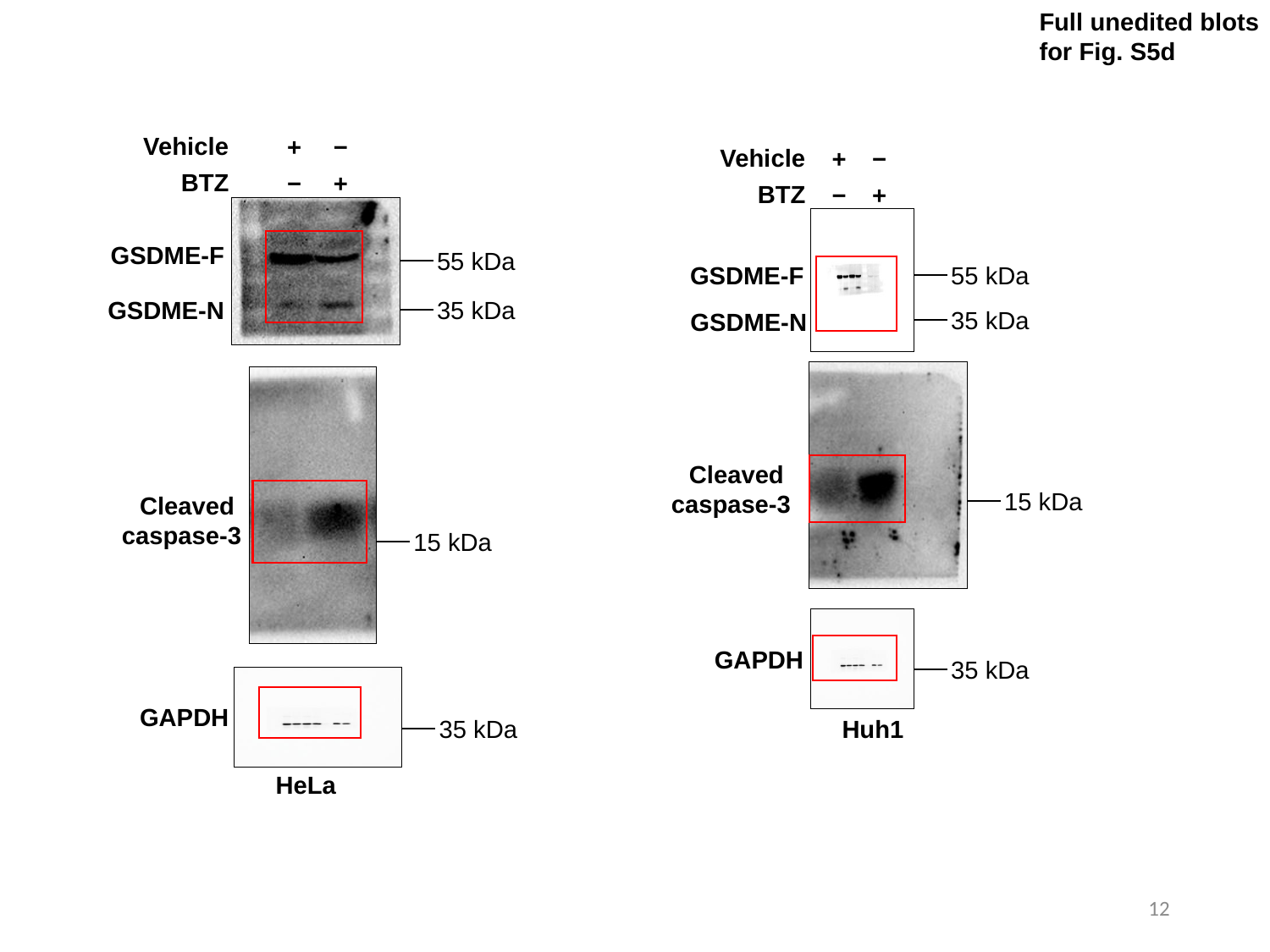

Full unedited blots
for Fig. S5d
Vehicle
+
−
Vehicle
+
−
BTZ
−
+
BTZ
−
+
GSDME-F
55 kDa
35 kDa
GSDME-F
55 kDa
GSDME-N
35 kDa
GSDME-N
Cleaved
caspase-3
15 kDa
Cleaved
caspase-3
15 kDa
GAPDH
35 kDa
GAPDH
Huh1
35 kDa
HeLa
12

## Slide 13
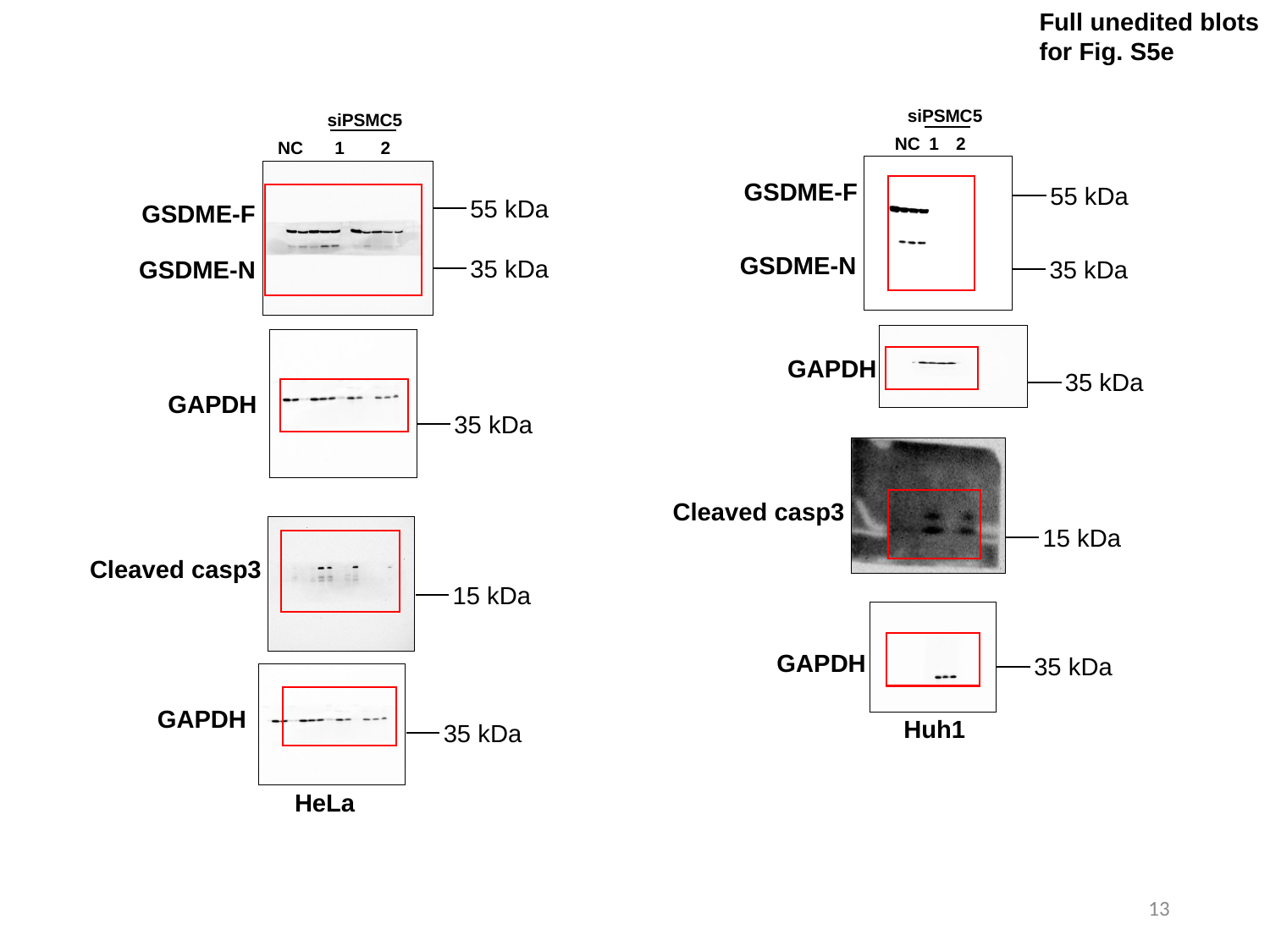

Full unedited blots
for Fig. S5e
siPSMC5
siPSMC5
NC
1
2
NC
1
2
GSDME-F
55 kDa
55 kDa
GSDME-F
GSDME-N
35 kDa
GSDME-N
35 kDa
GAPDH
35 kDa
GAPDH
35 kDa
Cleaved casp3
15 kDa
Cleaved casp3
15 kDa
GAPDH
35 kDa
GAPDH
Huh1
35 kDa
HeLa
13

## Slide 14
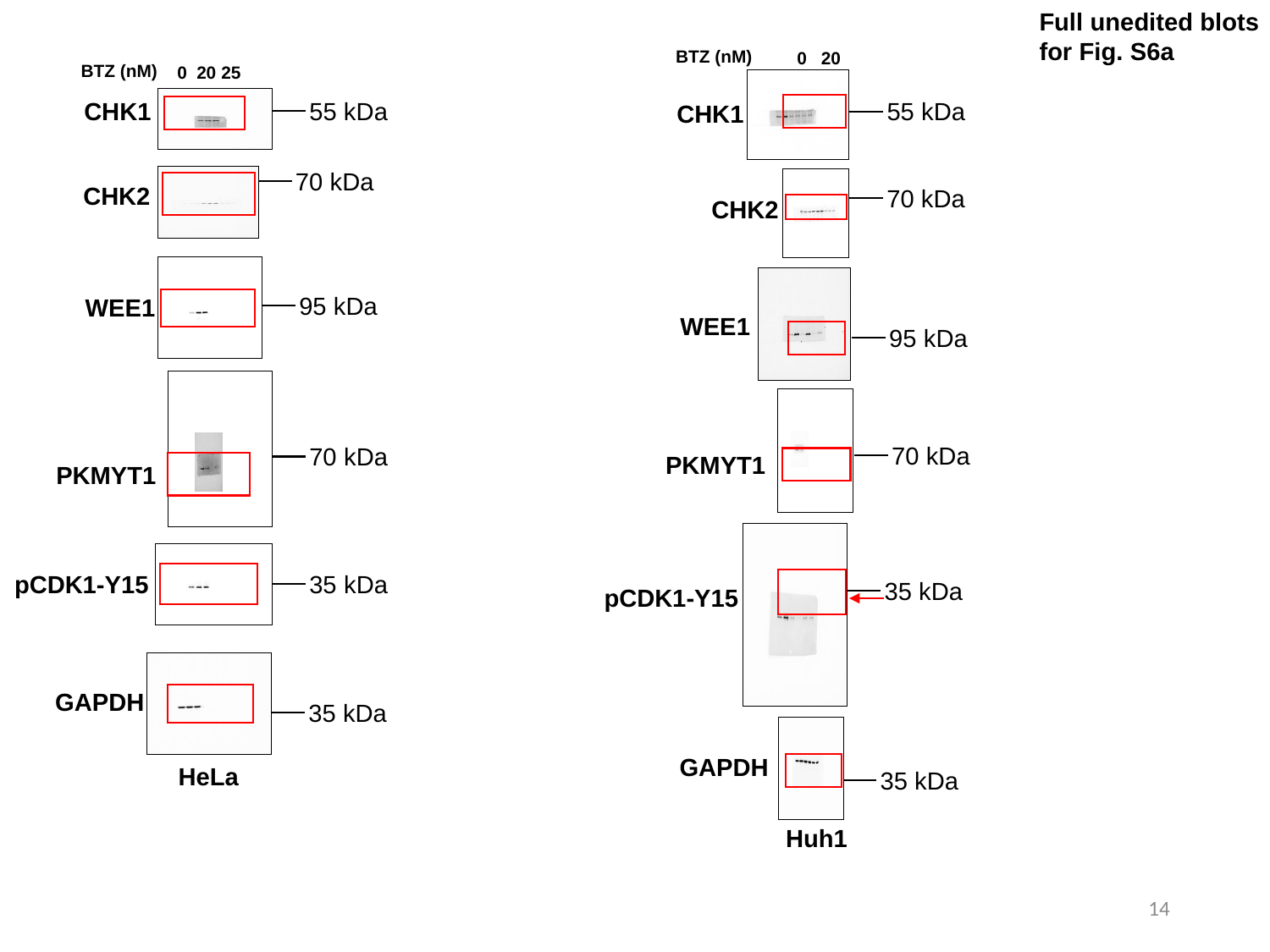

Full unedited blots
for Fig. S6a
BTZ (nM)
20
0
BTZ (nM)
20
25
0
55 kDa
CHK1
55 kDa
CHK1
70 kDa
CHK2
70 kDa
CHK2
95 kDa
WEE1
WEE1
95 kDa
70 kDa
70 kDa
PKMYT1
PKMYT1
35 kDa
pCDK1-Y15
35 kDa
pCDK1-Y15
GAPDH
35 kDa
GAPDH
HeLa
35 kDa
Huh1
14

## Slide 15
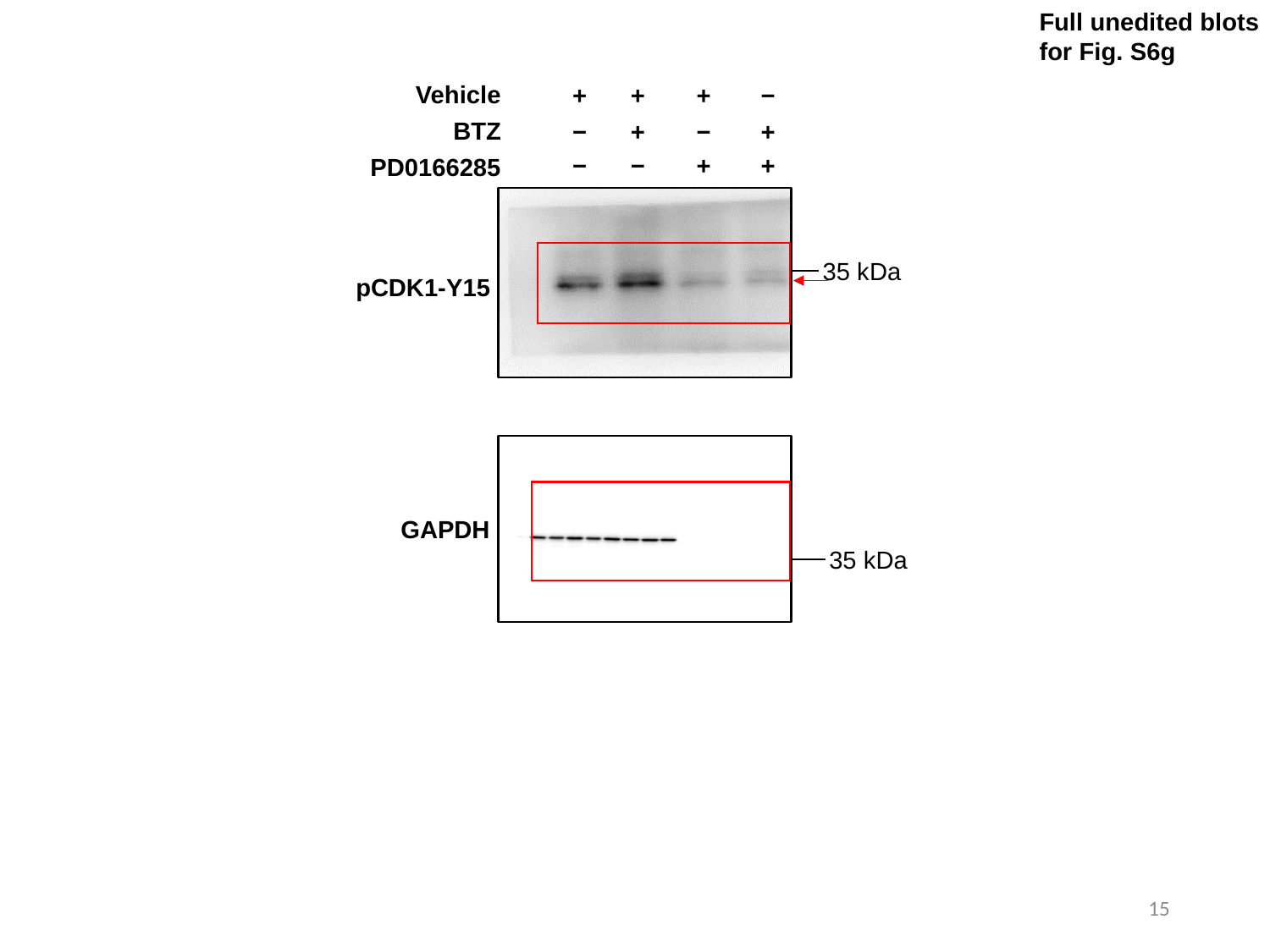

Full unedited blots
for Fig. S6g
Vehicle
+
+
+
−
BTZ
−
+
−
+
−
−
+
+
PD0166285
35 kDa
pCDK1-Y15
GAPDH
35 kDa
15

## Slide 16
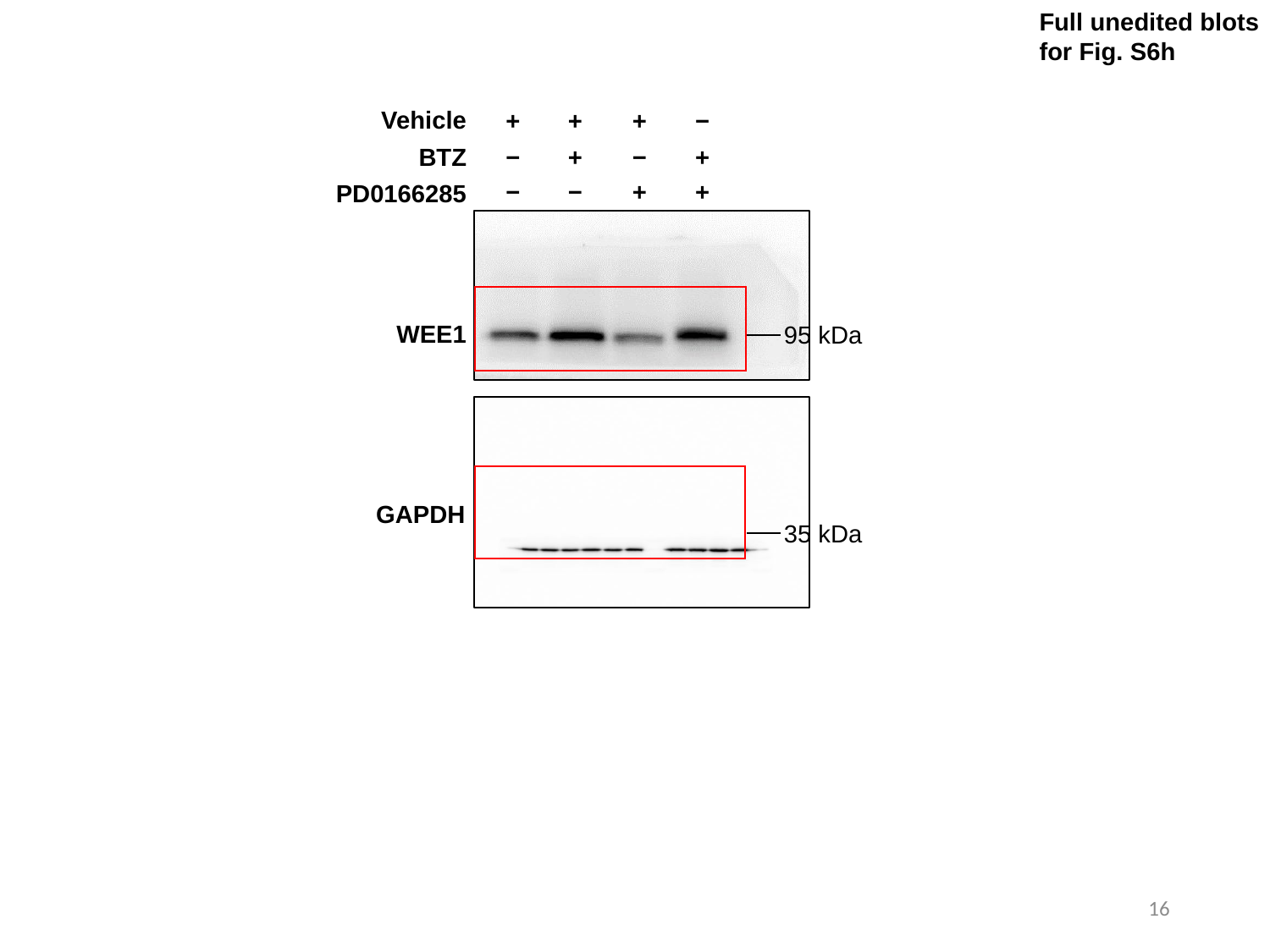

Full unedited blots
for Fig. S6h
Vehicle
+
+
+
−
BTZ
−
+
−
+
−
−
+
+
PD0166285
WEE1
95 kDa
GAPDH
35 kDa
16

## Slide 17
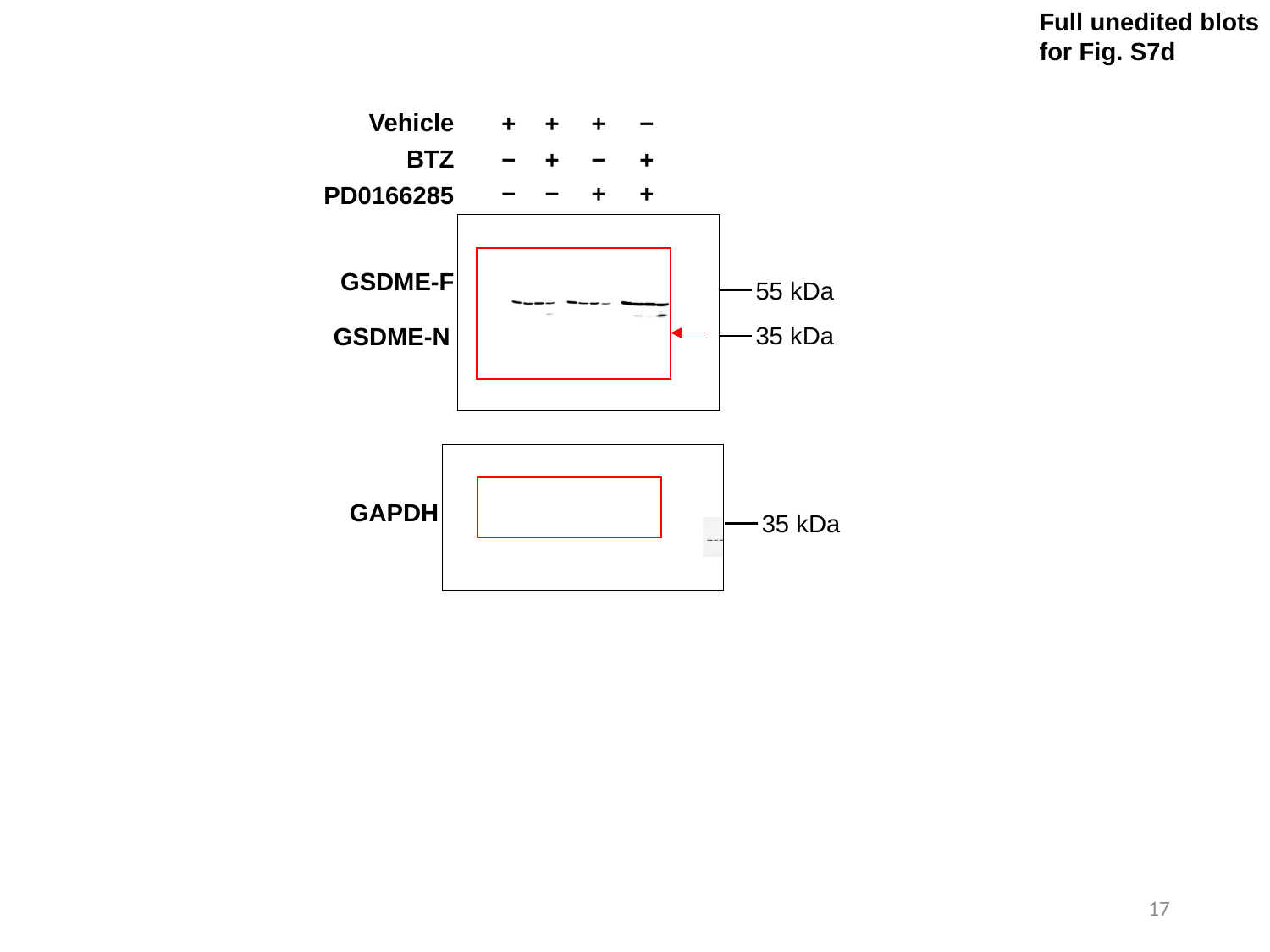

Full unedited blots
for Fig. S7d
Vehicle
+
+
+
−
BTZ
−
+
−
+
−
−
+
+
PD0166285
GSDME-F
55 kDa
35 kDa
GSDME-N
GAPDH
35 kDa
17

## Slide 18
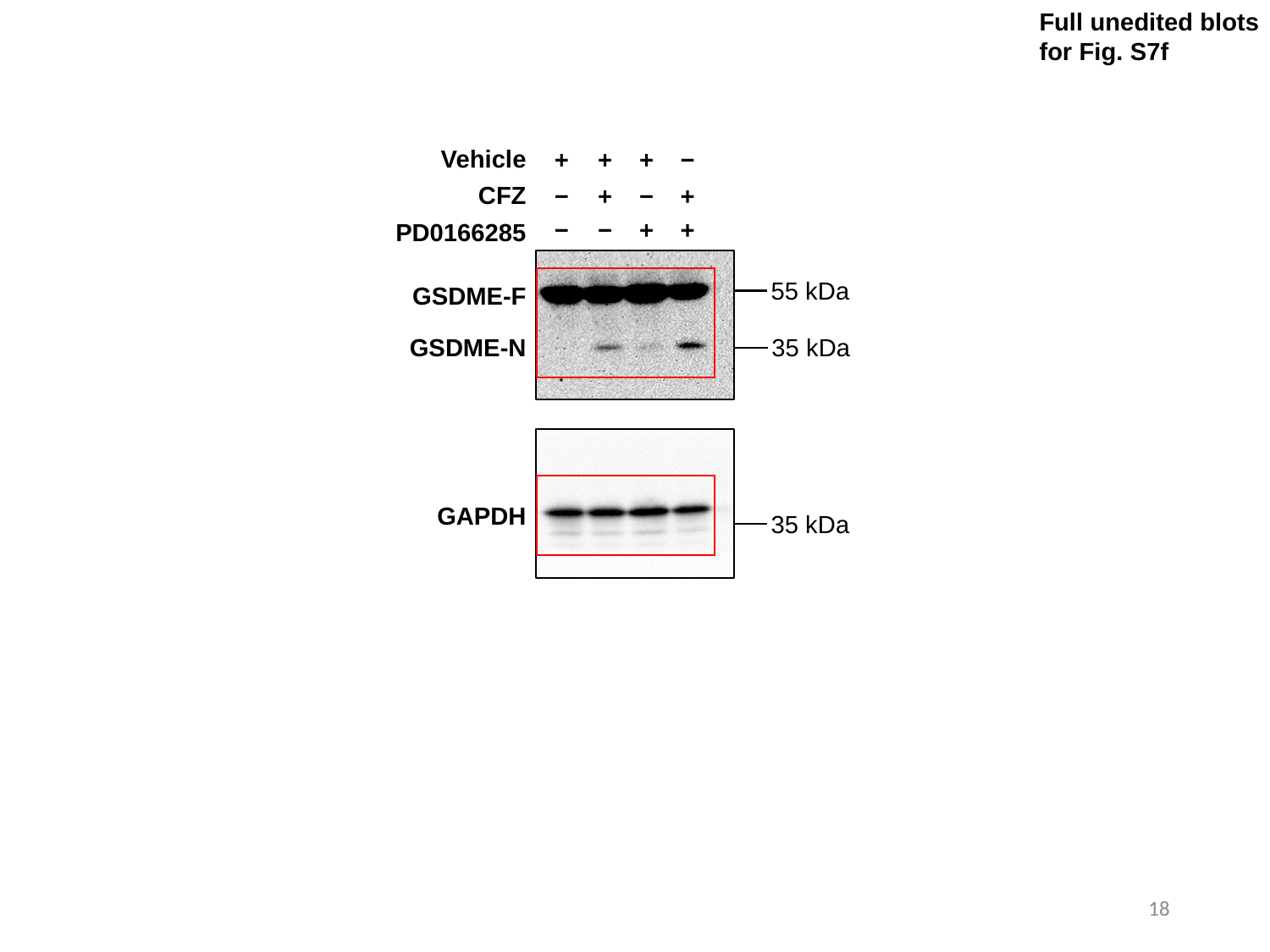

Full unedited blots
for Fig. S7f
Vehicle
+
+
+
−
CFZ
−
+
−
+
−
−
+
+
PD0166285
55 kDa
GSDME-F
GSDME-N
35 kDa
GAPDH
35 kDa
18

## Slide 19
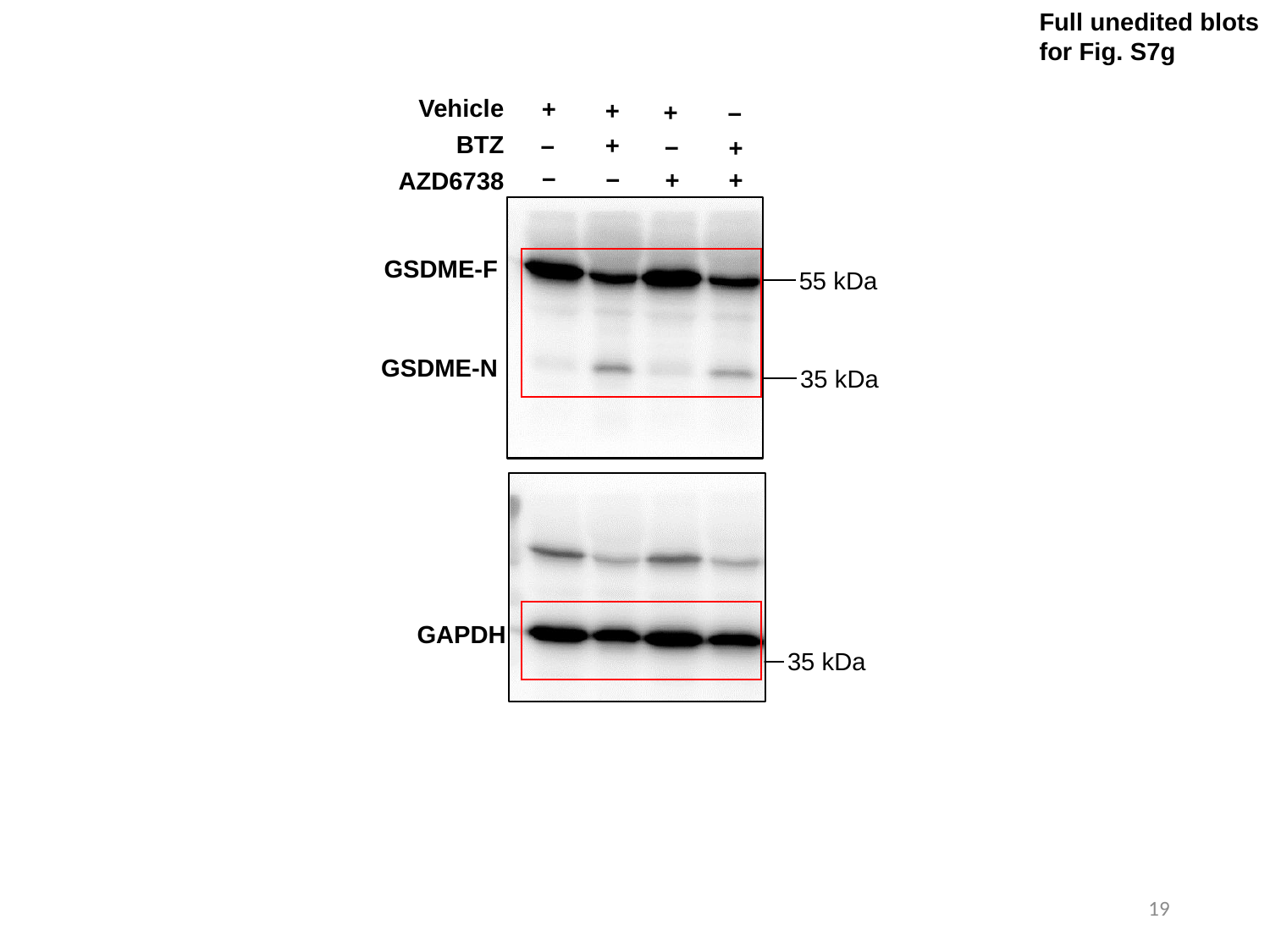

Full unedited blots
for Fig. S7g
Vehicle
+
+
+
−
BTZ
+
−
+
−
−
+
−
+
AZD6738
GSDME-F
55 kDa
GSDME-N
35 kDa
GAPDH
35 kDa
19

## Slide 20
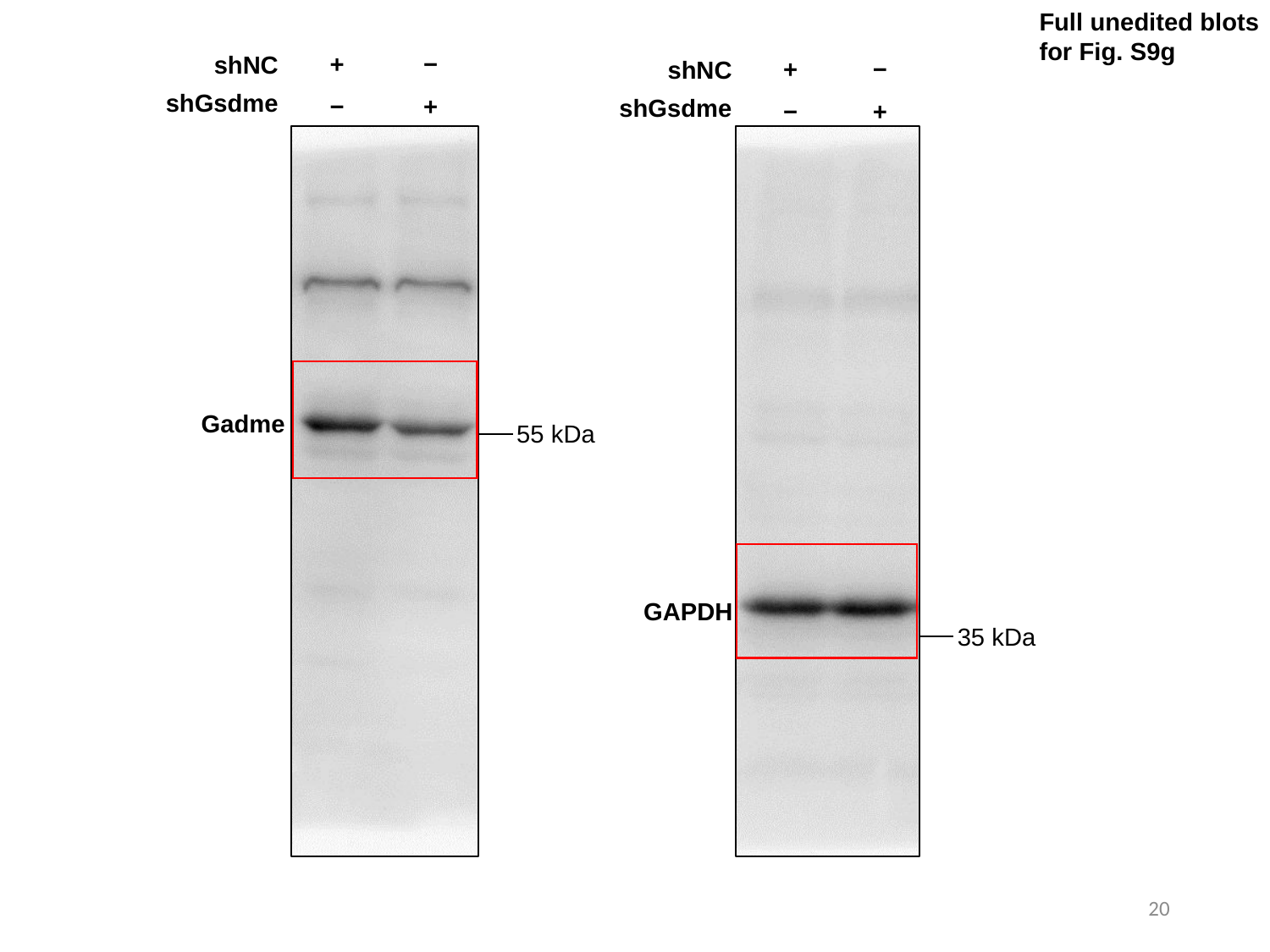

Full unedited blots
for Fig. S9g
+
−
shNC
+
−
shNC
shGsdme
−
+
shGsdme
−
+
Gadme
55 kDa
GAPDH
35 kDa
20

## Slide 21
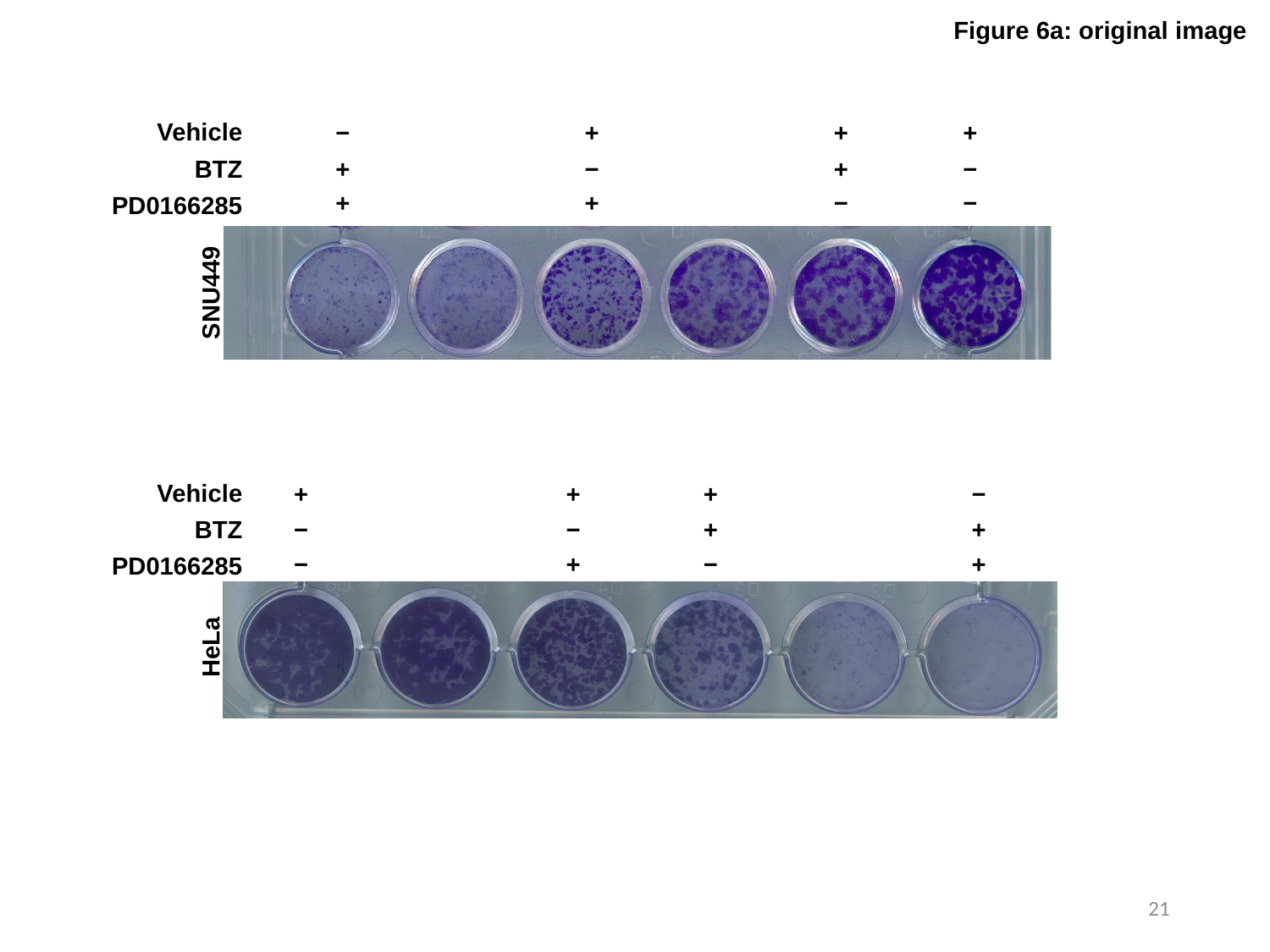

Figure 6a: original image
Vehicle
−
+
+
+
BTZ
+
−
+
−
+
+
−
−
PD0166285
SNU449
Vehicle
+
+
+
−
BTZ
−
−
+
+
−
+
−
+
PD0166285
HeLa
21

## Slide 22
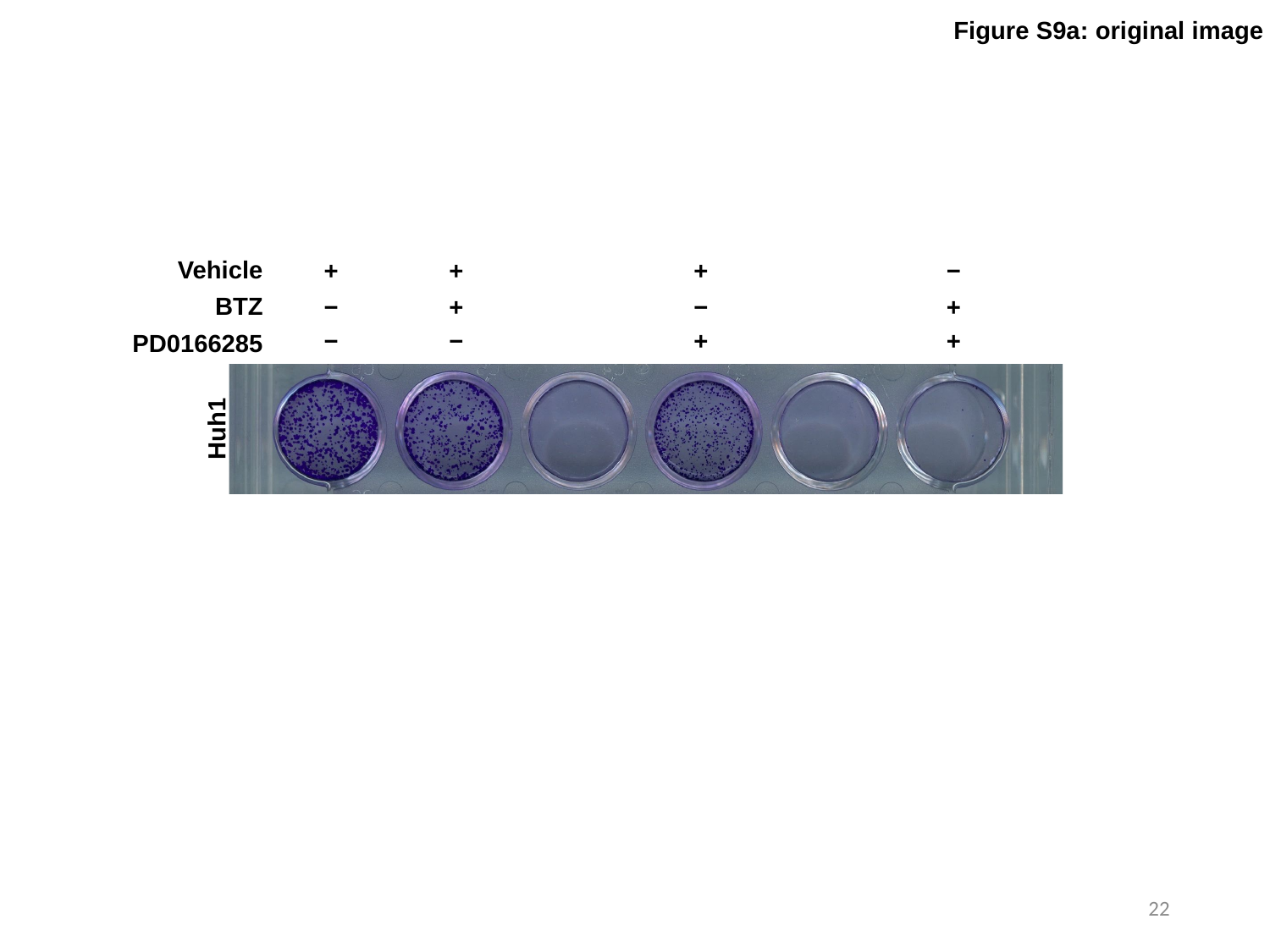

Figure S9a: original image
Vehicle
+
+
+
−
BTZ
−
+
−
+
−
−
+
+
PD0166285
Huh1
22

## Slide 23
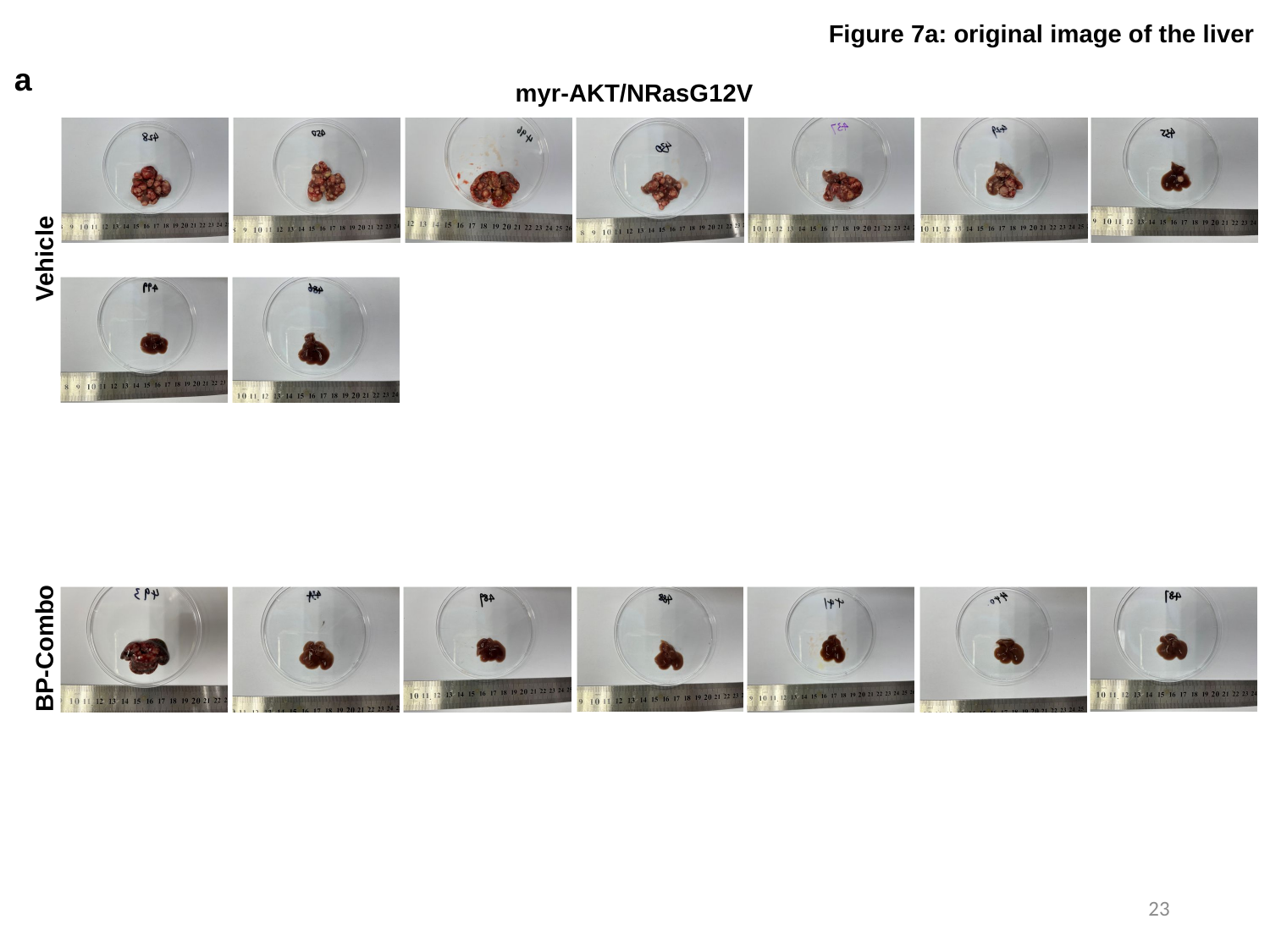

Figure 7a: original image of the liver
a
myr-AKT/NRasG12V
Vehicle
BP-Combo
23

## Slide 24
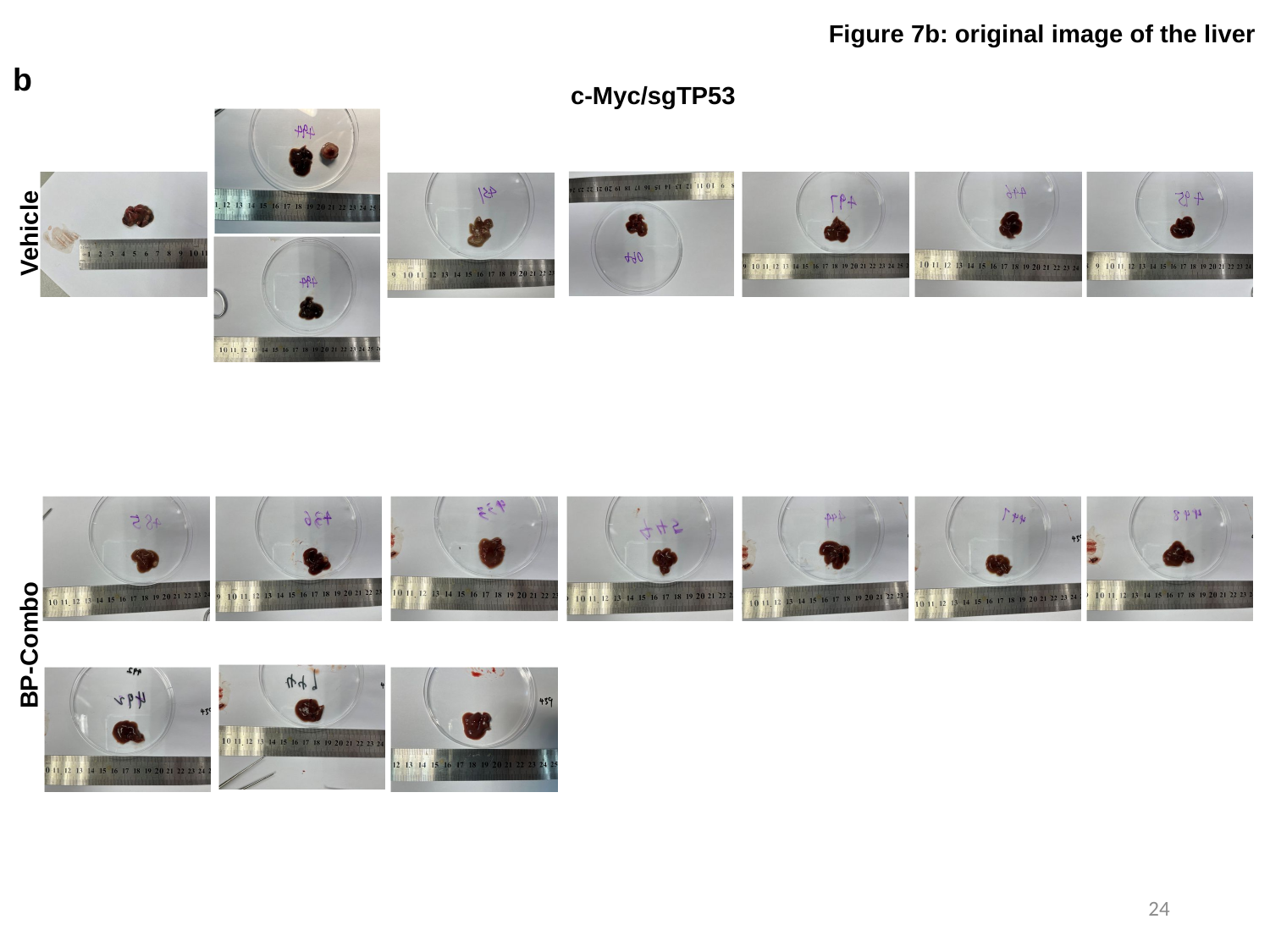

Figure 7b: original image of the liver
b
c-Myc/sgTP53
Vehicle
BP-Combo
24

## Slide 25
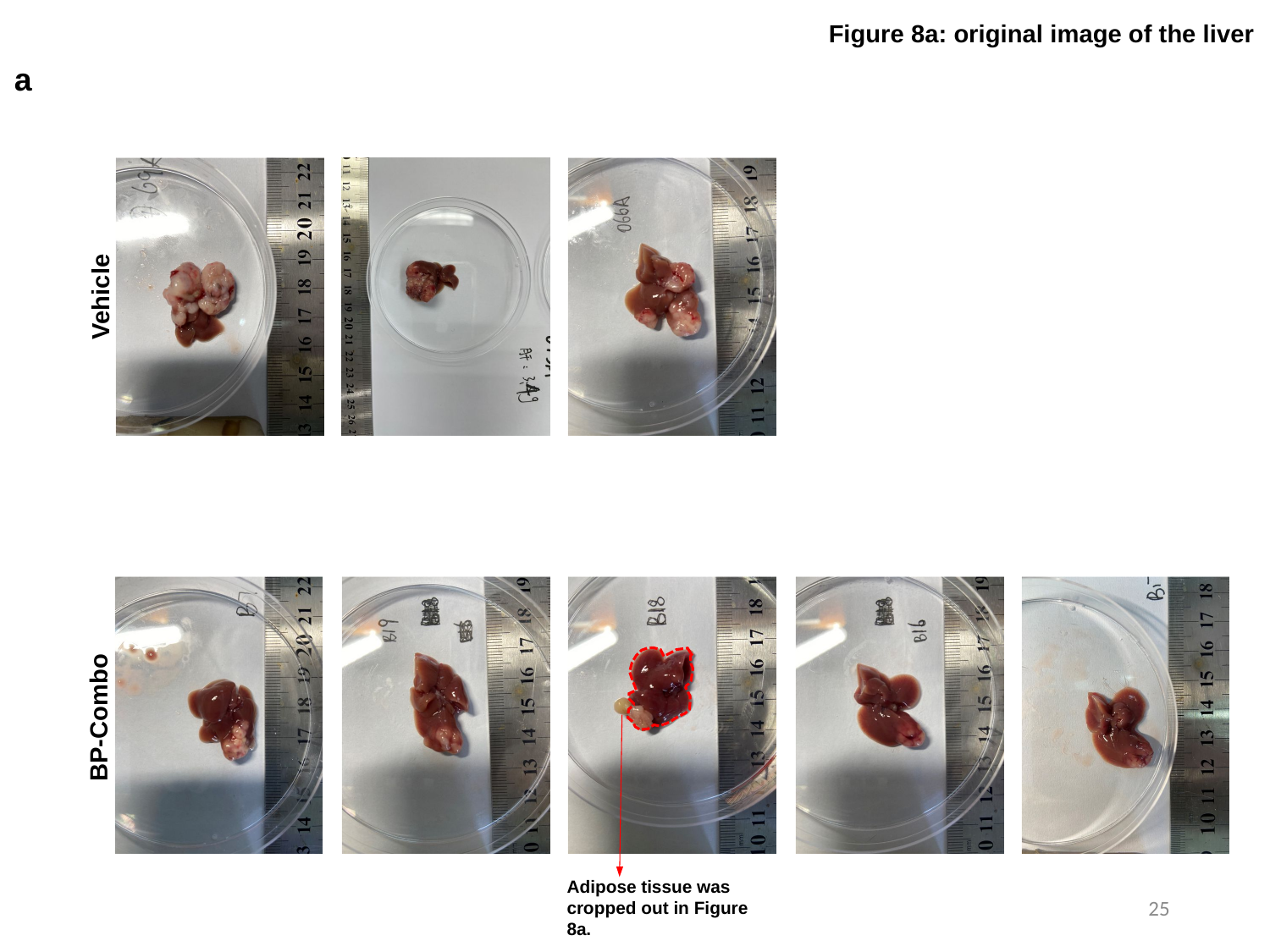

Figure 8a: original image of the liver
a
Vehicle
BP-Combo
Adipose tissue was cropped out in Figure 8a.
25
